# BAMLET kills chemotherapy-resistant mesothelioma cells, holding oleic acid in an activated cytotoxic state

**DOI:** 10.1371/journal.pone.0203003

**Published:** 2018-08-29

**Authors:** Emma M. Rath, Yuen Yee Cheng, Mark Pinese, Kadir H. Sarun, Amanda L. Hudson, Christopher Weir, Yiwei D. Wang, Anders P. Håkansson, Viive M. Howell, Guo Jun Liu, Glen Reid, Robert B. Knott, Anthony P. Duff, W. Bret Church

**Affiliations:** 1 Faculty of Pharmacy, University of Sydney, Sydney, NSW, Australia; 2 Asbestos Diseases Research Institute (ADRI), Concord, NSW, Australia; 3 University of Sydney, Sydney, NSW, Australia; 4 Kinghorn Cancer Centre and Garvan Institute of Medical Research, Sydney, NSW, Australia; 5 Bill Walsh Translational Cancer Research Laboratory, Kolling Institute, University of Sydney, Sydney, NSW, Australia; 6 Northern Blood Research Centre, Kolling Institute, University of Sydney, Sydney, NSW, Australia; 7 Burns Research, ANZAC Research Institute, Concord Hospital, University of Sydney, Concord, NSW, Australia; 8 Division of Experimental Infection Medicine, Lund University, Lund, Sweden; 9 Australian Nuclear Science and Technology Organisation (ANSTO), New Illawarra Rd, Lucas Heights, NSW, Australia; 10 Brain and Mind Centre and Faculty of Health Sciences, University of Sydney, Sydney, NSW, Australia; Columbia University, UNITED STATES

## Abstract

Malignant pleural mesothelioma is an aggressive cancer with poor prognosis. Here we have investigated in vitro efficacy of BAMLET and BLAGLET complexes (anti-cancer complexes consisting of oleic acid and bovine α-lactalbumin or β-lactoglobulin respectively) in killing mesothelioma cells, determined BAMLET and BLAGLET structures, and investigated possible biological mechanisms. We performed cell viability assays on 16 mesothelioma cell lines. BAMLET and BLAGLET having increasing oleic acid content inhibited human and rat mesothelioma cell line proliferation at decreasing doses. Most of the non-cancer primary human fibroblasts were more resistant to BAMLET than were human mesothelioma cells. BAMLET showed similar cytotoxicity to cisplatin-resistant, pemetrexed-resistant, vinorelbine-resistant, and parental rat mesothelioma cells, indicating the BAMLET anti-cancer mechanism may be different to drugs currently used to treat mesothelioma. Cisplatin, pemetrexed, gemcitabine, vinorelbine, and BAMLET, did not demonstrate a therapeutic window for mesothelioma compared with immortalised non-cancer mesothelial cells. We demonstrated by quantitative PCR that ATP synthase is downregulated in mesothelioma cells in response to regular dosing with BAMLET. We sought structural insight for BAMLET and BLAGLET activity by performing small angle X-ray scattering, circular dichroism, and scanning electron microscopy. Our results indicate the structural mechanism by which BAMLET and BLAGLET achieve increased cytotoxicity by holding increasing amounts of oleic acid in an active cytotoxic state encapsulated in increasingly unfolded protein. Our structural studies revealed similarity in the molecular structure of the protein components of these two complexes and in their encapsulation of the fatty acid, and differences in the microscopic structure and structural stability. BAMLET forms rounded aggregates and BLAGLET forms long fibre-like aggregates whose aggregation is more stable than that of BAMLET due to intermolecular disulphide bonds. The results reported here indicate that BAMLET and BLAGLET may be effective second-line treatment options for mesothelioma.

## Introduction

Malignant pleural mesothelioma is an aggressive tumour of the membrane lining the pleural cavity of the chest caused by exposure to asbestos fibres [1–3]. Due to heavy use of asbestos in the past, the United States, European countries, and Australia are suffering high incidence rates of mesothelioma, and the incidence is rising in developing nations where asbestos mining and use remains unrestricted, estimated as approximately 43 000 annual deaths worldwide of which 13% are in Asia [1–2,4–5]. Treatment options for mesothelioma are mainly palliative in nature, and patients will be confronted with recurrence of disease and drug resistance. The chemotherapy treatment of cisplatin plus pemetrexed was adopted as the standard first-line chemotherapy treatment when it increased the average survival of advanced mesothelioma patients from 9 to 12 months [6,7]. Other chemotherapies that have shown treatment benefit include gemcitabine and vinorelbine [8]. After initial chemotherapy treatment, mesothelioma almost always progresses [7] and as yet, there is no effective second-line chemotherapy [7–9]. There is therefore an urgent unmet need for treatment options for this treatment-resistant cancer.

Complexes of oleic acid with bovine α-lactalbumin protein (BAMLET/HAMLET–Bovine/Human Alpha-lactalbumin Made LEthal to Tumours [10–11]) and with bovine β-lactoglobulin (BLAGLET–Beta-LActoGlobulin made LEthal to Tumours) have demonstrated broad-spectrum anti-cancer activity *in vitro* to over 50 cancer cell lines [12–21] inventoried in [22], and have shown efficacy in reducing tumours and non-toxicity to healthy tissue in a few *in vivo* experiments of cancer tumours in humans, mice, and rats [13,15,23–25]. HAMLET and BAMLET are also cytotoxic towards some bacteria *in vitro* and *in vivo* in mice [26–29]. HAMLET and BAMLET complexes have not yet been tested on mesothelioma cancer cells.

Ever since the first published work on HAMLET that created the BAMLET field of study [12], researchers have been aware that BAMLET compounds are deactivated by components in blood, specifically as a consequence of both albumin [30] and calcium [31] sequestering the oleic acid. Taking the cue from that first study, cell viability assays are generally performed in the absence of serum during the BAMLET incubation step. We envisage administration of BAMLET directly into the pleural cavity to treat mesothelioma. However, blood components are also not completely absent in the pleural cavity and albumin and calcium can also be present due to pleural effusion.

It has been shown that the fatty acid, most commonly oleic acid, is the main active component of BAMLET and HAMLET-like complexes [14,18]. However, the protein component also plays an important role in BAMLET activity, as not only are a range of anti-cancer and anti-bacterial activities observed for BAMLETs prepared by different methods, but also in BAMLETs constituted with different proteins (reviewed in [22]). A striking example is albumin which binds oleic acid in blood without reports of associated anti-cancer activity [32], yet forms a cytotoxic BAMLET complex with oleic acid when prepared using a BAMLET heating protocol [33]. Uncertainty remains as to whether oleic acid is the lone cytotoxic component of BAMLET or if the protein component also contributes to this activity. Concentrations of oleic acid alone were as efficient at killing cells as the BAMLET-like complexes [14,34–35]. Alternate studies showed that similar amounts of oleic acid alone killed no cells or fewer cells than did the BAMLET-like complexes, and that higher amounts of oleic acid or sodium oleate were required to kill the same number of cells [18,27,33,36–39,40–43]. Researchers using α-lactalbumin without oleic acid as a control for *in vitro* HAMLET and BAMLET experiments have found no toxicity of α-lactalbumin towards cancer cells [14,24,27,44–46], whereas others found a reduction in cell viability of approximately 10% [12,15,39]. α-Lactalbumin protein treatment used as a control without oleic acid increased *in vivo* survival of bladder cancer tumour-bearing rats, although 5 times more α-lactalbumin was required to produce a similar level of survival as BAMLET treatment [15].

What the specific cellular targets of BAMLET are is an open question and is an active area of investigation (reviewed in [22]). The many proposed cellular targets include membrane ion channels [29,47–50], histones [51–53], mitochondria [54–56], proteasomes [19], α-actinin [57], annexin [30], and ATP synthase [58]. Multiple lines of evidence indicate that the initial BAMLET target is the cellular membrane of cancer cells [30,43,59–65].

It is generally accepted that the cytotoxicity of BAMLET-like anti-cancer complexes is due to a structural balance of its components, the protein and the oleic acid that can be accommodated by the protein, resulting in release of the oleic acid component to the cell membrane, termed “cargo off-loading” [66–68]. The novel protein-lipid structure of BAMLET-like complexes, in an aqueous environment will hold a nanoscale droplet of oleic acid oil in a cytotoxic “solubilised” state [68], so called because when the complex comes into contact with lipid membranes, the oleic acid dissociates from the protein complex and associates with the cell membrane [65]. This behaviour is in contrast to the unreactive behaviour of oleic acid in the bulk phase of a macroscale droplet of oil. The cytotoxicity mechanism can be thought of as a balance in stability of the structure of the BAMLET-like complexes; the complexes appear stable in a polar environment and are unstable when in the presence of lipids or strongly lipid-binding blood components. Such a mechanism is not used by any current cancer chemotherapies, targeted therapy drugs, or immunotherapies. The structure of BAMLET-like compounds represents a recently described type of lipoprotein structure [69] that has been named the “liprotide” [33]. This structure is well characterised by SAXS, with the SAXS curves of liprotides having distinctive features, in particular, a blunt first minimum followed by a diffuse second maximum.

In summary, BAMLET compounds have broad-spectrum anti-cancer activity and mesothelioma is a cancer in need of a second-line treatment, and thus the present work investigates BAMLET compounds as a potential therapy for mesothelioma, with suitable controls. The general BAMLET shortcoming that it is disabled by blood is not anticipated to be problematic in the case of mesothelioma treatment as the compound could be administered directly to the pleural cavity where blood is not abundant. This study investigates BAMLET activity towards mesothelioma cells in the presence of non-abundant blood compounds, and towards mesothelioma cells that have being treated with and have developed resistance to chemotherapies, and also determines the toxicity of α-lactalbumin alone. A bioinformatics analysis of membrane proteins in bacterial genomes is carried out for clues as to the BAMLET target in both bacteria and tumour cells, as simplified analysis is required for bacterial genomes compared with eukaryotic genomes. Results point to the membrane-embedded enzyme ATP synthase as a possible BAMLET target and thus gene expression alterations of ATP synthase subunits in mesothelioma cells treated with BAMLET are investigated. Given that the novel BAMLET mechanism of activity is due to its novel structure, this study carries out structural characterisations that explore the relationship between structure and anti-cancer activity, by characterising and comparing BAMLET and BLAGLET structures by small angle X-ray scattering (SAXS), circular dichroism (CD), and scanning electron microscopy (SEM).

## Results

### BAMLET and BLAGLET are cytotoxic to human mesothelioma cell lines as a function of oleic acid content

**Fig 1:**
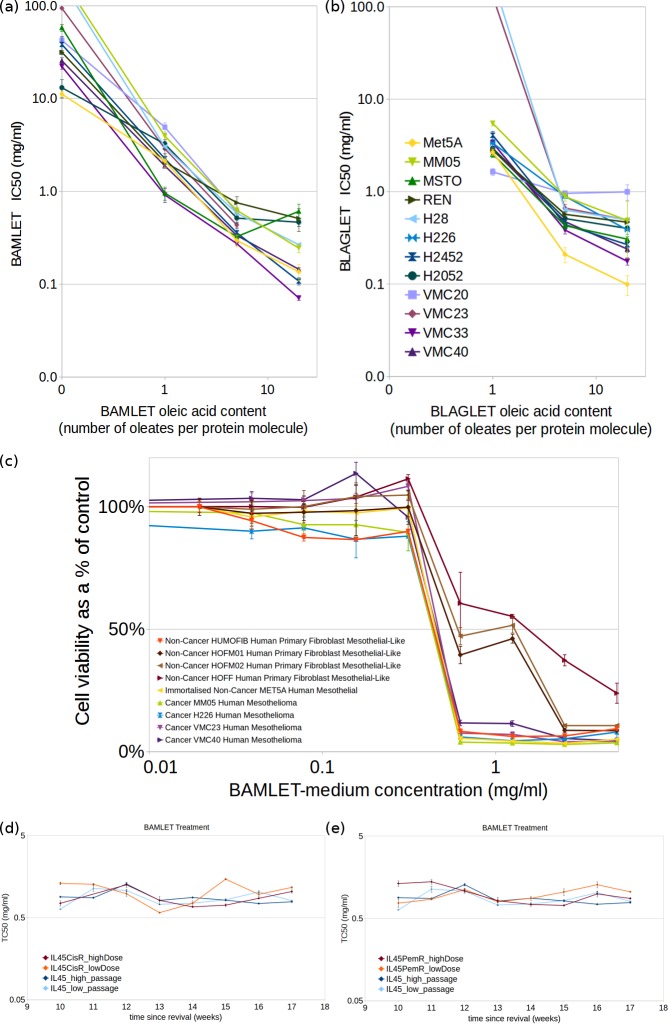
TC_50_ values at which 50% of human mesothelioma and non-cancer cells remain viable showing that the higher the amount of oleic acid in the BAMLET or BLAGLET compound, the more sensitive are cells to treatment. (a) BAMLET or bovine α-lactalbumin for 4.5 hours in medium without FBS after removal of conditioned FBS-containing medium, then addition of FBS and incubation for 1 day; (b) BLAGLET for 4.5 hours following same protocol as in (a). Cell lines shown are human immortalised non-cancer mesothelial (Met5A, yellow) and human mesothelioma (MM05, MSTO, REN, H28, H226, H2452, H2052, VMC20, VMC23, VMC33, and VMC40, colours as shown in (b)). Dose-response curves for a representative experiment are shown in Figures Aa and Ab in S1 File. (c) Cell death assay plot of human non-cancer primary fibroblast cells and human mesothelioma cells lines treated with BAMLET for 3 days with FBS present. Mesothelioma cells and immortalised non-cancer cells have a similar, consistent response to BAMLET (TC_50_ 0.7±0.1 mg/ml for MM05, H226, VMC23, VMC40, Met5A, and Humofib). There is a biphasic response to BAMLET treatment for three non-cancer primary cells (HOFF, HOFM01 and HOFM02) which manifests as two steps in the dose-response curve (as opposed to one step in the dose-response curve for the mesothelioma cells) and two values of TC_50_ (first value of TC_50_ is same as for mesothelioma cells and second value of TC_50_ is 2.4±0.3, 1.8±0.7, and 1.7±0.5 mg/ml for HOFF, HOFMO1, and HOFMO2 respectively and are statistically significantly different to the first TC_50_ value). Experiments were carried out 3 times with 3 replicates per experiment, and values shown are from a representative experiment consisting of three replicates. (d,e) BAMLET TC_50_ values for chemotherapy-resistant and chemotherapy-sensitive cells, showing that both types of cells have the same level of sensitivity to BAMLET. Although pemetrexed-resistant or cisplatin-resistant cells are more resistant to those chemotherapies (Figure C in S1 File), they are just as sensitive to BAMLET. There is variability in TC_50_ from week to week, which we attribute to the regular dosing with maintenance chemotherapy between assays and high passage number. However, statistical consistency of effects tests conclude that there is no overall difference in TC_50_ between the chemo-resistant and parental cells. (d) Pemetrexed-resistant (IL45-PemR, red and orange lines for cells maintained with regular high and low pemetrexed doses respectively) and (e) cisplatin-resistant (IL45-CisR, red and orange lines for cells maintained with regular high and low cisplatin doses respectively) rat mesothelioma cells lines and their parental chemosensitive rat mesothelioma cell line (IL45, blue and cyan lines for high- and low-passage number cells respectively) treated with BAMLET-medium (BAMLET prepared with oleic acid-protein ratio of 5 to 1).

The cytotoxicity of BAMLET (Fig 1A) and BLAGLET (Fig 1B) compounds having low, medium and high levels of oleic acid were tested on a range of human mesothelioma cell lines: MM05, MSTO, REN, H28, H226, H2452, H2052, VMC20, VMC23, VMC33 and VMC40 (see Methods). After 4.5 hours treatment of BAMLET or BLAGLET, in the absence of serum (to avoid deactivation of treatment compound by blood components), followed by 1 day incubation in FBS, these mesothelioma cells were found to be sensitive to BAMLET and BLAGLET at similar doses. The toxic concentration TC_50_ was in the range 0.3 to 0.8 mg/ml for treatment with the BAMLET-medium species and 0.4 to 1.0 mg/ml for BLAGLET-medium. The higher the oleic acid content of BAMLET or BLAGLET, the more sensitive the mesothelioma cells to the compound (TC_50_ values were lower for BAMLET-high and BLAGLET-high being in the range 0.07 to 0.6 mg/ml and 0.2 to 1.0 mg/ml respectively; TC_50_ values were higher for BAMLET-low and BLAGLET-low being in the range 1.0 to 5.0 mg/ml and 1.6 to 5.5 mg/ml respectively). An immortalised non-cancer human mesothelial cell line (MeT5A) was used as the non-cancer control due to ease of growing large quantities necessary for the assays, and showed similar sensitivities to BAMLET and BLAGLET as the mesothelioma cell lines (Met5A TC_50_ of 0.3±0.04 mg/ml and 0.2±0.04 mg/ml for BAMLET-medium and BLAGLET-medium respectively). Viability assays for treatment with α-lactalbumin alone (no oleic acid) with concentrations up to 39 mg/ml demonstrated TC_50_ values ranging from 13 to 38 mg/ml for some human mesothelioma cell lines, whereas for others the TC_50_ was not reached but viability did decrease and TC_50_ can be extrapolated to 58 mg/ml and higher. (Table A in S1 File is a complete compilation of TC_50_ for all human cells.)

### Non-cancer primary fibroblasts are more resistant to BAMLET than cancer cells when serum is present

Non-cancer primary human fibroblast cells, considered to be more representative of healthy mesothelial cells than immortalised cell lines, were assayed to measure BAMLET toxicity towards healthy-like cells compared to mesothelioma cells. The BAMLET species assayed was BAMLET-medium, prepared with a 5-to-1 molar ratio of oleic acid-to-protein, similar to the ratios of species used in reported *in vivo* experiments [13–15,23,25]. For three of the four non-cancer primary human fibroblast control cell lines tested, the response to BAMLET was biphasic, with a large proportion of the non-cancer cells more resistant to BAMLET than were the mesothelioma cells (statistically significantly higher TC_50_ of 2.4±0.3, 1.8±0.7, and 1.7±0.5 mg/ml for HOFF, HOFM01, and HOFM02 respectively, and percentage of resistant cells was 71%, 51%, and 43% respectively; respective p-values demonstrating statistical significance compared with mesothelioma MM05 are 2e-11, 9e-14, and 5e-13), with the rest of the non-cancer cells having the same sensitivity to BAMLET as the mesothelioma cells (Fig 1C; Table A in S1 File; and intermediate results interspersed in the code of S1 Dataset). Treatment consisted of three days incubation of cells with BAMLET-medium in the presence of 10% fetal bovine serum (FBS), to maintain both types of cells with uninterrupted supply of nutrients needed to stay healthy. Visual inspection did not reveal there to be two different types of cells in the cultures (Figure B in S1 File). We note that the three non-cancer fibroblast cell lines that exhibited resistance to BAMLET were established only a few months prior to the experiments, whereas the non-cancer fibroblast cell line that had similar sensitivity to BAMLET as the mesothelioma cell lines (Humofib; TC_50_ 0.7±0.1 mg/ml, response to BAMLET was not biphasic and no proportion of cells were more resistant to BAMLET), established by the same method, had been propagated for approximately 4 years prior to the experiments, demonstrating an immortalised characteristic of indefinite proliferation. These results suggest that cells become more sensitive to BAMLET when they become immortalised. Although not investigated further, we note that the three BAMLET-resistant non-cancer fibroblast cell lines grew very slowly *in vitro*, and after several more months of propagation, their proliferation increased to similar easy-to-culture levels as for Humofib, at which point they became similarly sensitive to BAMLET. This suggests that BAMLET's effects are growth phase-dependent. In addition to showing that BAMLET is more toxic to mesothelioma cells and immortalised cells than to recently established non-cancer healthy-like cell lines, these results show that BAMLET is able to kill mesothelioma cells when serum is present. When serum was removed prior to BAMLET dosing, the mesothelioma cells were approximately 7 to 10 times more sensitive to BAMLET (eg. TC_50_ 0.05±0.02 mg/ml for MM05, 0.10±0.01 mg/ml for H226, Table A in S1 File) than when FBS was present (eg. TC_50_ 0.7±0.1 mg/ml for MM05, 0.70±0.1 mg/ml for H226, Table A in S1 File). We note that when FBS was not present during BAMLET incubation, the non-cancer primary fibroblasts did not have a biphasic response and were not more resistant to BAMLET than were mesothelioma cells (TC_50_ 0.10±0.001 mg/ml for HOFM01, 0.10±0.01 mg/ml for HOFM02, Table A in S1 File).

### BAMLET is as cytotoxic to cisplatin-resistant and other chemotherapy-resistant mesothelioma cells as to chemotherapy-sensitive cells

The cisplatin-resistant rat mesothelioma cell line (IL45-CisR) had similar sensitivities to BAMLET as the parental cells (IL45) (Fig 1D; Table B in S1 File average IL45-CisR TC_50_ = 0.87±0.21 mg/ml; p-value = 1; results interspersed in the code of S1 Dataset). We established that the cisplatin-resistant cells did indeed have higher tolerances of cisplatin than the parental cells (Figure Ca in S1 File; average IL45-CisR TC_50_ = 3.18±1.90 μM, average IL45 TC_50_ = 1.74±0.31 μM; p-value = 0.0002). The pemetrexed-resistant rat mesothelioma cell line (IL45-PemR) had similar sensitivities to BAMLET as its parental pemetrexed-sensitive cells (IL45) in 22 out of 32 experiment comparisons (Fig 1E; Table B in S1 File average IL45-PemR TC_50_ = 1.00±0.27 mg/ml and average IL45 TC_50_ = 0.88±0.18 mg/ml; p-value = 1). We found that the pemetrexed-resistant cells were indeed resistant to pemetrexed at the doses tested (IL45-PemR TC_50_ was not reached for most experiments; p-value = 1e-72) while its parental IL45 cell line was sensitive to pemetrexed (Figure Cc in S1 File; Table B in S1 File average IL45 TC_50_ = 8.00±7.07 μM). The rat mesothelioma cell line developed for vinorelbine resistance (IL45-VLBR) had similar sensitivities to BAMLET as the parental cells (IL45) (Figure Cg in S1 File; Table B in S1 File average IL45-VLBR TC_50_ = 0.93±0.27 mg/ml; p-value = 1) although in our hands we did not achieve consistently higher resistance to vinorelbine (data variable from week to week and not shown). The gemcitabine-resistant rat mesothelioma cell line (IL45-GemR) had higher tolerances to gemcitabine than the parental (IL45) cells (Figure Ce in S1 File; average IL45-GemR TC_50_ = 1.43±1.404 μM, average IL45 TC_50_ = 0.46±0.35 μM; p-value = 4e-102). However, unlike the trend seen with the other three chemotherapies, the gemcitabine-resistant cells were also more resistant to BAMLET than the parental cells (Figure Cf in S1 File; Table B in S1 File average IL45-GemR TC_50_ = 1.23±0.76 mg/ml; p-value = 1e-5). The immortalised non-cancer control rat mesothelial cell line (4/4RM.4) had similar sensitivity to BAMLET (average BAMLET TC_50_ = 0.84±0.08 mg/ml) as the rat mesothelioma IL45 cell line (Table B in S1 File). These results indicate that BAMLET is as effective in cytotoxicity towards mesothelioma cells that have been treated with and have developed resistance to the cisplatin, pemetrexed, and vinorelbine chemotherapies currently used to treat mesothelioma, as it is towards chemotherapy-naive mesothelioma cells. These results also indicate that mesothelioma cells that have developed resistance to gemcitabine (another chemotherapy used to treat mesothelioma) may be more resistant to BAMLET.

### Immortalised non-cancer mesothelial cell lines are as sensitive to chemotherapies as mesothelioma cells

The immortalised non-cancer control human mesothelial cell line (MeT5A) had similar sensitivity to cisplatin, pemetrexed, gemcitabine, or vinorelbine as the human mesothelioma cells (Figures Da to Dd in S1 File). The immortalised non-cancer rat mesothelial cell line (4/4RM.4) also showed similar or increased sensitivity to cisplatin, pemetrexed, and gemcitabine compared to the rat mesothelioma cells (Table B in S1 File). The long-time immortalised non-cancer primary fibroblast human cell line (Humofib) was just as sensitive to cisplatin or pemetrexed treatment as the human mesothelioma cell lines (Figures Da to Dd in S1 File).

### Bioinformatics analysis of bacterial sequences indicates plasma membrane ATP synthase as a potential BAMLET target

A comparison of the membrane proteins of HAMLET-sensitive (*Streptococcus pneumoniae*, *Streptococcus mitis*, and *Streptococcus pyogenes* [26]) and HAMLET-resistant bacteria (*Escherichia coli*, *Staphylococcus aureus*, *Staphylococcus epidermidis*, *Klebsiella pneumoniae*, *Pseudomonas aeruginosa*, and *Enterobacter cloach* [26]) reveals that the difference between the two groups is the HAMLET-resistant bacteria possess the F0F1 ATP synthase subunit I gene whilst the HAMLET-sensitive bacteria do not (see Figure E in S1 File for relative gene placements and subunit I sequence alignment). For all six HAMLET-resistant bacteria, the Interproscan [70] search of the protein sequence of the gene preceding membrane subunits A and C matches it to subunit I (Interproscan ID IPR005598) and predicts it to possess four transmembrane helices identifying it as a transmembrane protein. Subunit I has been shown to assist in assembly of the membrane-embedded c-ring of F0F1 ATP synthase [71–72], a major component of the ATP synthase proton pore [73]. BLASTP searches confirmed that ATP synthase subunit I is not present in the HAMLET-sensitive bacteria nor in the human genome (all six subunit I sequences of the HAMLET-resistant bacteria were used for BLASTP searches). All BLASTP searches returned a result of no significant similarity found, with the exception of three sequenced *S*. *pneumoniae* isolates (identified in NCBI protein database as CVY60247, COE43039, and CJL04251). The NCBI nucleotide database contains 1,274,592 species and isolates of *S*. *pneumoniae* (for search term: “Streptococcus pneumoniae” AND “genome”), of which only these three contain the subunit I gene. Thus, *S*. *pneumoniae* does not contain the ATP synthase subunit I gene. We thus hypothesised that the ATP synthase c-ring membrane proton pore component functioning without the presence nor assistance of subunit I may be implicated in BAMLET's membrane depolarisation activity and anti-cancer mechanism. Given this result and given that other ATP synthase subunits may be targeted by HAMLET [58], we further investigated expression of ATP synthase genes in response to regular BAMLET dosing.

### Quantification by RT-qPCR of ATP synthase genes indicates that mesothelioma cells decrease their metabolic activity in response to regular BAMLET dosing

**Fig 2:**
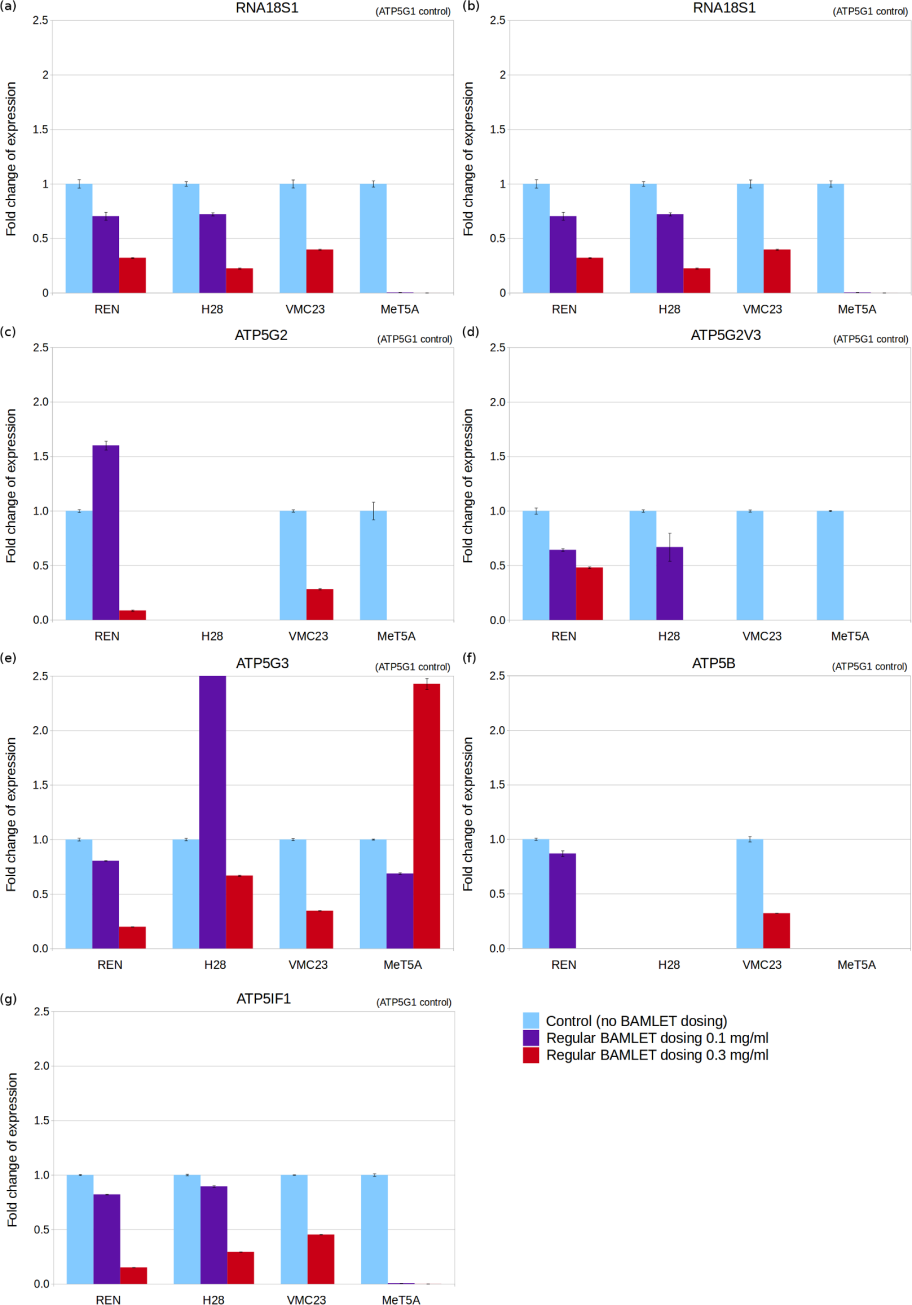
Fold change of mRNA and rRNA expression for mesothelioma (REN, H28, and VMC23) and control (MeT5A) cells regularly dosed with low or high BAMLET doses. Fold change measured for 3 replicates by qPCR for genes RNA18S ribosomal 1 (a), ATP synthase subunit 3 isoform 1 variant 2 (b), ATP synthase subunit 3 isoform 2 (c), ATP synthase subunit 3 isoform 2 variant 3 (d), ATP synthase subunit 3 isoform 3 (e), ATP synthase subunit beta (f), and ATP synthase inhibitory factor 1 (g).

The gene chosen as a standard for normalisation of qPCR results is ATP synthase subunit c isoform 1 (ATP5G1) as it was more stable across control and BAMLET-dosed cell lines than the housekeeping RNA18S ribosomal 1 gene (RNA18S1) (Figure F in S1 File). The regular high BAMLET dosing (0.3 mg/ml) was with sub-lethal concentrations lower than the TC_50_ concentration. Our results found that the higher the BAMLET dosing, the greater the downregulation of RNA18S1 as measured by fold change in ribosomal RNA (rRNA) expression (Fig 2A). Regular low BAMLET dosing (0.1 mg/ml) resulted in downregulated RNA18S1 gene expression in all three cell lines tested of which one was statistically significant (MeT5A), and regular high BAMLET dosing resulted in downregulated RNA18S1 gene expression in all four cell lines tested of which two were statistically significant (H28 and REN) (Table C in S1 File). This indicates that mesothelioma cells slow down their metabolic rate in response to BAMLET treatment. Regular BAMLET dosing of the higher 0.3 mg/ml dose results in downregulation or ablation in mesothelioma cells of the ATP synthase subunits measured (Fig 2B to 2H). Twenty-three of the twenty-four tested combinations of ATP synthase gene in cell line pairs exhibited downregulation of which seven were statistically significant (encompassing all 4 cell lines tested) (Table C in S1 File). This indicates that regular high BAMLET dosing of mesothelioma cells slows down their ATP synthase-generated energy activity. In most cases, regular low BAMLET dosing also results in downregulation of ATP synthase components (Fig 2; Table C in S1 File). In a few cases (in H28 and REN mesothelioma) low BAMLET dosing leads to upregulation of some of the ATP synthase subunit c isoforms which indicates that low BAMLET dosing may increase energy production in these mesothelioma cells even though high dosing lead to a decrease in mRNA expression of these same and other energy production genes. These overall results of downregulation of genes involved in metabolic and energy production activities in response to regular high or low BAMLET dosing are consistent with the hypothesis that ATP synthase is a target of BAMLET.

### BAMLET and BLAGLET adopt increasingly BAMLET-like structural features as oleic acid content is increased, and BLAGLET aggregation is more stable than BAMLET aggregation

**Fig 3:**
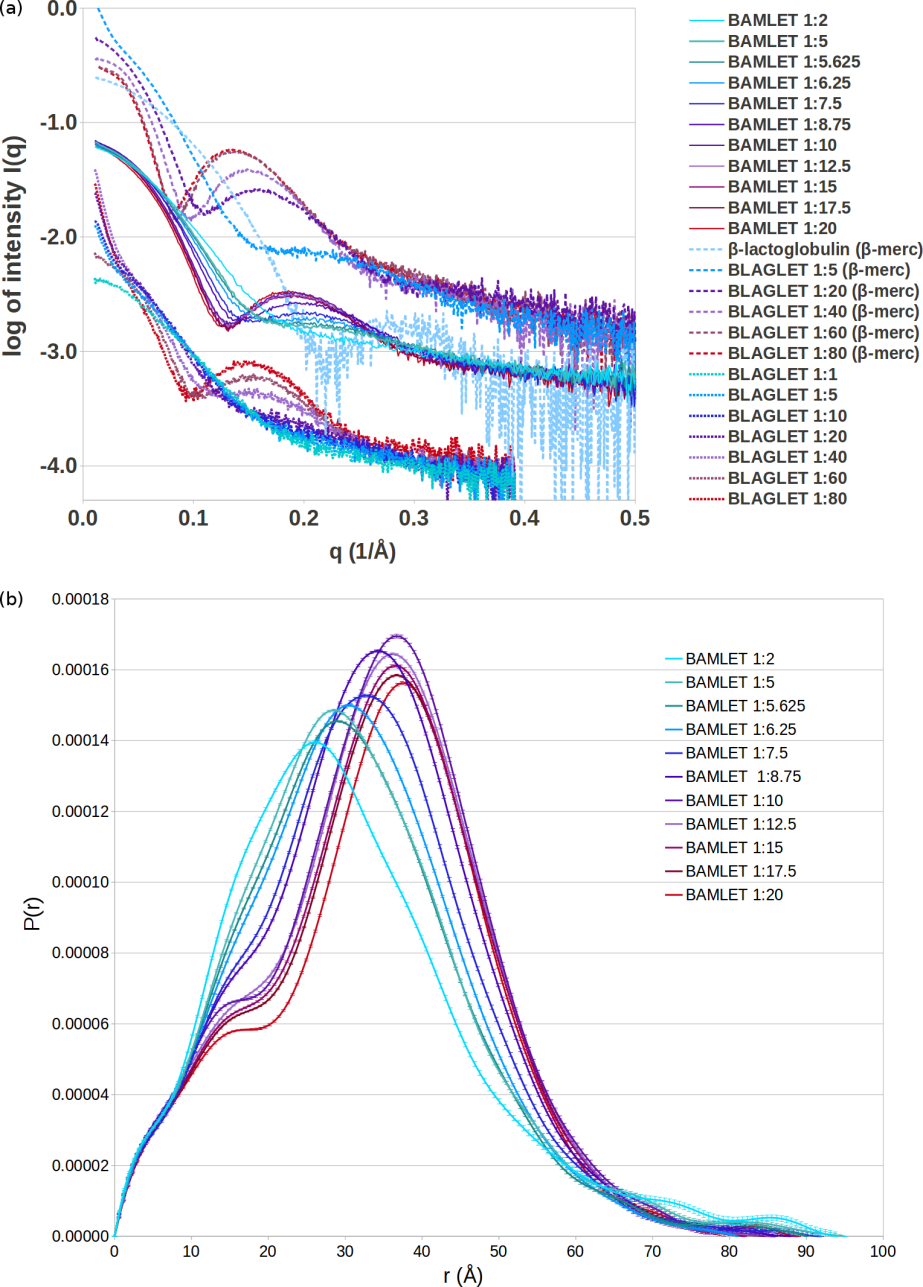
Experimental scattering curves and probability distribution profiles. (a) Experimental scattering curves demonstrating that BLAGLET exhibits the diffuse minimum followed by an increase in intensity (a “hump”) that is characteristic of BAMLET structures. Scattering shown is for β-lactoglobulin and BLAGLET species having protein:oleic acid input ratios of 1:5, 1:20, 1:40, 1:60, and 1:80, in pH 12 with 2% β-mercaptoethanol solution at 10°C (top), BAMLET species having input protein to lipid molecular ratios of 1:2, 1:5, 1:5.625, 1:6.25, 1:7.5, 1:8.75, 1:10, 1:12.5, 1:15, 1:17.5, and 1:20, in pH 12 solution at 4°C (middle), and BLAGLET species having protein:oleic acid input ratios of 1:1, 1:5, 1:10, 1:20, 1:40, 1:60, and 1:80, in pH 12 solution at 4°C without β-mercaptoethanol (bottom). As oleic acid content is gradually increased, the curves gradually develop a maximum that then gradually shifts to a higher q (representing gradually shorter distances between scattering points). The BLAGLET maximum at 0.16 Å^-1^ for BLAGLET 1:20 (in β-mercaptoethanol) shifts to 0.13 Å^-1^ for BLAGLET 1:80, corresponding to distances 39.3 Å to 48.3 Å (d = 2π/q). The BAMLET maximum at 0.2 Å^-1^ for BAMLET 1:5.625 shifts to 0.185 Å^-1^ for BAMLET 1:20, corresponding to distances 31.4 Å to 40.0 Å. Even though significant aggregation of BLAGLET occurs when β-mercaptoethanol is not present, as evidenced by the increasing SAXS intensity at low q, the characteristic diffuse minimum and secondary maximum (the “hump”) are still evident. (b) Probability distribution profiles, P(r), for BAMLET species in pH 12, 4°C, derived from SAXS data by the Indirect Method [74–75] with the curves set to zero at zero and Dmax distances, and used to create models of BAMLET. The P(r) is a histogram of distances between scattering points (in this case, protein mass) in the complexes. Low and no oleic acid content BAMLETs have P(r) curves typical of globular proteins, with one maximum near the centre of the histogram. Medium and high oleic acid content BAMLET P(r) curves have the shoulder that is characteristic of liprotides.

The SAXS data for high oleic acid content BAMLET and BLAGLET species exhibit the now classical liprotide characteristics of a non-sharp first minimum and a well-developed secondary maximum (Fig 3) which indicate that the structure is that of a liprotide having an oleic acid core and disordered protein shell. The resulting probability distribution function features a shoulder followed by a peak, now characteristic of liprotide structures (Fig 3B and Figure G in S1 File). The SAXS data for low oleic acid content BAMLETs and BLAGLETs are those of a globular protein. As oleic acid content increases, the liprotide features are more pronounced. Data curves for the highest two concentrations of BAMLET overlap, and the two highest concentrations of BLAGLET in β-mercaptoethanol overlap, indicating that a saturation point of oleic acid content was reached and excess oleic acid that could not be incorporated into the protein was removed by dialysis resulting in the same final structure. It has already been shown that excess oleic acid is not present in liprotide curves [69]. At pH 12, BAMLET species are non-aggregated, as shown by the constant SAXS intensity as q approaches zero (Fig 3A and Figure D in S1 File). This indicates that the added charge to protein side chains at pH 12 is enough to provide repulsion so that BAMLET is no longer aggregated. At pH 12, BLAGLET remains aggregated, as shown by increasing intensity of SAXS curves as q approaches zero, with the exception of BLAGLET 1:1 that is not aggregated (Fig 3 bottom). Addition of 2% β-mercaptoethanol at pH 12 results in non-aggregated BLAGLET (constant SAXS intensity as q approaches zero) with the exception of BLAGLET 1:5 that remains aggregated (Fig 3 top and Figure G in S1 File). This deaggregation of BLAGLET in β-mercaptoethanol indicates that disulphide bonds contribute to aggregation of BLAGLET and that BLAGLET structure consists of significantly more disulphide bonds than BAMLET. The significant amount of BLAGLET disulphide bonds is certainly due to the β-lactoglobulin protein's odd number of cysteines. One cysteine per β-lactoglobulin protein molecule will not be paired in a disulphide bond with another cysteine in the same molecule and, when accessible, will become paired with a cysteine from another protein molecule. This is in contrast to the α-lactalbumin's even number of cysteines and the resulting lack of aggregation of BAMLET at pH 12. Concentration-adjusted SAXS curves of BAMLET overlap, demonstrating that the aggregation status is stable and not dependent on concentration. This is also the case for BLAGLET in β-mercaptoethanol, with the exception of the highest concentrations that exhibit a decrease in the intensity at low q due to intermolecular repulsion (Figure G in S1 File). Plots of derived radius versus concentration and of I(0)/concentration versus concentration are effectively concentration independent, demonstrating that BAMLET in pH 12 and BLAGLET in β-mercaptoethanol are stable particles that are neither aggregating nor repulsing (derived parameters appear in Table D in S1 File, Guinier plots to find radii in Figure H in S1 File, and plots of derived parameters versus concentration in Figure I in S1 File). Given that these species are not aggregated, we carried out modelling of the shapes of these structures from P(r) distributions derived from the SAXS data (distance distribution plots derived by Indirect Method [74–75] in Figure J in S1 File, structure models and goodness-of-fit plots in Figure K in S1 File.)

### BAMLET and BLAGLET at pH 12 consist of increasingly peripherally located protein as oleic acid content increases

**Fig 4:**
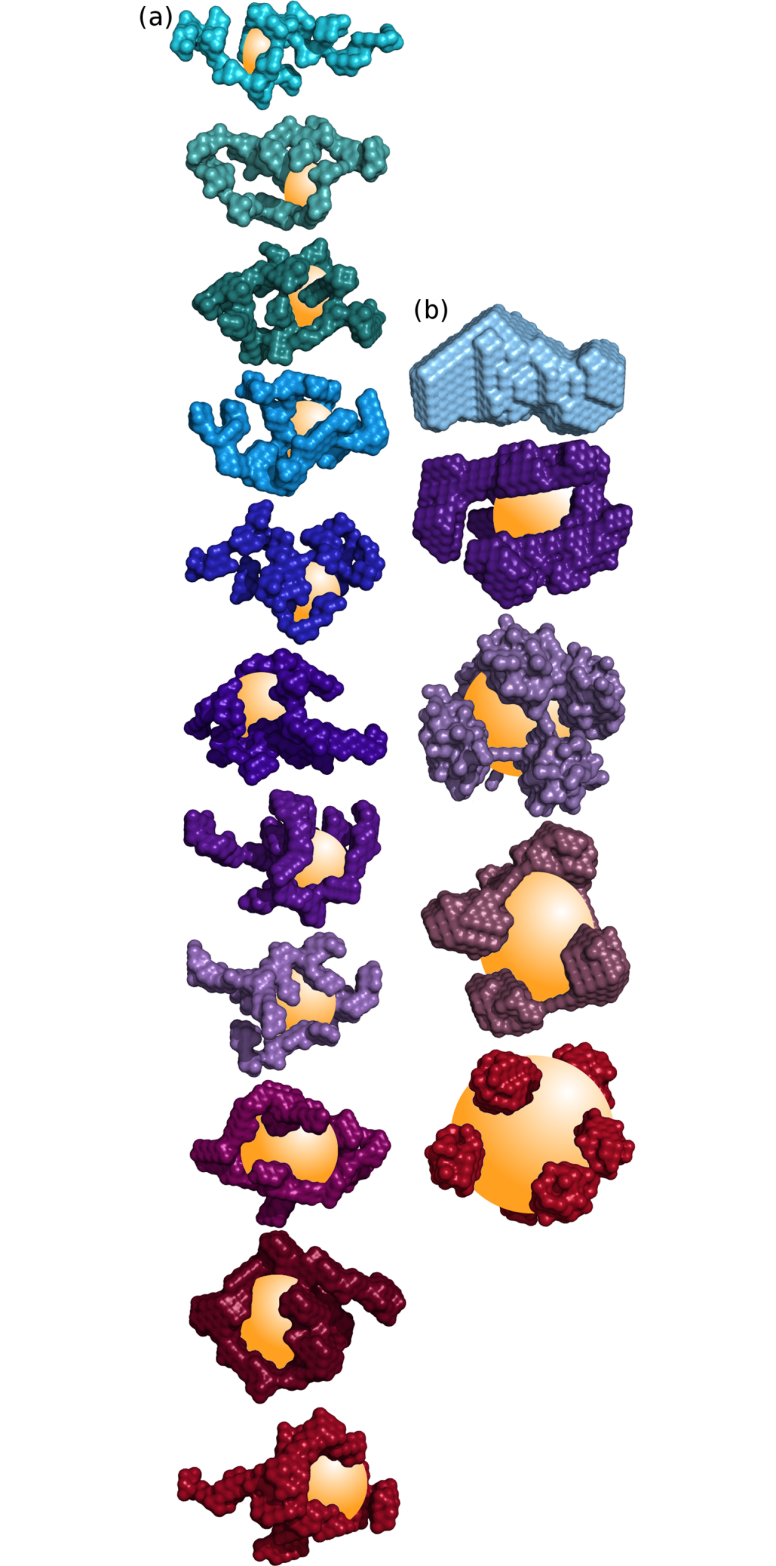
**SAXS-derived models, coloured as for SAXS curves in Fig 3, derived by DAMMIN** for (a) BAMLET species having (from top to bottom) input protein to lipid molecular ratios of 1:2, 1:5, 1:5.625, 1:6.25, 1:7.5, 1:8.75, 1:10, 1:12.5, 1:15, 1:17.5, and 1:20, and (b) β-lactoglobulin without oleic acid and BLAGLET having protein:lipid ratios of 1:20, 1:40, 1:60, and 1:80 (from top to bottom). The models are composed of dummy atoms that represent X-ray scattering mass of the protein component of BAMLET and do not represent the oleic acid. The speculatively modelled oleic acid oil droplets are depicted as a yellow sphere or ellipsoid in the centre of the partially unfolded protein model, as experimentally revealed in [68]. As oleic acid content increases, the models evolve from mildly elongated ellipsoid globular protein to spherical profile having peripheral protein density.

The structural units of BAMLET and BLAGLET species are similar to each other. (Fig 4 shows representative models; Figure K in S1 File shows multiple models; S2 Dataset contains all models; models contain dummy atoms representing scattering mass.) As oleic acid incorporated into BAMLET and BLAGLET species increases, the protein component is located more peripherally and the core volume not containing protein increases. Models for no or low oleic acid BAMLET and BLAGLET have globular protein structure. Models are more unfolded as oleic acid content is increased, with the high oleic acid content BAMLETs and BLAGLETs containing the bulk of the protein on the periphery of the model and not in the centre. The centre has been shown experimentally to contain a droplet of oleic acid oil [68], confirming previous predictions and modelling [33,69]. As oleic acid content of BAMLET increases, the mildly elongated shape envelope of low oleic acid content BAMLET becomes spherical for high oleic acid content BAMLET (seen visually in the models). As oleic acid content increases, BAMLET radius of gyration (R_g_) steadily increases while the maximum dimension of the complex in general decreases (Figure I in S1 File) confirming the visually observed elongated-to-spherical transition. This structural progression of decreasing maximum dimension and increasing R_g_ was previously mentioned [69] and is now convincingly demonstrated here for BAMLET by the SAXS modelling for a range of oleic acid content BAMLET complexes. This structural progression demonstrates how a fixed volume of α-lactalbumin protein incorporates increasing amounts of oleic acid in its core. In contrast, BLAGLET R_g_ steadily increases and maximum dimension remains constant as oleic acid content increases (Figure I in S1 File). Oleic acid may not be incorporated in BLAGLET protein in the same way as it is in BAMLET protein. There is an excellent fit between the simulated scattering from these models and the actual scattering data (shown by overlap of simulated and actual scattering curves in Figure K in S1 File) and the multiple models are similar (Figure K in S1 File), demonstrating that the models are the plausible representations of BAMLET and BLAGLET structure.

### Scanning electron microscopy shows that BAMLET forms rounded aggregates and BLAGLET forms long fibre-like aggregates

**Fig 5:**
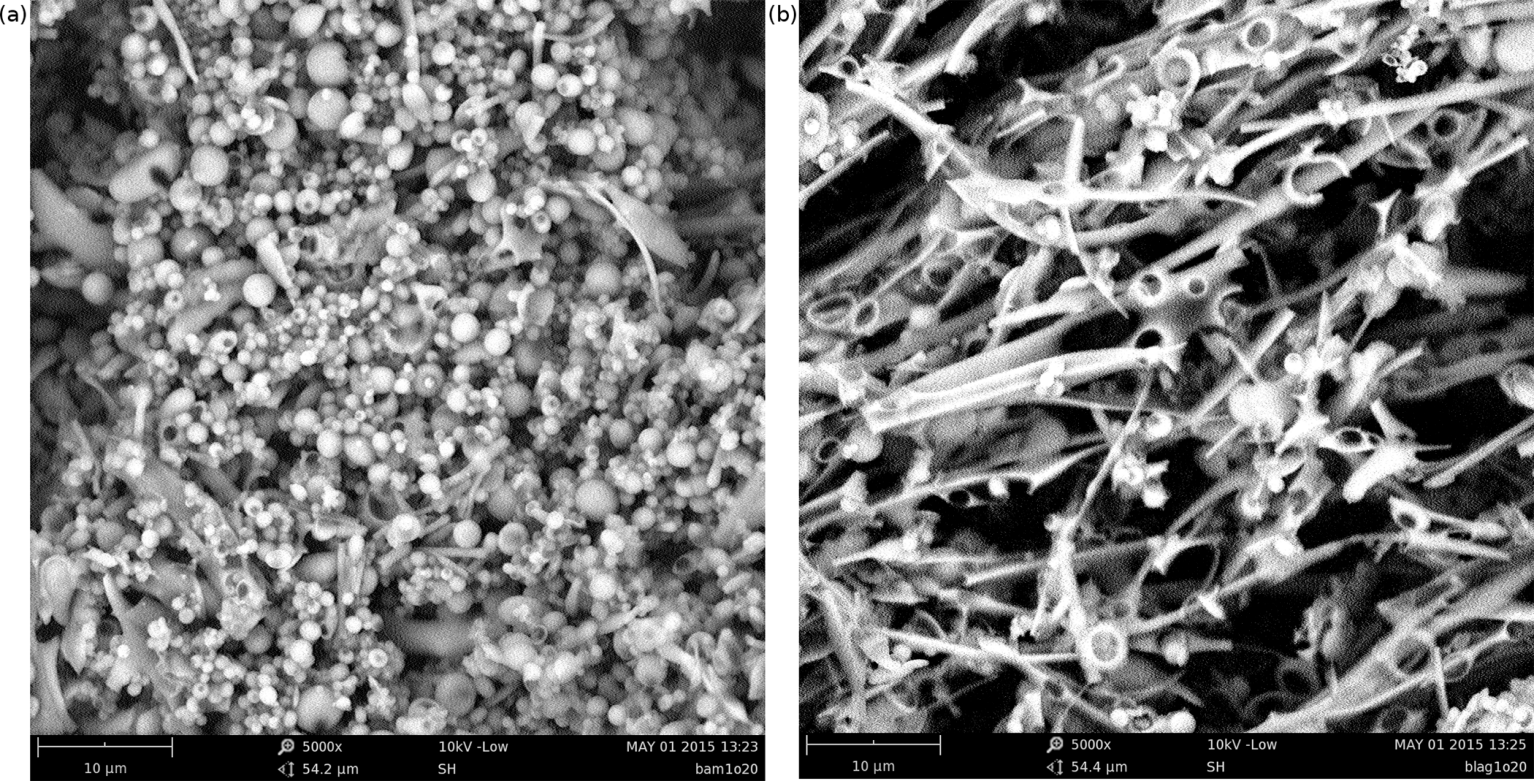
SEM of BAMLET and BLAGLET. (a) SEM of BAMLET-high powder having protein to lipid molecular ratios of 1:20, showing that this BAMLET species forms sphere-like aggregations of approximately 1–3.5 μm diameter. Our SAXS results demonstrate that at pH 12, these aggregations separate into monodispersed particles. (b) SEM of BLAGLET-high powder having protein to lipid molecular ratios of 1:20, showing that this BLAGLET species forms fibre-like aggregations of approximate width 0.5–1.5 μm and lengths of 25 μm and longer observed.

Scanning electron microscopy (SEM) of BAMLET-high powder (Fig 5A) shows that spheres of approximately 1–3.5 μm (10 000–35 000 Å) diameter predominate. SEM of BLAGLET-high powder (Fig 5B) shows that long, thin fibre-like structures of length 25 μm and longer and width 0.5–1.5 μm predominate. These features are in contrast to α-lactalbumin and β-lactoglobulin powder that show no such features when viewed by SEM (Figure L in S1 File).

### Circular dichroism shows that thermal denaturation of BLAGLET is gradual and reversible, whereas for apo-β-lactoglobulin it is gradual and irreversible

**Fig 6:**
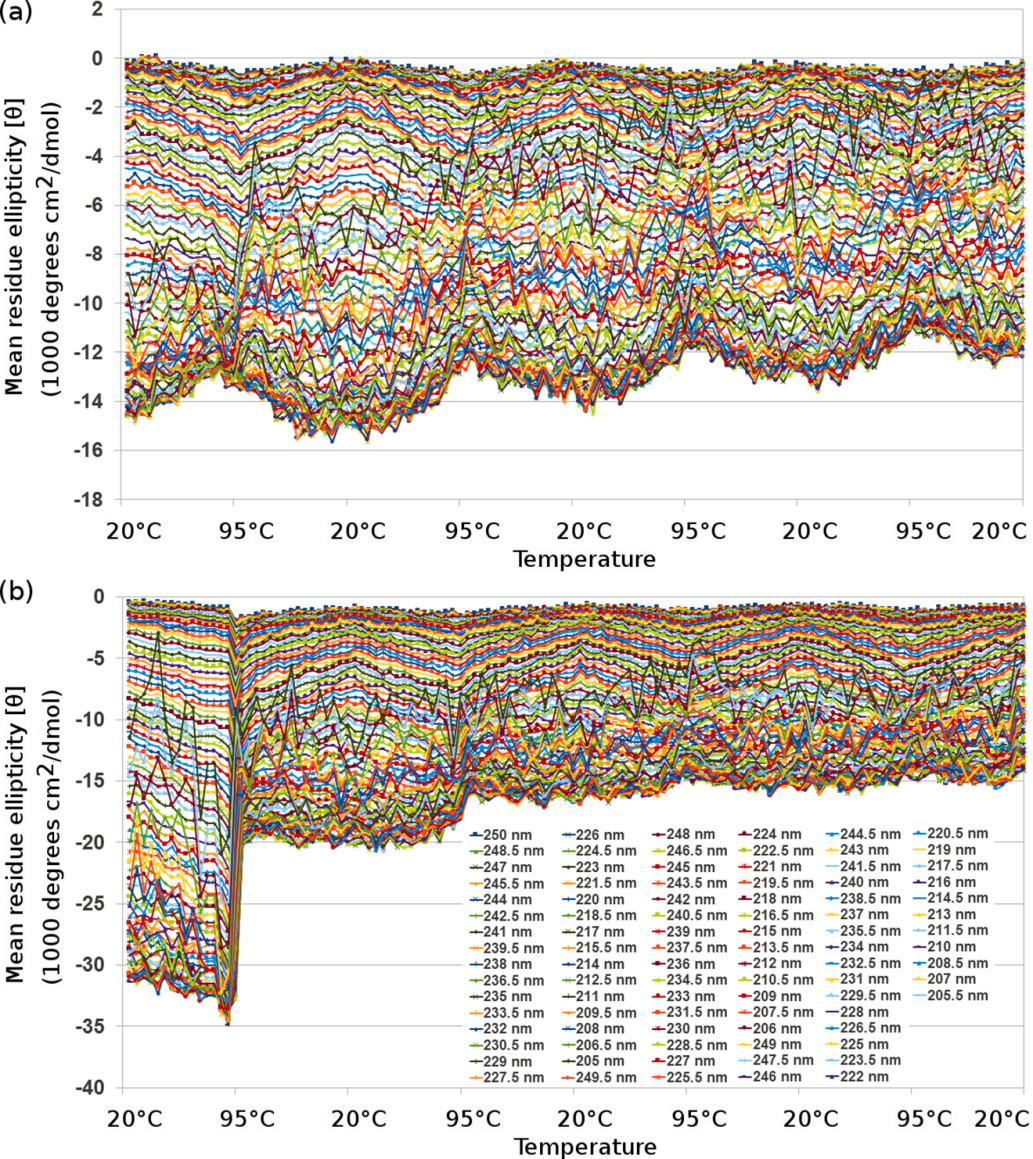
**Plot of CD wavelengths from 190 nm to 250 nm by 0.5 nm, changing temperature by 5°C approximately every 5 minutes** for (a) BLAGLET-high in pH 7, and (b) apo-β-lactoglobulin in pH 7. Similar to the preparation of BLAGLET, apo-β-lactoglobulin was prepared by heating β-lactoglobulin to 85°C for 1 hour then lyophilised. For circular dichroism experiments, temperature was initially 20°C then raised to 95°C and lowered to 20°C four times, with CD readings taken every 5°C change in temperature. At 20°C and 95°C, the samples were held at that temperature for more than 5 minutes.

As occurred for BAMLET-high and α-lactalbumin [69], circular dichroism of cycles of heating BLAGLET-high to 95°C and cooling back to 20°C reveal that there is no pronounced transition temperature at which the protein changes from mainly folded to mainly unfolded or vice versa (Fig 6A and Figures M and N in S1 File). The unfolding steps are small and numerous and give the impression of steady unfolding. One can imagine increasing amounts of oleic acid being able to associate with the protein as the protein increasingly unfolds. The thermal denaturation is a mostly reversible process for BLAGLET-high. Repeated temperature cycles for apo-β-lactoglobulin show that the thermal denaturation is gradual in appearance, however it is not reversible (Fig 6B and Figure O in S1 File). After heating to 95°C, the protein of apo-β-lactoglobulin remains denatured when cooled to 20°C. This characteristic may be due to the formation of disulphide bonds at the higher temperatures when the protein is unfolded, that remain bonded and impede the protein from folding again when the temperature is reduced to 20°C. This may be occurring for apo-β-lactoglobulin and not for apo-α-lactalbumin ([69] showed that thermal denaturation of apo-α-lactalbumin is gradual and reversible) due to β-lactoglobulin having an odd number of cysteines (5 cysteines) and thus the folded monomer cannot have all cysteines engaged in disulphide bonds within the monomer. α-Lactalbumin has an even number of cysteines (4 cysteines) and thus its folded, disulphide-bonded monomer does not have a free, highly-reactive cysteine. The BLAGLET and apo-β-lactoglobulin thermal denaturation results show that oleic acid in the BLAGLET complex inhibits the unfolding irreversibility observed for apo-β-lactoglobulin. If this irreversibility is due to unpaired disulphide bonds between cysteines of neighbouring β-lactoglobulin molecules, then oleic acid inhibits the formation of these neighbouring bonds.

## Discussion

In this study, we investigate the *in vitro* efficacy of BAMLET complexes in killing mesothelioma cells. Our cell viability results indicate that BAMLET may be a potential treatment option for mesothelioma. In most cases, chemotherapy-resistant mesothelioma cells and their parental cells that are not as chemotherapy-resistant showed similar sensitivity to BAMLET, indicating a potential for BAMLET to treat chemotherapy-resistant disease. We show that BAMLET is able to kill mesothelioma cells at doses for which non-cancer cells remain viable. BAMLET is cytotoxic to all 11 human mesothelioma cell lines tested, which represent the most common types of mesothelioma. BAMLET was as cytotoxic towards mesothelioma cells that had developed resistance to one of the main chemotherapies used to treat mesothelioma, cisplatin, as it was towards the parental non-resistant mesothelioma cell line. In a majority of experiments, BAMLET was as cytotoxic towards mesothelioma cells that had developed resistance to the other main chemotherapy used to treat mesothelioma, pemetrexed. BAMLET was also as cytotoxic towards mesothelioma cells that were receiving and surviving regular dosing with vinorelbine, another chemotherapeutic agent used to treat mesothelioma. These results indicate that BAMLET may be effective in killing cancer cells in tumours that have developed resistance to cisplatin, pemetrexed, and vinorelbine. We hypothesise that this is due to the BAMLET mechanism of action being different to the cytotoxicity mechanisms of cisplatin, pemetrexed, and vinorelbine. Mesothelioma cells that have developed resistance to the gemcitabine chemotherapy, however, were more resistant to BAMLET. This indicates that resistance to gemcitabine may confer resistance to BAMLET. Whilst BAMLET was as cytotoxic to chemotherapy-resistant cells as to chemotherapy-naive cells in most cases, the finding that it was not in all cases (ie, it was not the case for gemcitabine and sometimes for pemetrexed) demonstrates that BAMLET is targeting biological mechanisms and is not an indiscriminate poison. Our results add to the cancer cell types for which BAMLET is cytotoxic, extending the list to over 60 cancer cell types, now including mesotheliomas. We show that although mesothelioma cells are more sensitive to BAMLET in the absence of serum, BAMLET is nonetheless able to kill mesothelioma cells even when blood serum components are present. Although BAMLET is partially deactivated by the calcium and albumin in blood, we show that increasing the BAMLET dose by a factor of 10 makes it possible to achieve the same level of cytotoxic activity towards mesothelioma cells incubating in FBS-containing medium (containing albumin and calcium) as when these cancer cells are incubated in medium containing no FBS (containing calcium but no albumin). These results lead us to make the suggestion that BAMLET administered to mesothelioma patients by direct instillation into the lung pleural cavity, thus avoiding the intravenous route that would put BAMLET in contact with high concentrations of deactivating blood components, may result in BAMLET cytotoxicity to tumour cells. This approach warrants further investigation in *in vivo* models. Such treatment would require a high enough dose of BAMLET to overcome the BAMLET-deactivating effects of calcium and any albumin in the serous fluid in the pleural cavity. Given that BAMLET has been shown to act on the cell membrane and has not been shown to diffuse extensively through tissue, we envisage that repeated treatments would be necessary to continue tumour shrinkage by killing cancer cells at the edges of the tumour.

We demonstrated that primary non-cancer cells are more resistant to BAMLET than mesothelioma cancer cells. This window of resistance was small yet statistically significant. For non-cancer immortalised mesothelial cells, there was no BAMLET window of resistance compared to mesothelioma cells. For these non-cancer immortalised cell lines, there was no window of resistance for pemetrexed or cisplatin either, which are the current standard chemotherapies for treating mesothelioma [6]. That pemetrexed and cisplatin showed no *in vitro* therapeutic window and are the current standard of care appears as a paradox. There was previously no effective drug therapy option for mesothelioma, and cisplatin had already demonstrated anti-cancer activity for other cancers *in vitro*, in animal experiments, and in human patients [76–77], and so cisplatin treatment was trialled on mesothelioma patients [78–81]. This was carried out before subsequent cisplatin trials in animals [81–83]. When cisplatin was finally tested on mesothelioma cells *in vitro*, it was found to have no activity [84] or variable activity [81,85]. The rationale for trialling pemetrexed, a folic acid analog, to treat human mesothelioma patients was that it had shown *in vitro* anti-cancer activity for other cancer types, and that mesothelioma tumours had been shown to overexpress a folate receptor protein and other antifolates appeared to have activity in humans for treating mesothelioma [6,86–89]. Pemetrexed had also been tested on three patient-derived cell lines and found to be cytotoxic to two [84]. Although it has not been discussed in published studies, human non-cancer immortalised mesothelial cell line MeT5A has been shown in published studies to be just as sensitive to cisplatin as human mesothelioma cell lines [90–91], and rat non-cancer immortalised mesothelial cell line 4/4RM.4 had similar or increased sensitivity to cisplatin, pemetrexed, gemcitabine, vinorelbine, and paclitaxel as rat mothelioma cell lines [92]. We observed that three recently established slow growing non-cancer human fibroblast cell lines were more resistant to BAMLET than mesothelioma cells, whereas a fourth one established 4 years prior to the others by the same method had similar sensitivity to BAMLET as the mesothelioma cells. This may imply that the therapeutic effect of BAMLET is cell-cycle dependent, such that BAMLET has a more potent effect on highly proliferating cells. In this case, given the implication of the cell membrane in the BAMLET mechanism of action, the BAMLET cytotoxicity mechanism may involve an increased sensitivity to oleic acid induced membrane disruption during cell division. The effects of BAMLET on rapidly proliferating cells warrants further investigation. This observation also recalls reports by other researchers that recently established non-cancer primary cells are resistant to BAMLET and HAMLET while long-time established non-cancer immortalised cell lines are sensitive [12,14,35]. Such results suggest that over time, increased adaptation of non-cancer cells to *in vitro* culture may increase their sensitivity to BAMLET, and also suggests that such increased sensitivity may not o*ccur in vivo*. The BAMLET therapeutic window may be larger *in vivo* than what was found in these *in vitro* results as no toxic effects to healthy cells were observed in the bladders of humans, rats and mice after treatment with very high concentrations of HAMLET or BAMLET (25, 10, and 10 mg/ml respectively) [14–15,23], whereas experiments reported here at the lower dose we used (5 mg/ml) killed healthy fibroblast cells grown *in vitro*. Our BAMLET treatment window of resistance for human non-cancer primary fibroblasts compared to mesothelioma cells disappeared when serum was removed for the BAMLET incubation period. This protocol unfortunately would have removed growth factors in the medium generated and built up by the non-cancer fibroblasts. The loss of resistance indicates that the non-cancer primary cells were more sensitive than mesothelioma cells to the treatment protocol, ie. to the sudden removal of growth factors and/or albumin. This result suggests that BAMLET cell viability assays performed using the standard BAMLET protocol of removing FCS-containing medium for the BAMLET incubation period may misleadingly fail to find a window of resistance and this would be erroneously attributed to BAMLET toxicity towards non-cancer cells when it is in fact due to the sensitivity of the non-cancer cells to the treatment protocol.

Over 20 years of BAMLET research has resulted in many investigations into the BAMLET mechanism of anti-cancer activity and has thrown up many possible cellular targets, but as yet there is no full understanding. The BAMLET mechanism of anti-cancer activity is in some way or another related to the BAMLET structure releasing oleic acid to perturb cancer cell membranes (reviewed in [22]) and thus is not the same mechanism employed by currently used anti-cancer drugs. ATP synthase is a membrane protein involved in energy production previously shown to be a HAMLET target, with HAMLET treatment resulting in a dose-dependent reduction in levels of the ATP energy molecule [58]. Our qPCR results show a BAMLET dose-dependent reduction in gene expression of ATP synthase components and of ribosomal RNA. These results indicate that BAMLET treatment leads to a decrease in cancer cell energy production capacity and metabolic activities. Multiple lines of investigation by others indicate that the cellular target of BAMLET is the plasma membrane [30,43,59–65]. Our bioinformatics analysis of bacterial membrane proteins revealed ATP synthase as the only membrane protein to have a structural difference in HAMLET-sensitive bacteria compared to HAMLET-resistant bacteria, the difference being the lack of subunit I in sensitive bacteria. This finding that ATP synthase may be a HAMLET target in bacteria is consistent with the findings that HAMLET kills bacteria by triggering a dissipation of the proton gradient across the plasma membrane [26–29]. The ATP synthase complex is composed of the membrane-embedded F0 component and soluble catalytic F1 component, and our results that cancer cells survive regular BAMLET dosing by downregulating ATP synthase is consistent with the hypothesis of ATP synthase as a membrane target of BAMLET. Proton-pumping, energy-generating F0F1 ATP synthase has been identified as present on the plasma membrane of cancer cells [93–96] whereas in most types of healthy cells it is located in the mitochondria and not on the plasma membrane, which suggests that ATP synthase could be an effective potential target for anti-cancer therapy. The membrane-embedded c-ring of F0 ATP synthase has been demonstrated to be capable of forming a pore when in high calcium conditions [97]. The soluble F1 component of ATP synthase drastically changes position after treatment with HAMLET, from a punctate cell surface staining-pattern to a cytoplasmic pattern of aggregates, suggesting that the soluble F1 component has dissociated from the membrane F0 component in response to HAMLET [58]. The centre of the F0 ATP synthase c-ring is hydrophobic with the exception of an accessible threonine residue, is thought to likely contain a phospholipids plug, and the structural mechanism of how this centre could become a pore is a matter of debate [98]. The relationship of BAMLET and its possible compromising of cancer membranes via structural or regulatory effects on ATP synthase is an intriguing hypothesis worthy of further investigation. Our cell death assay results suggest an additional potential membrane target of BAMLET–the SLC28 and SLC29 concentrative and equilibrative nucleoside transporters that transport the chemotherapy drug gemcitabine into the cell [99–100]. We found that mesothelioma cells that had developed resistance to gemcitabine had also developed resistance to BAMLET. A plausible membrane-associated hypothesis for this relationship is that the membrane-embedded gemcitabine/nucleoside transporters are a target of BAMLET and their downregulation leads to decreased sensitivity to BAMLET in addition to decreased uptake of and decreased sensitivity to gemcitabine. Further investigations would be to see if removing these nucleoside transporters removes sensitivity to BAMLET, and a possible confounding issue for such investigations would be that some inhibitors of membrane channels and transporters also deactivate BAMLET by causing oleic acid to precipitate out of the complex (eg. barium chloride and amiloride [22]). Oleic acid is likely to have multiple membrane targets due to its structure. Oleic acid is a simple lipid having the same length as the width of one layer of the lipid bilayer and thus has high affinity for the lipid bilayer. Oleic acid's one unsaturated cis double-bond is responsible for a kink in oleic acid structure, a characteristic that promotes softening, disordering and disruption of the lipid bilayer. Although it has been shown by multiple research groups that the oleic acid component of BAMLET is the active component [14,18], our cell death assay results show that high doses of the α-lactalbumin protein component also have toxic effects towards cancer cells, and this cytotoxicity is not due to the protein being unfolded as the protein in our α-lactalbumin-only assays was not unfolded. These results echo previous findings that pure protein causes a 20% reduction in the growth rate of cells as measured by thymidine incorporation [12]. However, significantly higher doses of α-lactalbumin were required for cytotoxicity when compared with BAMLET, and thus α-lactalbumin can be used as a control in BAMLET viability assays at the lower concentrations required for BAMLET cytotoxicity.

Our findings that BAMLET has similar cytotoxicity towards cisplatin-resistant and pemetrexed-resistant cancer cells as towards chemotherapy-sensitive cells provides a further indication that the BAMLET mechanism of anti-cancer activity is different to anti-cancer mechanisms of cisplatin and pemetrexed. These results echo previous findings that HAMLET is just as cytotoxic towards apoptosis-resistant cells (p53 mutant and bcl-2 overexpressing) as for their respective apoptosis-sensitive parental cell lines [21], and BAMLET is just as cytotoxic towards TRAIL-resistant cell lines as for TRAIL-sensitive cell lines [15]. This is of particular interest for the field of mesothelioma because mesothelioma is a cancer that is resistant to the available chemotherapies. We found that genes involved in energy production and metabolism (ie. ATP synthase and ribosomal RNA) are downregulated in mesothelioma cells in response to regular BAMLET dosing, indicating that mesothelioma cells need to slow down their metabolism in order to survive regular BAMLET dosing. This suggests that the novel BAMLET mechanism of activity is associated with cellular metabolism, in addition to the now substantial body of evidence that the BAMLET mechanism involves the cell membrane.

Our SAXS structural results reveal how BAMLET and BLAGLET complexes hold oleic acid in a solubilised, activated state, and give insights into how this structure is able to incorporate increasing amounts of oleic acid resulting in the increased cytotoxicity to mesothelioma cells that we demonstrated in our cell viability assays. In brief, the more oleic acid incorporated into BAMLET complexes, the more the protein structure unfolds to accommodate the increased oleic acid volume in the core, without changing the nature of the binding between protein and lipid. In this structural system, the oleic acid appears to be not bound in specific protein binding sites and not bound tightly to the protein. This continuum of partially unfolded states was hinted at in our previous CD work on BAMLET [69] and remains consistent with the work on BLAGLET here. The results presented here now convincingly show, by SAXS, the structural continuum of partial unfolding for both BAMLET and BLAGLET protein. Our SAXS-derived structural models and parameters show that the BAMLET and BLAGLET protein structures grow in size as increasing amounts of oleic acid are added to the complex. Our recent small angle neutron scattering studies show that the oleic acid is located in the core of this structure[68]. The protein structures grow in size incorporating the increased oleic acid volume in the core in the same conformation of non-specific binding sites. It is the increased amount of oleic acid present in the solubilised, activated state that is mainly responsible for the increased cytotoxicity of the higher oleic acid content BAMLETs and BLAGLETs compared to the lower oleic acid content complexes. This state is referred to as a “solubilised” state because the oleic acid component remains inside its parent protein in an aggregated form dissolved in solution instead of the oleic acid forming one larger separate oil slick phase. This solubilised state of oleic acid, loosely bound to the protein, is referred to as “activated” because although the complex remains intact in water solutions, the oleic acid is readily dissociated from the protein and therefore able to enter the lipid membrane when the complex comes into contact with cancer cell membranes. Oleic acid is an oil and as oil and water are not comfortable bedfellows we postulate that the protein component of BAMLET acts as a surfactant, inhibiting the oleic acid from forming an unreactive micelle, keeping it emulsified in an aqueous environment, most probably via attraction of the hydrophobic fatty acid tail to the hydrophobic interior surfaces of the partially unfolded soluble protein. Our CD results for repeated cycles of thermal denaturing of BAMLET [69] and BLAGLET (in the present work) demonstrate that the BAMLET-like structure exhibits flexibility as well as instability, unfolding as the temperature increases and refolding around the oleic acid as the temperature decreases. We postulate that this flexibility and instability are central to the BAMLET “cargo-offloading” mechanism. This mechanism means the protein can keep oleic acid encapsulated when the complex is in an aqueous environment, and then fail to hold on to the oleic acid when the complex comes into contact with hydrophobic cell membranes, much like a scheduled cargo unload.

While both BAMLET and BLAGLET were cytotoxic towards mesothelioma cells, our studies reveal structural differences between BAMLET and BLAGLET. Our SAXS results show that BLAGLET, prepared from bovine β-lactoglobulin protein, remains aggregated in conditions where BAMLET, prepared from bovine α-lactalbumin, is no longer aggregated. BLAGLET was more resistant to its aggregated form being deaggregated, and we showed that this is due to intermolecular disulphide bonds. SAXS-derived parameters and models showed that as oleic acid content is increased, the molecular dimensions of the ellipsoidal BLAGLET steadily increases whereas that of BAMLET actually decreases and BAMLET becomes more spherical, hinting at differences in the structural mechanisms for binding increasing amounts of oleic acid. SEM images show a striking difference in the aggregated forms of these two BAMLET-like species. BAMLET aggregations are spherical, while BLAGLET aggregations are long fibre-like structures, as a result of their intermolecular disulphide bonds. These structural differences presented in the current work hint at possibilities for creating BAMLET-like compounds having tuneable structural characteristics that result in tuneable biological characteristics with regards to stability and release of the active oleic acid component to kill cancer cells.

## Methods

### Preparation of BAMLET and BLAGLET complexes

BAMLET and BLAGLET complexes were prepared according to the heating protocol [37]. Lyophilised bovine α-lactalbumin protein (99% purity, calcium-depleted from Sigma-Aldrich for all BAMLET experiments other than some cell death assays, or 90% purity α-lactalbumin from Davisco Foods International Inc., Le Sueur, MN, USA, for some BAMLET cell death assays) or bovine β-lactoglobulin protein (Sigma-Aldrich, Sydney, Australia) was dissolved in 150 mM NaCl, 1 mM EDTA, at pH 8.3, at a concentration of 1 to 50 mg/ml. BAMLET complexes having protein:oleic acid ratios 1:5.625, 1:6.25, 1:7.5, 1:8.75, 1:12.5, 1:15, and 1:17.5 were prepared by mixing appropriate quantities of BAMLET complexes having ratios 1:5, 1:10, and 1:20 in 45 mM glycine, 55 mM NaOH, pH 12. Protein concentrations were determined by absorption of light at wavelength 280 nm, using extinction co-efficients 28 460 for BAMLET and α-lactalbumin and 17 600 for BLAGLET and β-lactoglobulin, on a Thermo Scientific NanoDrop 2000c UV-Vis Spectrophotometer. Oleic acid (4 mL) was dissolved in ethanol (50 mL). Protein in solution was heated under continual stirring to 45°C for α-lactalbumin and to 60°C for β-lactoglobulin and incubated for 15 min for α-lactalbumin and for 60 min for β-lactoglobulin (β-lactoglobulin required incubation for a longer time at higher temperature than α-lactalbumin in order to effectuate unfolding of the protein). Oleic acid in ethanol was vortexed for 1 min then added to stirring solution by descending pipette tip into solution and slowly releasing oleic acid in a steady stream. Stirring at the same temperature continued for 10 min, and then stirring ceased and the solution allowed to cool to room temperature. The solution was dialysed (SnakeSkin Dialysis Tubing, Thermo Scientific, membrane cutoff 3.5 kDa) for 3 days in Elga filtered water (Purelab Option-Q) at 4°C with a change of water every 24 hrs. The solution was flash-frozen in aliquots of 10 ml in 50 ml falcon tubes descended into liquid nitrogen, freeze dried (ALPHA 1-2-LDplus, Martin Christ GmbH, Germany) in the dryer’s cryogenic chamber at -41°C and 0.37 mbar for 5 days or until all water was removed, and the lyophilised BAMLET or BLAGLET powder was stored at 4°C. Aliquots are described using content ratios 1:N where N is moles of oleic acid per mole of protein, as prepared. No residue protein or oleic acid was observed on surfaces. BAMLET-low, BAMLET-medium, and BAMLET-high refer to BAMLET complexes having N = 1, 5 and 20 respectively as per [69]. BLAGLET-low, BLAGLET-medium, and BLAGLET-high refer to BLAGLET complexes having N = 1, 5 and 20 respectively. BAMLET-medium is the BAMLET species used in experiments except where otherwise specified.

### Cell culture and cell death assays

The human malignant pleural mesothelioma cell lines H28, H226, H2052, H2452, MSTO, and the immortalised human mesothelial cell line MeT5A [101] were purchased from the American Type Culture Collection (ATCC, Manassas, VA, USA). REN human mesothelioma cells [102] were provided by Laura Moro (University of Piemonte Orientale A. Avogadro, Novara, Italy). The primary human mesothelioma cell line MM05 was generated at the University of Queensland Thoracic Research Centre (The Prince Charles Hospital, Brisbane, Australia) [103]. The human mesothelioma cell lines VMC23 (and VMC33 taken from the same patient after chemotherapy) [104] and VMC20 [105] were described previously and human mesothelioma cell line VMC40 established by the same group were kindly provided by Walter Berger (Institute of Cancer Research and Walter Klepetko, Medical University of Vienna, Austria). The mesothelioma types represented are epithelioid (REN, H28, H226, H2452, H2052, VMC20, VMC23 and VMC33) and biphasic (MM05, MSTO and VMC40). All cell lines were cultured in RPMI 1640 with 10% FBS, 1% penicillin-streptomycin (PS) and maintained at 5% CO_2_, 37°C and 95% humidity. The rat mesothelioma IL45 cell line [106] was a kind gift from Associate/Professor Emanuela Felley-Bosco (Zurich University). The chemotherapy-resistant IL45 rat mesothelioma cell lines were described previously [92], and were maintained for resistance with the following high or low doses respectively once a week: 2.5 μM and 1.8 μM cisplatin for IL45-CisR, 1.05 μM or 105 nM pemetrexed for IL45-PemR, 70 nM and 15 nM gemcitabine for IL45-GemR, and 105 nM and 35 nM vinorelbine for IL45-VLBR. The immortalised mesothelial 4/4RM.4 cell line from female Fischer 344 rats [107] was purchased from ATCC (CCL-216). Primary non-cancer human dermal fibroblasts (Humofib, HOFF, HOFMO1, and HOFMO2) were isolated from biopsies of healthy patients undergoing abdomen reduction surgery at Concord Hospital, with hospital ethics approval (CH62/6/2006-026) and have been used in a number of studies (see [108] and references therein).They were chosen for these experiments because they have mesothelial-like visual characteristics (Figure B in S1 File). Alamar Blue cell death assays were carried out for human cells as follows. Cells were plated in 96-well culture plates at 3 000 cells in 100 μl medium per well for cancer and immortalised mesothelial cell lines and at 5 000 cells in 100 μl per well for non-cancer primary cells. After 24 hr, non-cancer and cancer cells reached similar confluence and cells were treated. For experiments having FBS present during dosing, 20 μl of BAMLET or chemotherapy treatment in RPMI was added and incubation continued for 72 hr. For experiments having no FBS present during dosing, medium was removed and 100 μl of BAMLET, BLAGLET, or α-lactalbumin treatment in medium without FBS was added and incubated for 4.5 hr after which 100 μl medium containing 20% FBS and 2% Penicillin-Streptomycin was added to give a final FBS concentration of 10% and incubation continued for 24 hr. Alamar Blue, 20 μl (50 mL PBS containing also Sigma reagents 0.075 g Resazurin, 0.0125 g Methylene Blue, 0.1655 g Potassium hexacyanoferrate (III), 0.211 g Potassium hexacyanoferrate (II) trihydrate, filter-sterilised, and stored at 4°C in the dark), was added and incubated for 2 to 4 hr at 37°C. Fluorescence intensity was measured at 590–10 nm with 544 nm excitation, using a FLUOstar Optima (BMG LabTech, Ortenberg, Germany). Fluorescence intensity was presented as a percentage of intensity of control cells. Experiments involving human cell lines were performed 3 times with 3 replicates each time, except for experiments involving slow-growing non-cancer primary fibroblasts that were performed 4 times with 2 or 3 replicates. All media and FBS were from Life Technologies (Carlsbad, CA, USA). Where indicated, cells were treated with pemetrexed, gemcitabine, or vinorelbine (Eli Lilly, Sydney, Australia), or cisplatin (McFarlane Medical & Scientific, Sydney, Australia).

### Calculations of TC_50_ and statistical significance of cell assays

Viability percentage was calculated from intensity measurements as the ratio of intensity treatment to intensity control where the control is the treatment concentration of zero. Cell death assays involving human non-cancer fibroblasts that appeared to have a biphasic response to BAMLET treatment were analysed by statistical modelling carried out in R [109] to determine models, TC_50_ values, and statistical significance. Cell death assays comparing viability of chemotherapy-resistant and parental rat mesothelioma cells in response to chemotherapy or BAMLET treatment were analysed by statistical modelling in R to determine statistical significance. A sigmoid function [110] was used to model treatment responses for cell lines in the cell death assays, except in the case of Pemetrexed treatment of rat mesothelioma cells for which a linear function was used. The sigmoid function used was:

$$viability\;fraction=A+(B-A)*\;\left(\frac{1}{\left(1+exp(\frac{\left(xmid-x\right)}{scale})\right)}\right)$$ (1)

where *A* is the left asymptote (viability at treatment concentration of 0, *B* is the right asymptote (viability at highest treatment concentration), *xmid* the transition point (TC_50_), *scale* is an x-axis scale parameter impacting slope of the transition, and *x* is log_10_ of the BAMLET concentration (thus rendering the curve symmetrical and suitable for modelling using log-likelihood).

Biphasic treatment responses were modelled using a biphasic version of the sigmoid function:

$$viability=A+(B-A)*\;\left(\frac{sensitive}{\left(1+exp(\frac{\left(xmid-x\right)}{scale})\right)}+\frac{\left(1-sensitive\right)}{\left(1+exp(\frac{\left(xmid+xshift-x\right)}{scale})\right)}\right)$$ (2)

where *A* is the left asymptote, *B* is the right asymptote, *xmid* is the transition point for the BAMLET-sensitive cells (TC_50_ of BAMLET-sensitive cells), *xshift* is the shift in transition point for BAMLET-resistant cells (*xmid* + *xshift* = TC_50_ of BAMLET-resistant cells), *sensitive* is the sensitive cell fraction, *scale* is an x-axis scale parameter impacting slope of the transitions, and *x* is log_10_ of the BAMLET concentration.

Models for a response of a cell line to treatment fit *A*, *B*, *xmid*, *xshift*, and *scale* for all experiments for a given cell line, and fit *sensitive* per experiment per cell line. The maximum log likelihood method was used to determine the best fitting parameters for a given model, using the optimx R package, including determination of TC_50_. The likelihood ratio test was used to compare 2 competing models where the models are nested, ie. one model is obtained from the other model by removing parameters, the models referred to as “complex” and “simpler” respectively. The chi-squared statistic was used to determine whether the improvement of fit for the more complex model over the simpler model is statistically significant, using the following formulae for chi-squared value and degrees of freedom:

$$X^{2}{}_{difference\_in\_models}=X^{2}{}_{complex\_model}–X^{2}{}_{simpler\_model}$$ (3)

$$df_{difference\_in\_models}=df_{complex\_model}–df_{simpler\_model}$$ (4)

This method was first used to demonstrate that the response to BAMLET of the non-cancer fibroblast cells is biphasic. This method was then used to determine whether the shift in TC_50_ of BAMLET-resistant non-cancer cells compared to the TC_50_ of cancer cells is statistically significant. This method was used to determine whether viability responses to chemotherapy or BAMLET treatment were statistically significantly higher for chemotherapy-resistant rat mesothelioma cells compared to chemotherapy-sensitive parental cells. TC_50_ values for all other cell death assay experiments (BAMLET and BLAGLET assays of human mesothelioma cell lines and BAMLET assays of rat mesothelioma cell lines) were calculated by linear regression in LibreOffice Calc. All R code for TC_50_ modelling using sigmoidal or linear models are in the code of S1 Dataset. Cell assay data used in R modelling and calculations are in S1 Dataset.

### Reverse transcription and quantitative real-time PCR (RT-qPCR)

RT-qPCR was carried out following a protocol based on [111]. Briefly, total RNA was extracted from cell lines using Trizol reagent (Life Technologies) following the manufacturer’s protocol. Reverse transcription reactions were performed using 200 ng of total RNA with MMLV first strand cDNA kit (Promega, Madison, WI) following the manufacturer’s protocol. Gene expression was determined by quantitative real-time PCR using the Kapa SYBR ® Fast qPCR Master Mix (Sigma-Aldrich) and the Vii7 QPCR System (Thermo Fisher Scientific). Probe Design software (Roche Diagnostic) was used for designing PCR primers (Table E in S1 File). For each gene in a cell line, qPCR cycle threshold was collected for 3 replicates. Expression levels of mRNA were determined using the 2^−ΔΔCq^ method [112–113] with normalisation to the reference gene. Statistical significances were determined by a randomisation test consisting of 10,000 permutations [113–114]. Data and R code are in S3 Dataset.

### Small angle X-ray scattering and analysis

Lyophilised BAMLET or BLAGLET was dissolved in 45 mM glycine, 55 mM NaOH, pH 12. For further experiments, lyophilised BLAGLET was dissolved in 45 mM glycine, 55 mM NaOH, pH 12, 2% β-mercaptoethanol (to deaggregate and produce monodispersed BLAGLET). Samples were dialysed in the buffer solution for at least 24 h at 4°C to provide a buffer blank. Samples were successively diluted by 50% to create a concentration series. Scattering data were collected as I(q) versus q, where the scattering vector q = 4πsinθ/λ, 2θ is the scattering angle, and λ is the X-ray wavelength. SAXS data for BAMLET and BLAGLET β-mercaptoethanol samples were collected on the Australian Synchrotron SAXS/WAXS beamline [115], wavelength 1.127 Å, sample exposure time of approximately 5 s on a Pilatus 1M photon counting detector (Dectris) placed 1.48 m from the sample capillary, and measurements were binned in 5 s units. Samples were fed to the beam by capillary from a 96-well plate, 100 μl per well, kept at 4°C for BAMLET and 10°C for BLAGLET by a Huber Ministat 230 Circulation thermostat. Data reduction was performed with the beamline specific software package “scatterbrain” (Australian Synchrotron "http://www.synchrotron.org.au/aussyncbeamlines/saxswaxs/software-saxswaxs/") producing data on absolute scale. SAXS data for BLAGLET samples without β-mercaptoethanol were collected on a Bruker NanoStar II instrument (Karlsruhe, Germany), with a rotating anode copper Kα source (wavelength 1.541 Å), Montel mirrors, three pinhole collimation, a Vantec 2000 2D detector with 68 μm pixel size, and q range 0.01–0.42 Å^-1^. Samples of 40 μL were held in a capillary at 4°C. The instrument's software was used to integrate the detected intensity to produce the 1D scattering function I(q). Calibration to place pH 12 data on an absolute scale was performed using water [116]. For both BAMLET and BLAGLET SAXS data, the ATSAS suite of SAXS analysis programs [117] was used for SAXS analysis of the buffer blank subtracted data. SAXS of BAMLET and BLAGLET β-mercaptoethanol samples were analysed as monodispersed particles for which radius of gyration (R_g_) and molecular shape envelope can be determined. The R_g_ of the scattering complex and the scattering intensity extrapolated to zero q, I(0), were determined by two methods. One method was the Guinier approximation [118] (Guinier 1938) using the ATSAS program Primus. The other method was the numerical method of Glatter [74–75] using the ATSAS program GNOM. The Guinier analysis obtains R_g_ and I(0) from the slope and y-intercept, respectively, of the linear fit line ln(I(q)) versus q^-1^ using a subset of the low q SAXS data such that q x R_g_ < 1.30 [118]. The lowest q point used to derive this linear fit line was also used as the lowest q point for the numerical method and Fourier transform of Glatter. The probability distribution function, P(r), was derived by the indirect Fourier transform method [74–75] using the ATSAS program GNOM. Twenty 3D models representing the scattering particles were derived from the P(r) using the ATSAS DAMMIN program [119]. Simulated scattering from models was performed by DAMMIN and FoXS Server (Fast SAXS Profile Computation with Debye Formula) [119–121]. SAXS data are in S2 Dataset.

### Bioinformatics analysis

Gene sequences were obtained from NCBI Nucleotide (https://www.ncbi.nlm.nih.gov/nuccore) and Protein (https://www.ncbi.nlm.nih.gov/protein) databases. Multiple sequence alignments were carried out using Clustal Omega (1.2.3) (http://www.ebi.ac.uk/Tools/msa/clustalo/) using default parameters [122–124]. Functional analysis of protein sequences was performed using an Interproscan search for each sequence [70]. Gene searches were carried out using NIH Standard Protein BLAST (BLASTP 2.6.0+) (https://blast.ncbi.nlm.nih.gov/Blast.cgi) using default parameters [125–126]. The NCBI Nucleotide identifiers of genome sequences from which ATP synthase genes were obtained are NC_008533.1 (*S*. *pneumoniae D39*), CP014326.1 (Streptococcus mitis strain SVGS_061), NC_002737.2 (*Streptococcus pyogeneM1 GAS*), CP009685.1 (*Escherichia coli str*. *K-12 substr*. *MG1655*), BX571856.1 (*Staphylococcus aureus subsp*. *aureus strain MRSA252*), AE015929.1 (*Staphylococcus epidermidis ATCC 12228*), NZ_FO834906.1 (*Klebsiella pneumoniae str*. *Kp52*.*145*), CP007224.1 (*Pseudomonas aeruginosa PA96*), and CP009756.1 (*Enterobacter cloacae strain GGT036*). BLASTP search organisms were *Streptococcus pneumoniae (taxid*:*1313)*, *Streptococcus mitis (taxid*:*28037)*, *Streptococcus pyogenes (taxid*:*1314)*, and *Homo sapiens (taxid*:*9606)*.

### Scanning electron microscopy

Scanning electron microscopy (SEM) of α-lactalbumin, β-lactoglobulin, and of high oleic acid content BAMLET and BLAGLET in powder form was performed with a Phenom ProX Desktop SEM, having a Cerium Hexaboride filament (CeB6) source. A beam acceleration voltage of 10 kV or 5 kV was used, as indicated. The beam current was set between 0 and 8.1 nA as per the capabilities of this instrument. The vacuum level in the Phenom sample chamber is variable and the instrument's typical values of 0.1–0.3 mbar were used. A turbo molecular pump is used inside unit to maintain the source vacuum and a dry diaphragm pump is used externally. Magnification from 255× to 20,000×, typically 5,000×, was used as indicated. The instrument's detector is a Backscattered Electron (BSE) Detector consisting of four segments to collect the backscattered electrons. Samples were mounted on an aluminium pin stub, attached by a double sided Carbon pad sticker.

### Circular dichroism

Lyophilised β-lactoglobulin or BLAGLET was dissolved in 20 mM Tris-HCl, 2 mM EDTA, pH 7 at a concentration of 0.2 mg/mL. Protein (250 μL) in solution, or solution alone, was placed in a 1 mm pathlength quartz cuvette. Circular dichroism (CD) was performed using a Jasco (Tokyo, Japan) J-815 CD Spectropolarimeter fitted with a Peltier temperature controller. CD was performed for the far ultraviolet (UV) wavelengths 190–250 nm, in steps of 0.5 nm. The temperature of the samples was increased from 20 to 95°C in 5°C steps, with readings taken after 5 min incubation at each step. The same procedure was used reducing the temperature back to 20°C, and the cycle was repeated four times. Readings, with scanning speed 100 nm/min, scanning bandwidth 1 nm, were made in quadruplicate and averaged. Analysis of secondary structure content was performed for the buffer subtracted CD data using the Yang method [127].

## Supporting information

S1 File Supporting information file. (DOC)

S1 Dataset Data and code files for human and rat cell viability assays. (TGZ)

S2 Dataset Data files for BAMLET and BLAGLET SAXS experiments and SAXS-derived models. (TGZ)

S3 Dataset Data and code files for qPCR experiments. (GZ)

## Abbreviations

BAMLET: bovine alpha-lactalbumin made lethal to tumours
BLAGLET: beta-lactoglobulin made lethal to tumours
CD: circular dichroism
PCR: polymerase chain reaction
RT-qPCR: reverse transcription and quantitative real-time PCR
SAXS: small angle X-ray scattering
