# Supplementary material for: BAMLET kills chemotherapy-resistant mesothelioma cells, holding oleic acid in an activated cytotoxic state
Source: PLoS One. 2018 Aug 29;13(8):e0203003. doi: 10.1371/journal.pone.0203003 (PMC6114908; doi:10.1371/journal.pone.0203003)
Supplement: S1 File — (DOC) [file pone.0203003.s001.doc]

### Supplementary Information

*Supplementary Table A. Toxic concentration (TC_50_) at which 50% of human cells remain viable after in vitro BAMLET, BLAGLET or chemotherapy treatment. The treatment of zero concentration was used as control for calculating % viability. Experiments were carried out 3 times. TC_50_ values for the BAMLET-medium 3 days dosing experiments were calculated from statistical modelling. All other TC_50_ values shown are for a representative experiment of three replicates and were calculated from linear regression. Asterisk (*) denotes TC_50_ value that was extrapolated from experimental data when TC_50_ value did not fall within the experiment's range.*

| **Treatment** | | |
| --- | --- | --- |
|  | **Cell line** | **TC_50_** |
| BAMLET-medium, 3 days with FBS during BAMLET dosing (plotted Figure 1c) | | |
|  | MM05 | 0.681±0.100 mg/ml |
|  | H226 | 0.662±0.108 mg/ml |
|  | VMC23 | 0.600±0.112 mg/ml |
|  | VMC40 | 0.773±0.013 mg/ml |
|  | Met5A | 0.666±0.112 mg/ml |
|  | Humofib | 0.688±0.131 mg/ml |
|  | HOFM01 – BAMLET-resistant fraction  (51.3% of cells) | 1.786±0.712 mg/ml |
|  | HOFM01 – BAMLET-sensitive fraction | 0.722±0.229 mg/ml |
|  | HOFM02 – BAMLET-resistant fraction  (42.8% of cells) | 1.741±0.491 mg/ml |
|  | HOFM02 – BAMLET-sensitive fraction | 0.700±0.202 mg/ml |
|  | HOFF – BAMLET-resistant fraction (70.6% of cells) | 2.372±0.310 mg/ml |
|  | HOFF – BAMLET-sensitive fraction | 0.882±0.111 mg/ml |
| BAMLET-medium, 4.5 hours without FBS, then 3 days with FBS  (for comparison with the experiments of BAMLET-medium, 3 days with FBS) | | |
|  | MM05 | 0.048±0.024 mg/ml |
|  | H226 | 0.115±0.008 mg/ml |
|  | VMC23 | 0.031±0.001 mg/ml |
|  | HOFM01 | 0.132±0.001 mg/ml |
|  | HOFM02 | 0.106±0.010 mg/ml |
| α-Lactalbumin, 4.5 hours without FBS, then 1 day with FBS (plotted Figure 1a) | | |
|  | MM05 | TC_50_ not reached |
|  | MSTO | 58.188±5.085 mg/ml * |
|  | REN | 31.594±1.469 mg/ml |
|  | H28 | TC_50_ not reached |
|  | H226 | 16.336±4.603 mg/ml |
|  | H2452 | 38.344±8.416 mg/ml |
|  | H2052 | 13.108±2.902 mg/ml |
|  | VMC20 | 42.412±3.581 mg/ml * |
|  | VMC23 | 94.194±45.010 mg/ml * |
|  | VMC33 | 22.220±1.515 mg/ml |
|  | VMC40 | 25.570±1.972 mg/ml |
|  | MeT5A | 11.191±0.732 mg/ml |
| BAMLET-low, 4.5 hours without FBS, then 1 day with FBS (plotted Figure 1a) | | |
|  | MM05 | 3.949±0.302 mg/ml |
|  | MSTO | 0.959±0.157 mg/ml |
|  | REN | 2.066±0.058 mg/ml |
|  | H28 | 3.023±0.347 mg/ml |
|  | H226 | 2.428±0.749 mg/ml |
|  | H2452 | 2.255±0.296 mg/ml |
|  | H2052 | 3.240±0.392 mg/ml |
|  | VMC20 | 4.922±0.561 mg/ml |
|  | VMC23 | 2.879±0.610 mg/ml |
|  | VMC33 | 0.917±0.157 mg/ml |
|  | VMC40 | 1.884±0.088 mg/ml |
|  | MeT5A | 2.127±0.311 mg/ml |
|  | Humofib | 1.161±0.292 mg/ml |
| BAMLET-medium, 4.5 hours without FBS, then 1 day with FBS (plotted Figure 1a) | | |
|  | MM05 | 0.627±0.042 mg/ml |
|  | MSTO | 0.326±0.009 mg/ml |
|  | REN | 0.756±0.122 mg/ml |
|  | H28 | 0.582±0.014 mg/ml |
|  | H226 | 0.615±0.019 mg/ml |
|  | H2452 | 0.358±0.044 mg/ml |
|  | H2052 | 0.516±0.059 mg/ml |
|  | VMC20 | 0.599±0.009 mg/ml |
|  | VMC23 | 0.434±0.030 mg/ml |
|  | VMC33 | 0.274±0.009 mg/ml |
|  | VMC40 | 0.335±0.012 mg/ml |
|  | MeT5A | 0.296±0.043 mg/ml |
|  | Humofib | 0.315±0.005 mg/ml |
| BAMLET-high, 4.5 hours without FBS, then 1 day with FBS (plotted Figure 1a) | | |
|  | MM05 | 0.245±0.026 mg/ml |
|  | MSTO | 0.611±0.117 mg/ml |
|  | REN | 0.511±0.141 mg/ml |
|  | H28 | 0.267±0.007 mg/ml |
|  | H226 | 0.288±0.022 mg/ml |
|  | H2452 | 0.108±0.010 mg/ml |
|  | H2052 | 0.466±0.047 mg/ml |
|  | VMC33 | 0.071±0.004 mg/ml |
|  | VMC40 | 0.145±0.006 mg/ml |
|  | MeT5A | 0.136±0.027 mg/ml |
|  | Humofib | 0.054±0.001mg/ml |
| BLAGLET-low, 4.5 hours without FBS, then 1 day with FBS (plotted Figure 1b) | | |
|  | MM05 | 5.482±0.348 mg/ml * |
|  | MSTO | 2.537±0.199 mg/ml |
|  | REN | 2.794±0.031 mg/ml |
|  | H28 | TC_50_ not reached |
|  | H226 | 3.345±0.354 mg/ml |
|  | H2452 | 4.069±0.417 mg/ml * |
|  | H2052 | 2.938±0.127 mg/ml |
|  | VMC20 | 1.636±0.144 mg/ml |
|  | VMC23 | TC_50_ not reached |
|  | VMC33 | 3.378±0.051 mg/ml |
|  | VMC40 | 2.843±0.015 mg/ml |
|  | MeT5A | 2.671±0.213 mg/ml |
| BLAGLET-medium, 4.5 hours without FBS, then 1 day with FBS (plotted Figure 1b) | | |
|  | MM05 | 0.884±0.097 mg/ml |
|  | MSTO | 0.429±0.036 mg/ml |
|  | REN | 0.568±0.037 mg/ml |
|  | H28 | 0.630±0.043 mg/ml |
|  | H226 | 0.889±0.049 mg/ml |
|  | H2452 | 0.440±0.047 mg/ml |
|  | H2052 | 0.512±0.013 mg/ml |
|  | VMC20 | 0.959±0.050 mg/ml |
|  | VMC23 | 0.663±0.066 mg/ml |
|  | VMC33 | 0.384±0.036 mg/ml |
|  | VMC40 | 0.473±0.032 mg/ml |
|  | MeT5A | 0.209±0.039 mg/ml |
|  | Humofib | 0.149±0.025 mg/ml |
| BLAGLET-high, 4.5 hours without FBS, then 1 day with FBS (plotted Figure 1b) | | |
|  | MM05 | 0.487±0.297 mg/ml |
|  | MSTO | 0.306±0.020 mg/ml |
|  | REN | 0.467±0.008 mg/ml |
|  | H28 | 0.495±0.030 mg/ml |
|  | H226 | 0.386±0.041 mg/ml |
|  | H2452 | 0.267±0.015 mg/ml |
|  | H2052 | 0.400±0.022 mg/ml |
|  | VMC20 | 0.994±0.192 mg/ml |
|  | VMC23 | 0.493±0.009 mg/ml |
|  | VMC33 | 0.176±0.016 mg/ml |
|  | VMC40 | 0.239±0.019 mg/ml |
|  | MeT5A | 0.099±0.024 mg/ml |
|  | Humofib | 0.086±0.015 mg/ml |
| Cisplatin, 3 days with FBS | | |
|  | MM05 | 6.664±0.355 μM |
|  | MSTO | 4.152±0.166 μM |
|  | REN | 2.253±0.185 μM |
|  | H28 | 3.311±0.106 μM |
|  | H226 | 3.276±0.199 μM |
|  | H2452 | 5.790±0.593 μM |
|  | H2052 | 8.312±0.383 μM |
|  | VMC20 | 1.505±0.200 μM |
|  | VMC23 | 10.186±0.685 μM |
|  | VMC33 | 2.240±0.180 μM |
|  | VMC40 | 4.528±0.726 μM |
|  | MeT5A | 1.935±0.155 μM |
|  | Humofib | 9.826±0.758 μM |
| Pemetrexed, 3 days with FBS | | |
|  | MM05 | 0.141±0.066 μM |
|  | REN | 0.103±0.018 μM |
|  | H28 | 0.213±0.045 μM |
|  | H226 | 0.295±0.038 μM |
|  | H2452 | 0.181±0.029 μM |
|  | H2052 | 0.464±0.116 μM * |
|  | VMC20 | 0.292±0.088 μM * |
|  | VMC23 | 0.306±0.009 μM * |
|  | VMC33 | 0.306±0.009 μM * |
|  | VMC40 | 0.230±0.074 μM |
|  | MeT5A | 0.229±0.047 μM |
|  | Humofib | 2.791±2.011 μM * |
| Gemcitabine, 3 days with FBS | | |
|  | MM05 | 0.009±0.001 μM |
|  | REN | 0.023±0.003 μM |
|  | H28 | 0.006±0.001 μM |
|  | H226 | 0.018±0.001 μM |
|  | H2452 | 0.023±0.001 μM |
|  | H2052 | 0.021±0.008 μM |
|  | VMC20 | 0.009±0.000 μM |
|  | VMC23 | 0.011±0.003 μM |
|  | VMC33 | 0.008±0.001 μM |
|  | VMC40 | 0.004±0.001 μM |
|  | MeT5A | 0.010±0.001 μM |
|  | Humofib | TC_50_ not reached |
| Vinorelbine, 3 days with FBS | | |
|  | MSTO | 0.040±0.099 μM |
|  | REN | 0.001±0.000 μM |
|  | H28 | 0.009±0.001 μM |
|  | H226 | 0.014±0.002 μM |
|  | H2452 | 0.003±0.001 μM |
|  | H2052 | 0.059±0.057 μM |
|  | VMC20 | 0.036±0.003 μM |
|  | VMC23 | 0.006±0.003 μM |
|  | VMC33 | 0.005±0.001 μM |
|  | VMC40 | 0.002±0.000 μM |
|  | MeT5A | 0.002±0.001 μM |
|  | Humofib | TC_50_ not reached |

*Supplementary Table B. Mean TC_50_ at which 50% of rat cells remain viable after in vitro BAMLET or chemotherapy treatment. Plated cells were incubated for 3 days with BAMLET or chemotherapy in medium with fetal bovine serum (FBS) and without removal of conditioned medium after which the cell death assay was carried out. The treatment of zero concentration was used as control for calculating % viability. Each one experiment of three replicates was performed once a week on the same cell culture. Chemotherapy-resistant cell lines were dosed once a week with chemotherapy to maintain the resistance.*

| **Treatment** | | |
| --- | --- | --- |
|  | **Cell line** | **TC_50_** |
| BAMLET-medium | | |
|  | IL45-CisR (high dose maintenance) | 0.873±0.209 mg/ml |
|  | IL45-CisR (low dose maintenance) | 1.062±0.303 mg/ml |
|  | IL45-PemR (high dose maintenance) | 0.995±0.258 mg/ml |
|  | IL45-PemR (low dose maintenance) | 0.977±0.180 mg/ml |
|  | IL45-GemR (high dose maintenance) | 1.228±0.757 mg/ml |
|  | IL45-GemR (low dose maintenance) | 1.070±0.219 mg/ml |
|  | IL45-VLBR (high dose maintenance) | 0.929±0.267 mg/ml |
|  | IL45-VLBR (low dose maintenance) | 0.810±0.072 mg/ml |
|  | Parental IL45 (higher passage no.) | 0.877±0.178 mg/ml |
|  | Parental IL45 (lower passage no.) | 0.873±0.182 mg/ml |
|  | 4/4RM.4 (non-cancer immortalised) | 0.828±0.081 mg/ml |
| Cisplatin | | |
|  | IL45-CisR (high dose maintenance) | 3.179±1.897 μM |
|  | IL45-CisR (low dose maintenance) | 4.222±2.834 μM |
|  | Parental IL45 (higher passage no.) | 1.739±0.308 μM |
|  | Parental IL45 (lower passage no.) | 1.910±0.837 μM |
|  | 4/4RM.4 (non-cancer immortalised) | 4.463±7.815 μM |
| Pemetrexed | | |
|  | IL45-PemR (high dose maintenance) | TC_50_ not reached |
|  | IL45-PemR (low dose maintenance) | TC_50_ not reached |
|  | Parental IL45 (higher passage no.) | 8.004±7.071 μM |
|  | Parental IL45 (lower passage no.) | 4.987±4.402 μM |
|  | 4/4RM.4 (non-cancer immortalised) | TC_50_ not reached |
| Gemcitabine | | |
|  | IL45-GemR (high dose maintenance) | 1.425±1.404 μM |
|  | IL45-GemR (low dose maintenance) | 0.964±0.161 μM |
|  | Parental IL45 (higher passage no.) | 0.462±0.347 μM |
|  | Parental IL45 (lower passage no.) | 0.356±0.145 μM |
|  | 4/4RM.4 (non-cancer immortalised) | 0.311±0.080 μM |

*Supplementary Table C. Gene expression fold change results measured by qPCR for ATP synthase and RNA18S1 genes for human mesothelioma cells receiving regular BAMLET dosing compared to controls receiving no dosing. BR0.1 indicates regular dosing of 0.1 mg/ml. BR0.3 indicates regular dosing of 0.3 mg/ml.*

| **Target Gene** | **Cell Line** | **Fold Change** | **Result** | **Statistically Significant Result** | **p-value** |
| --- | --- | --- | --- | --- | --- |
| ATP5G1V2 | H28-BR0.1 | 1.662 | UP |  | 0.1072 |
| ATP5G1V2 | H28-BR0.3 | 0.204 | DOWN | DOWN | 0.0095 |
| ATP5G1V2 | MeT5A-BR0.1 | 0.0097 | DOWN | DOWN | 0.0457 |
| ATP5G1V2 | MeT5A-BR0.3 | 0.111 | DOWN |  | 0.0562 |
| ATP5G1V2 | REN-BR0.1 | 1.137 | UP |  | 0.3482 |
| ATP5G1V2 | REN-BR0.3 | 0.094 | DOWN | DOWN | 0.0336 |
| ATP5G1V2 | VMC23-BR0.3 | 0.132 | DOWN | DOWN | 0.0446 |
| ATP5G2 | H28-BR0.1 | 0.000 | DOWN |  | 0.8458 |
| ATP5G2 | H28-BR0.3 | 0.000 | DOWN |  | 0.9452 |
| ATP5G2 | MeT5A-BR0.1 | 0.000 | DOWN |  | 0.7939 |
| ATP5G2 | MeT5A-BR0.3 | 0.000 | DOWN |  | 0.7971 |
| ATP5G2 | REN-BR0.1 | 1.600 | UP |  | 0.1772 |
| ATP5G2 | REN-BR0.3 | 0.086 | DOWN | DOWN | 0.0393 |
| ATP5G2 | VMC23-BR0.3 | 0.282 | DOWN |  | 0.0819 |
| ATP5G2V3 | H28-BR0.1 | 0.668 | DOWN |  | 0.2004 |
| ATP5G2V3 | H28-BR0.3 | 0.000 | DOWN |  | 0.5020 |
| ATP5G2V3 | MeT5A-BR0.1 | 0.000 | DOWN |  | 0.9463 |
| ATP5G2V3 | MeT5A-BR0.3 | 0.000 | DOWN |  | 0.9489 |
| ATP5G2V3 | REN-BR0.1 | 0.643 | DOWN |  | 0.2217 |
| ATP5G2V3 | REN-BR0.3 | 0.481 | DOWN |  | 0.1247 |
| ATP5G2V3 | VMC23-BR0.3 | 0.000 | DOWN |  | 0.5497 |
| ATP5G3 | H28-BR0.1 | 8.605 | UP | UP | 0.0202 |
| ATP5G3 | H28-BR0.3 | 0.669 | DOWN |  | 0.2116 |
| ATP5G3 | MeT5A-BR0.1 | 0.688 | DOWN |  | 0.2932 |
| ATP5G3 | MeT5A-BR0.3 | 2.426 | UP |  | 0.0571 |
| ATP5G3 | REN-BR0.1 | 0.804 | DOWN |  | 0.2840 |
| ATP5G3 | REN-BR0.3 | 0.199 | DOWN | DOWN | 0.0362 |
| ATP5G3 | VMC23-BR0.3 | 0.347 | DOWN |  | 0.0803 |
| ATP5B | H28-BR0.1 | 0.000 | DOWN |  | 1.0000 |
| ATP5B | H28-BR0.3 | 0.000 | DOWN |  | 1.0000 |
| ATP5B | MeT5A-BR0.1 | 0.000 | DOWN |  | 1.0000 |
| ATP5B | MeT5A-BR0.3 | 0.000 | DOWN |  | 1.0000 |
| ATP5B | REN-BR0.1 | 0.869 | DOWN |  | 0.3811 |
| ATP5B | REN-BR0.3 | 0.000 | DOWN |  | 0.5092 |
| ATP5B | VMC23-BR0.3 | 0.321 | DOWN |  | 0.1615 |
| ATP5IF1 | H28-BR0.1 | 0.895 | DOWN |  | 0.3262 |
| ATP5IF1 | H28-BR0.3 | 0.294 | DOWN | DOWN | 0.0104 |
| ATP5IF1 | MeT5A-BR0.1 | 0.007 | DOWN | DOWN | 0.0462 |
| ATP5IF1 | MeT5A-BR0.3 | 0.001 | DOWN |  | 0.0817 |
| ATP5IF1 | REN-BR0.1 | 0.821 | DOWN |  | 0.3347 |
| ATP5IF1 | REN-BR0.3 | 0.152 | DOWN | DOWN | 0.0369 |
| ATP5IF1 | VMC23-BR0.3 | 0.453 | DOWN |  | 0.1632 |
| RNA18S1 | H28-BR0.1 | 0.721 | DOWN |  | 0.2336 |
| RNA18S1 | H28-BR0.3 | 0.224 | DOWN | DOWN | 0.0107 |
| RNA18S1 | MeT5A-BR0.1 | 0.004 | DOWN | DOWN | 0.0414 |
| RNA18S1 | MeT5A-BR0.3 | 0.000 | DOWN |  | 0.0504 |
| RNA18S1 | REN-BR0.1 | 0.704 | DOWN |  | 0.1624 |
| RNA18S1 | REN-BR0.3 | 0.321 | DOWN | DOWN | 0.0389 |
| RNA18S1 | VMC23-BR0.3 | 0.397 | DOWN |  | 0.1197 |

*Supplementary Table D. Radius of gyration (Rg), intensity extrapolated to zero angle, I(0), and maximum molecular dimension (Dmax) for BAMLET species at pH 12 and 4°C and BLAGLET species at pH 12 in 2% β-mercaptoethanol at 10°C, derived from SAXS. Rg calculated by Guinier analysis (Guinier 1938) and by the numerical Indirect Method of Glatter (Glatter 1977). I(0) and Dmax calculated by the numerical method. BAMLET measurements are on absolute scale.*

| **Species** | **Protein to Lipid Molecular Ratio** | **Conc.**  **(mg/ml)** | **I(0)/conc.**  **(cm^-1^)** | **R_g_, Guinier**  **(Å)** | **R_g_, real space**  **(Å)** | **D_max_**  **(Å)** |
| --- | --- | --- | --- | --- | --- | --- |
| BAMLET | 1 : 2 | 0.5 | 0.0170±  0.00115 | 20.2±  1.27 | 28.33±  1.708 | 96 |
| BAMLET | 1 : 2 | 2 | 0.0159±  0.000187 | 23.5±  0.482 | 24.94±  0.390 | 95 |
| BAMLET | 1 : 2 | 4 | 0.0151±  0.000111 | 22.6±  0.299 | 24.62±  0.234 | 95 |
| BAMLET | 1 : 5 | 0.5 | 0.0164±  0.000694 | 22.0±  1.14 | 25.62±  1.379 | 95 |
| BAMLET | 1 : 5 | 4 | 0.0159±  0.000104 | 23.8±  0.285 | 24.66±  0.222 | 95 |
| BAMLET | 1 : 5.625  (equal mix of  1 : 5 and 1 : 6.25) | 4 | 0.0155±  0.000075 | 23.5±  0.288 | 24.35±  0.149 | 90 |
| BAMLET | 1 : 6:25  (equal mix of  1 : 5 and 1 : 7.5) | 4 | 0.0158±  0.000044 | 23.7±  0.286 | 24.20±  0.071 | 81 |
| BAMLET | 1 : 7.5  (equal mix of  1 : 5 and 1 : 10) | 4 | 0.0162±  0.000075 | 23.9±  0.297 | 25.01±  0.150 | 92 |
| BAMLET | 1 : 8.75  (equal mix of  1 : 7.5 and 1 : 10) | 4 | 0.0172±  0.00005 | 24.9±  0.274 | 25.46±  0.088 | 88 |
| BAMLET | 1 : 10 | 0.5 | 0.0175±  0.000275 | 27.0±  1.34 | 27.78±  0.512 | 95 |
| BAMLET | 1 : 10 | 2 | 0.0174±  0.0000764 | 26.6±  0.411 | 26.99±  0.140 | 91 |
| BAMLET | 1 : 10 | 4 | 0.0172±  0.0001356 | 26.8±  0.268 | 26.34±  0.055 | 86 |
| BAMLET | 1 : 12.5  (equal mix of  1 : 10 and 1 : 15) | 4 | 0.0168±  0.000039 | 26.6±  0.277 | 26.14±  0.064 | 86 |
| BAMLET | 1 : 15  (equal mix of  1 : 10 and 1 : 20) | 4 | 0.0162  0.000031 | 27.2±  0.307 | 26.12±  0.051 | 82 |
| BAMLET | 1 : 17.5  (equal mix of  1 : 15 and 1 : 20) | 4 | 0.0160±  0.000043 | 26.6±  0.236 | 26.34±  0.072 | 86 |
| BAMLET | 1 : 20 | 0.5 | 0.0.0161±  0.000308 | 27.4±  1.81 | 27.99±  0.573 | 92 |
| BAMLET | 1 : 20 | 2 | 0.0151±  0.0000792 | 29.7±  0.530 | 27.49±  0.185 | 95 |
| BAMLET | 1 : 20 | 4 | 0.0153±  0.000042 | 26.9±  0.309 | 26.57±  0.082 | 89 |
| BLAGLET(*β-lactoglobulin)* | 1:0 | 0.25 | 0.1838±  0.0118 | 21.9±  4.73 | 24.71±  1.565 | 84 |
| BLAGLET(*β-lactoglobulin)* | 1:0 | 0.5 | 0.1485±  0.00551 | 20.7±  2.00 | 23.18±  0.990 | 82 |
| BLAGLET(*β-lactoglobulin)* | 1:0 | 1 | 0.1283±  0.000767 | 20.6±  0.201 | 21.43±  0.241 | 80 |
| BLAGLET(*β-lactoglobulin)* | 1:0 | 2 | 0.1236±  0.000507 | 20.7±  0.129 | 21.39±  0.170 | 80 |
| BLAGLET(*β-lactoglobulin)* | 1:0 | 4 | 0.1314±  0.000258 | 21.6±  0.0714 | 22.31±  0.071 | 80 |
| BLAGLET | 1:20 | 0.25 | 0.3022±  0.00366 | 30.7±  1.16 | 30.09±  0.371 | 88 |
| BLAGLET | 1:20 | 0.5 | 0.2810±  0.00273 | 31.1±  0.796 | 30.42±  0.261 | 88 |
| BLAGLET | 1:20 | 1 | 0.2744±  0.00172 | 32.0±  0.624 | 30.96±  0.182 | 92 |
| BLAGLET | 1:20 | 2 | 0.2724±  0.00101 | 32.0±  0.395 | 31.21±  0.100 | 92 |
| BLAGLET | 1:20 | 4 | 0.2690±  0.000555 | 31.6±  0.290 | 31.49±  0.048 | 88 |
| BLAGLET | 1:40 | 0.25 | 0.2579±  0.00419 | 31.4±  1.75 | 31.77±  0.421 | 89 |
| BLAGLET | 1:40 | 0.5 | 0.2434±  0.00311 | 31.0±  1.25 | 32.26±  0.323 | 89 |
| BLAGLET | 1:40 | 1 | 0.2370±  0.00196 | 31.1±  1.28 | 32.70±  0.222 | 92 |
| BLAGLET | 1:40 | 2 | 0.2299±  0.000956 | 30.9±  0.650 | 32.49±  0.089 | 85 |
| BLAGLET | 1:40 | 4 | 0.2486±  0.000984 | 30.4±  1.13 | 33.24±  0.078 | 89 |
| BLAGLET | 1:60 | 0.25 | 0.2174±  0.00295 | 34.9±  1.91 | 33.60±  0.383 | 88 |
| BLAGLET | 1:60 | 0.5 | 0.2182±  0.00225 | 35.6±  2.79 | 35.40±  0.229 | 92 |
| BLAGLET | 1:60 | 1 | 0.2135±  0.00140 | 34.7±  1.96 | 35.50±  0.134 | 90 |
| BLAGLET | 1:60 | 2 | 0.2173±  0.00141 | 33.3±  2.03 | 35.96±  0.123 | 92 |
| BLAGLET | 1:60 | 4 | 0.2271±  0.000689 | 29.4±  0.622 | 35.41±  0.057 | 92 |
| BLAGLET | 1:80 | 0.25 | 0.2394±  0.00435 | 33.7±  3.90 | 35.92±  0.382 | 90 |
| BLAGLET | 1:80 | 0.5 | 0.2168±  0.0023 | 34.2±  2.28 | 36.00±  0.211 | 90 |
| BLAGLET | 1:80 | 1 | 0.2110±  0.00121 | 35.3±  1.66 | 36.46±  0.105 | 90 |
| BLAGLET | 1:80 | 2 | 0.2072±  0.000969 | 33.9±  1.84 | 36.43±  0.083 | 90 |
| BLAGLET | 1:80 | 4 | 0.1968±  0.00245 |  | 34.62±  0.118 | 90 |

*Supplementary Table E. Primers used for qPCR experiments.*

| **Target** | **Forward** | **Reverse** |
| --- | --- | --- |
| RNA18S1 | 5'-gcaattattccccatgaacg-3' | 5'-gggacttaatcaacgcaagc-3' |
| ATP5G1V2 | 5'-agggctaaagctgggagact-3' | 5'-tgtgtctgcctccttcttga-3' |
| ATP5G2 | 5'-gtgatgcctgagctgatcct-3' | 5'-tggagcaggcgaacattt-3' |
| ATP5G2V3 | 5'-ttttaactcaccttctgtttgagc-3' | 5'-atgttcgcctgctccaagt-3' |
| ATP5G3 | 5'-aacgtgcattcaggcagat-3' | 5'-acattggaatatttttcatcagtgg-3' |
| ATP5B | 5'-agaggtcccatcaaaaccaa -3' | 5'-tcctgctcaacactcatttcc-3' |
| ATP5IF1 | 5'-gggccttcggaaagagag-3' | 5'-ttcaaagctgccagttgttc-3' |

| 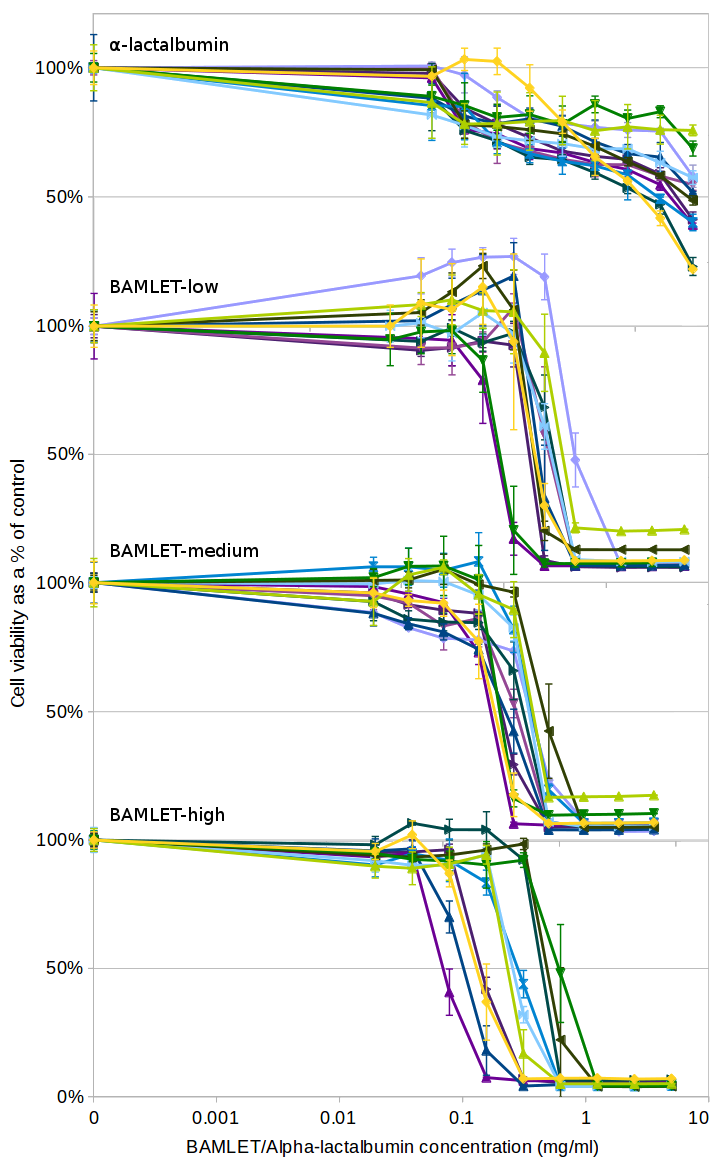  (a) | 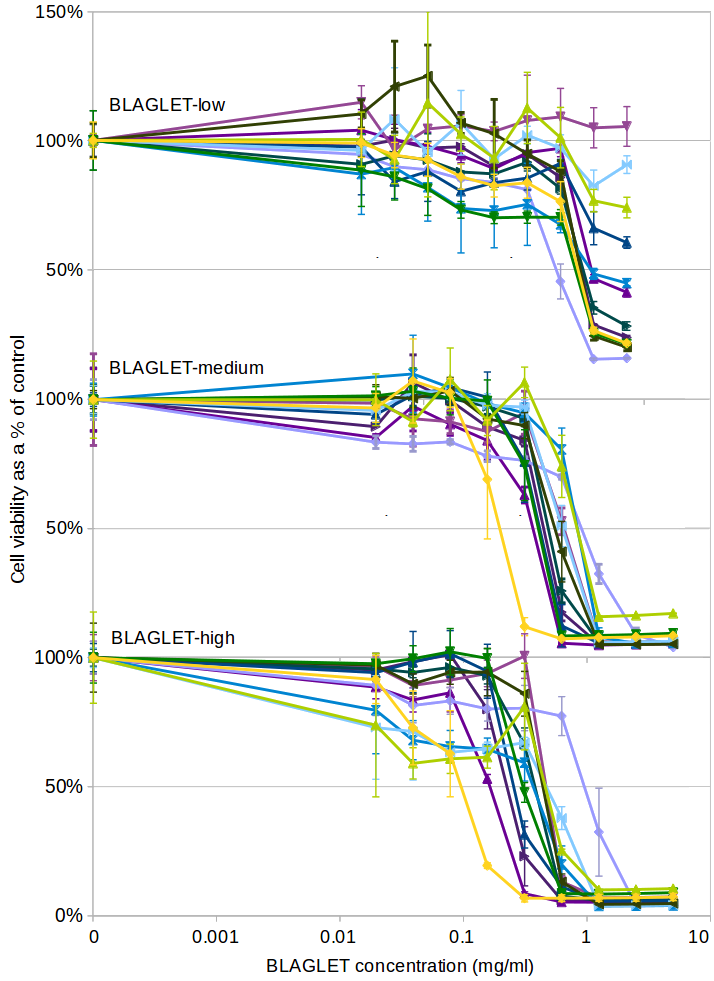  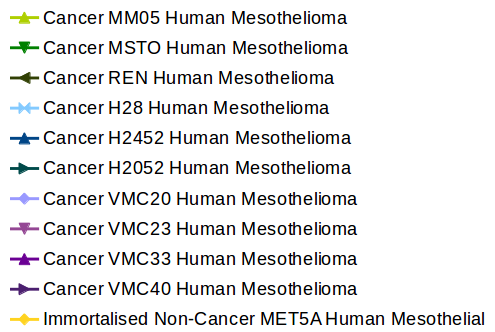  (b) |
| --- | --- |

*Supplementary Figure A. Cell death assay plots for human mesothelioma and non-cancer cells showing that the higher the amount of oleic acid in the BAMLET or BLAGLET compound, the more sensitive are cells to treatment. (a) BAMLET or bovine α-lactalbumin for 4.5 hours in medium without FBS after removal of conditioned FBS-containing medium, then addition of FBS and incubation for 1 day; (b) BLAGLET for 4.5 hours following same protocol as in (a). A representative experiment is shown.*

| 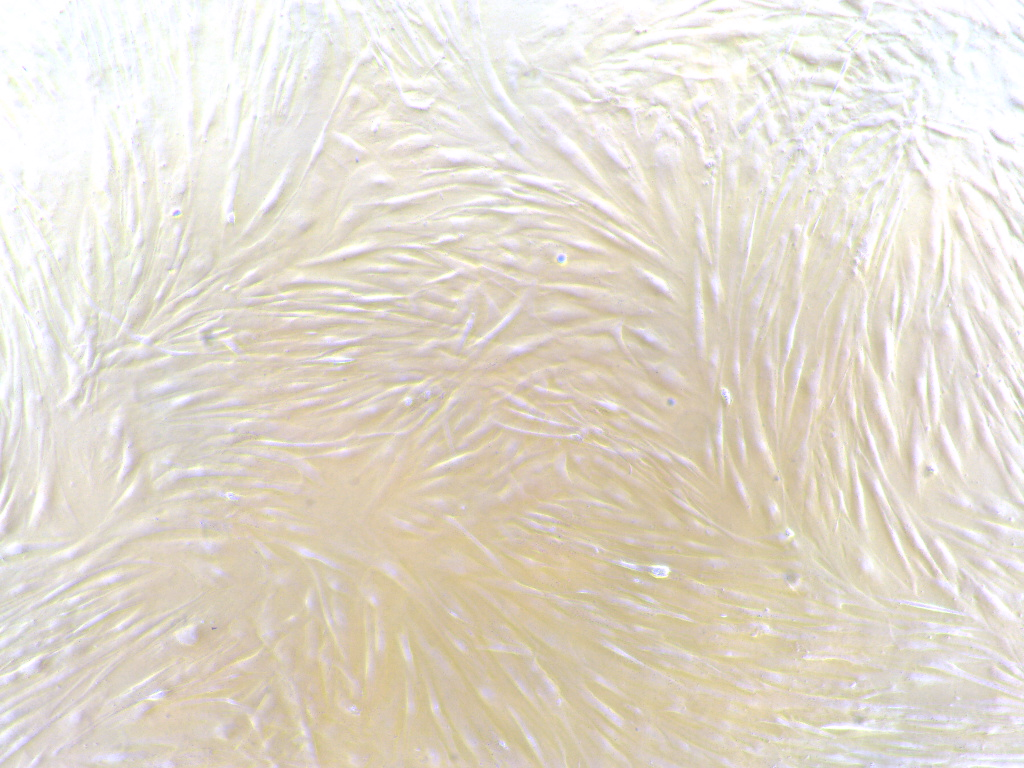(a) | 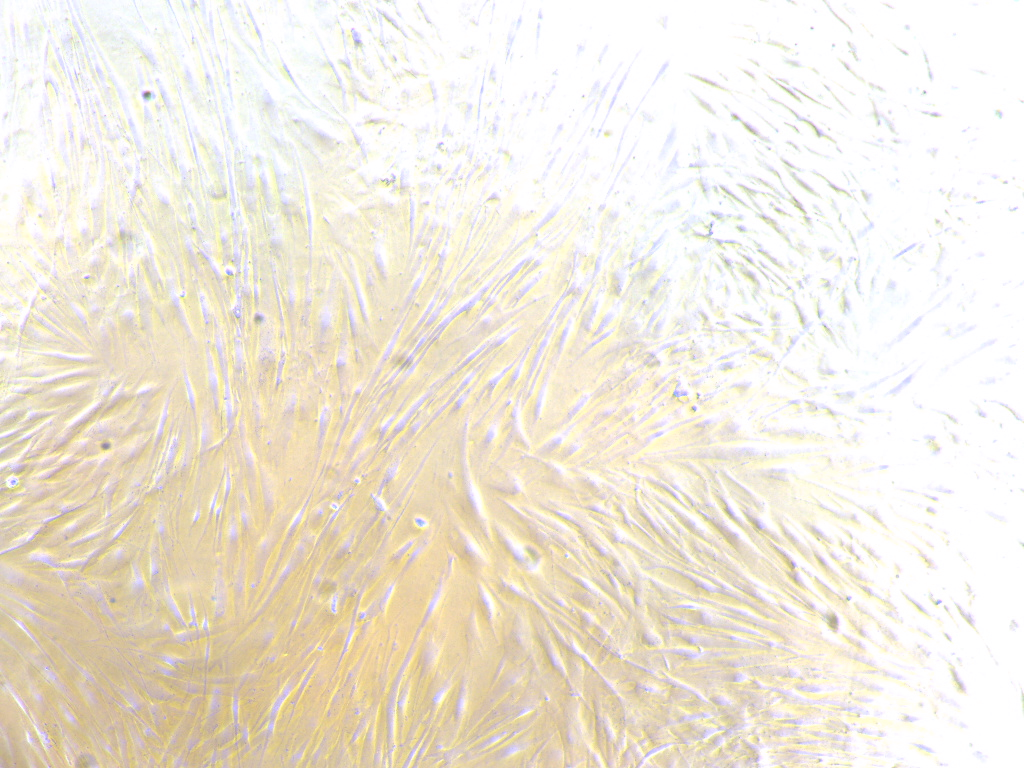(b) |
| --- | --- |

*Supplementary Figure B. Light microscopy images (Leica) of (a) HOFM01 and (b) HOFM02 non-cancer human primary fibroblasts.*

| 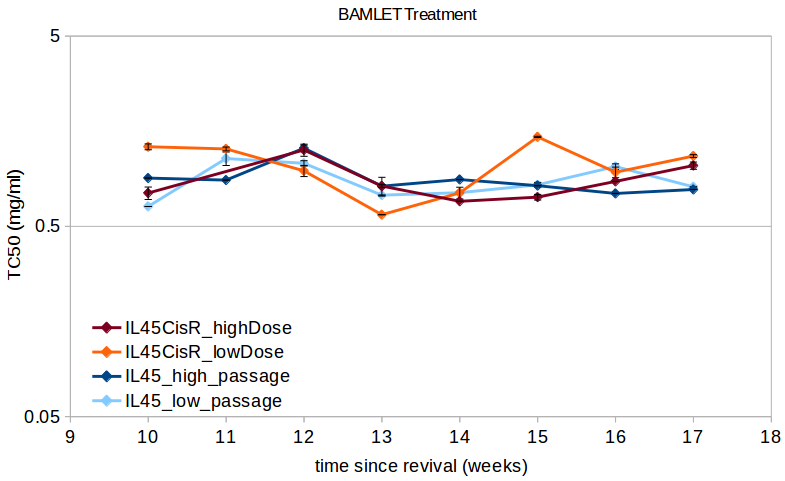  (a) | 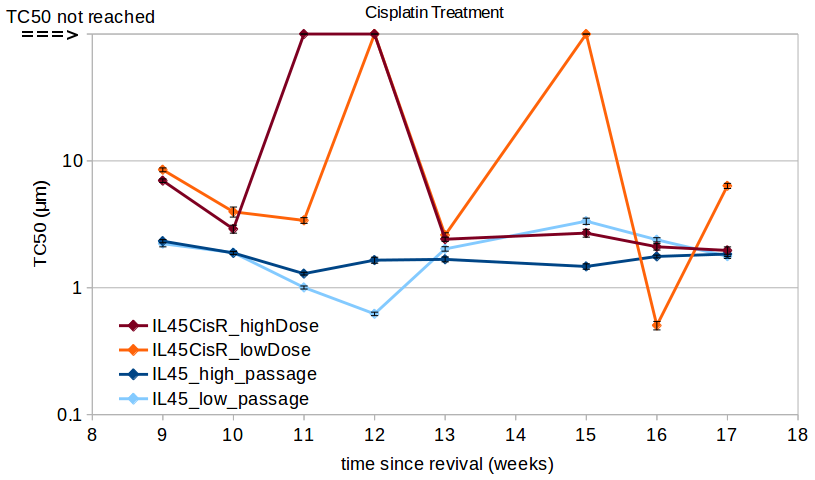  (b) |
| --- | --- |
| 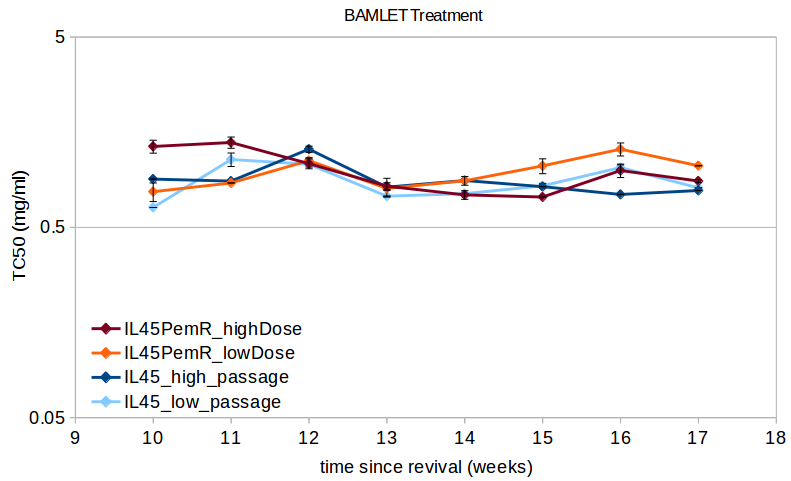  (c) | 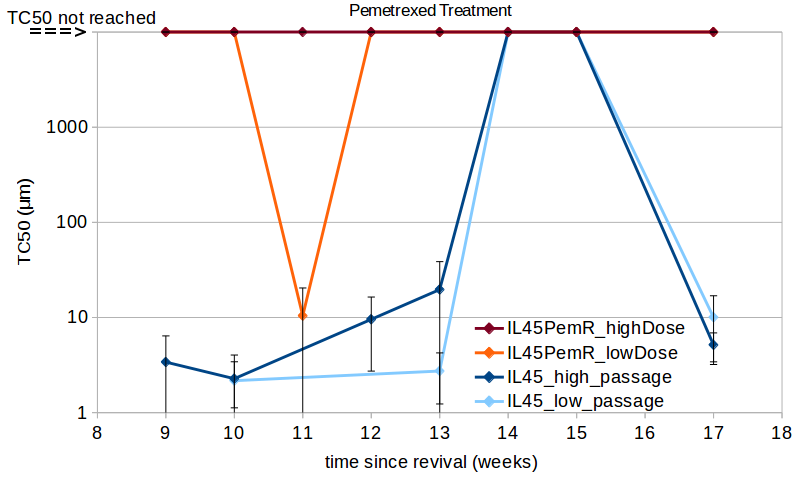  (d) |
| 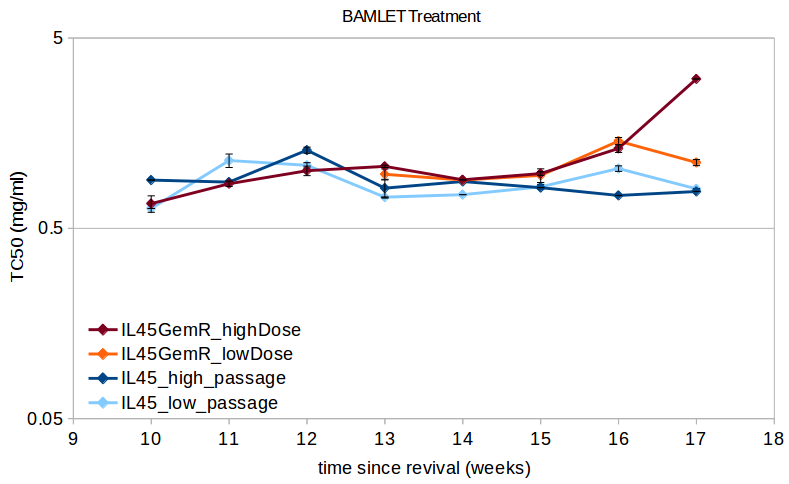  (e) | 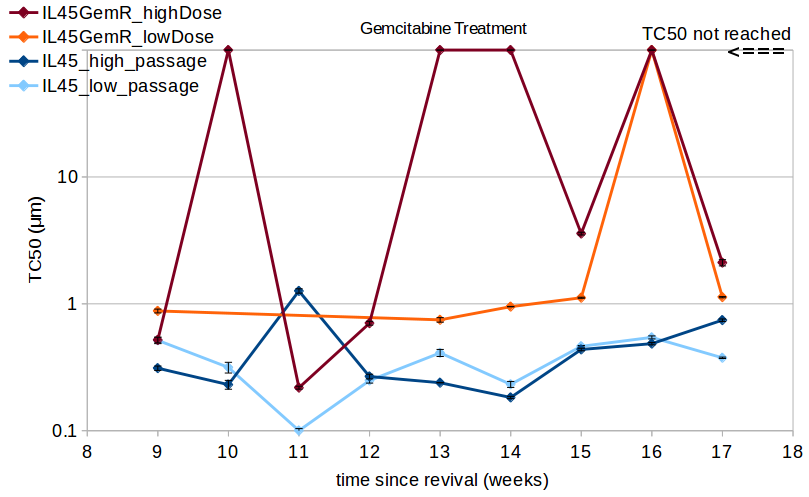  (f) |
| 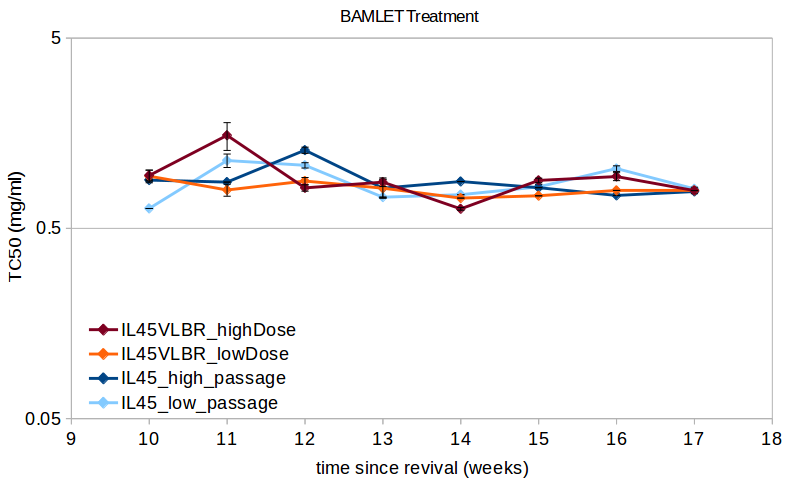  (g) |  |

*Supplementary Figure C. TC_50_ values for (a-b) cisplatin-resistant (IL45-CisR), (c-d) pemetrexed-resistant (IL45-PemR), (e-f) gemcitabine-resistant (IL45-GemR), and (g) vinorelbine-resistant (IL45-VLBR) rat mesothelioma cell lines (red lines) and their parental rat mesothelioma cell line (IL45, blue lines) for treatment with (b) cisplatin, (d) pemetrexed, (f) gemcitabine, and (a,c,e,g) BAMLET-medium. Cell death assays were performed once a week to monitor drug sensitivities, and involved adding drug compound to cell medium, incubating for 3 days, then measuring cell viability by fluorescence assay.*

| 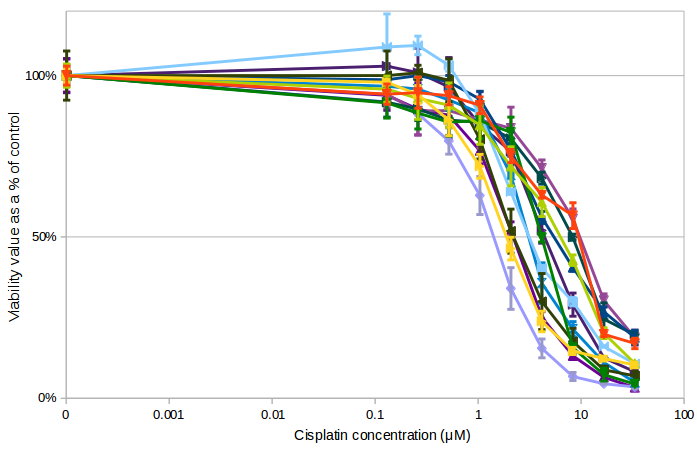  (a) | 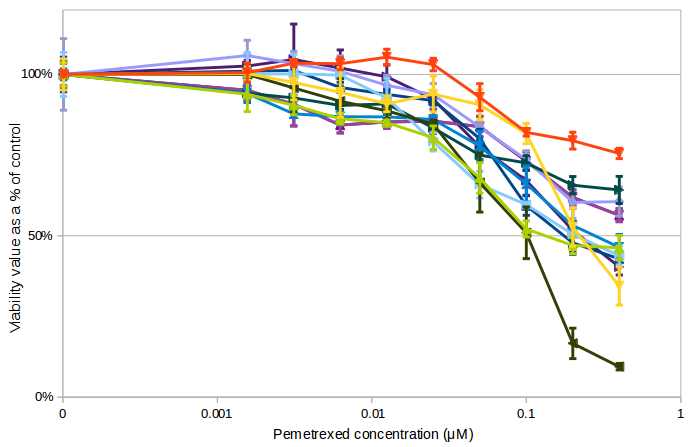  (b) |
| --- | --- |
| 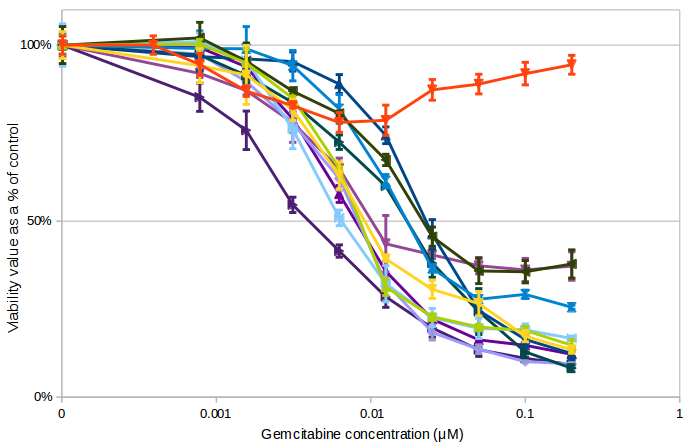  (c) | 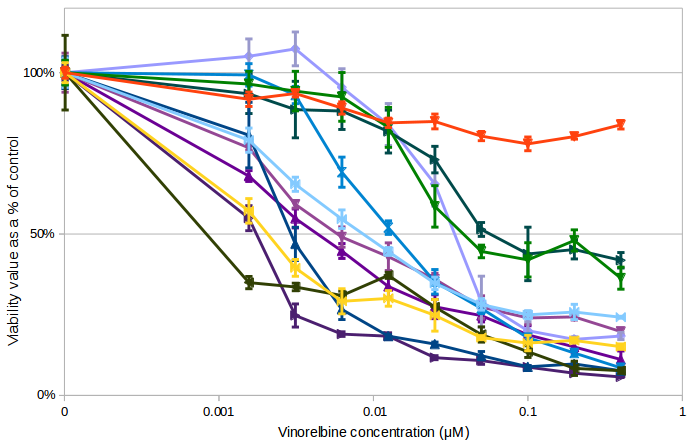  (d) |
| 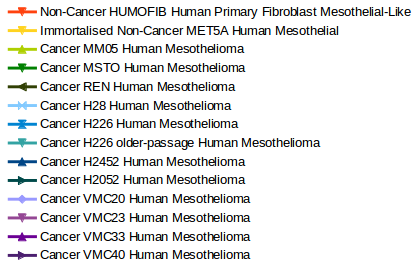 | |

*Supplementary Figure D. Cell death assay plots for human mesothelioma and non-cancer cells treated with (a) cisplatin, (b) pemextrexed, (c) gemcitabine, and (d) vinorelbine followed by 3 days incubation. Experiments were carried out 3 times and values shown are for a representative experiment of three replicates.*

**(a)**

**subunit: I A C B delta alpha gamma beta epsilon**

**
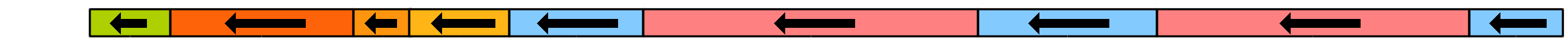
**

**(b)**

**subunit: C A B delta alpha gamma beta epsilon**


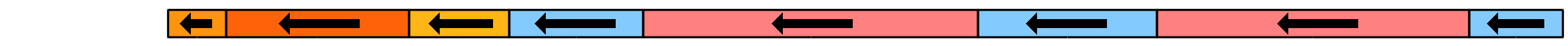


**(c)**

CLUSTAL O(1.2.3) multiple sequence alignment for F0F1 ATP synthase subunit I

S.pneumonaie No significant similarity found (BLASTP 2.6.0+)

S.mitis No significant similarity found (BLASTP 2.6.0+)

S.pyogenes No significant similarity found (BLASTP 2.6.0+)

S.aureus ----MSRFYNIFKQYIQYYLYLLIILGGLYLF-THHAFILGLIIGVTGSAINTVIFESYL

S.epidermidis ----MRRFNIIFKQYLQYYLYILVIIGILYSI-SPHPFLLGLMIGTCGSLVNTYIFEIYL

P.aeruginosa MESRMPSRLPAFRLLLV--QLVVVLVAAISLWISWGPV-AG-YSGLLGGMIAWLPNCYFA

E.coli MSVSLVSRNVARKLLLV--QLLVVIASG-LLFSLKDPF-WG-VSAISGGLAVFLPNVLFM

K.pneumoniae MSVSLLSRNVARKLLFI--QLLAVMASG-LLFSLKDPF-WG-ISAVCGGLAVVLPNLMFI

E.cloacae MSVSLLSRNVARKLLFI--QFLAVIASG-LLFSLKDPF-WG-ISAVCGGLAVVLPNMLFM

: : : : :: . . * . *. :

S.aureus AKAKRPDTMHISTG-NMWRYLVAIIACMIWYFNKSHVS-----------IIG-IIIGLMI

S.epidermidis AKSMHKETTQMSTG-STWRYLVAVIACVLWLFFKEHIN-----------IIG-VLIGLMI

P.aeruginosa YKAFRFSGARAAR-EIVRSFYAGEAGKLILTAVLFALTFAGVKPLMAPALFGVYLLTLMV

E.coli IFAWRHQAHTPAKGRVAWTFAFGEAFKVLAMLVLLVVALAVLKAV---------FLPLIV

K.pneumoniae IFAWRHQAHTPAKGRVAWTFAFGEAFKVLLTFALLAVALAVLKVV---------FLPLIV

E.cloacae IFAWRHQAHTPAKGRVAWSFALGEVCKVLLTFALLVMALAVLKVV---------FMPLIA

: : . : : . :: : :: *:

S.aureus SYVVVIIRPLLKVS----K-

S.epidermidis SYIVIILRPLLQRE------

P.aeruginosa SWCAPLLMGKTFTRP-----

E.coli TWVLVLVVQILAPAVINNKG

K.pneumoniae TWVLVLVVQVLAPAVINNKG

E.cloacae TWVLVLVVQVLAPAVINNKG

:: ::

*Supplementary Figure E. Protein multiple sequence comparisons. (a) F0F1 ATP synthase gene placements on bacterial chromosome for Escherichia coli str. K-12 substr. MG1655 (NCBI Reference Sequence: NZ_CP009685.1), Staphylococcus aureus subsp. aureus strain MRSA252 (GenBank: BX571856.1), Staphylococcus epidermidis ATCC 12228 (GenBank: AE015929.1), Klebsiella pneumoniae str. Kp52.145 (NCBI Reference Sequence: NZ_FO834906.1), Pseudomonas aeruginosa PA96 (NCBI Reference Sequence: NZ_CP007224.1), and Enterobacter cloacae strain GGT036 (NCBI Reference Sequence: NZ_CP009756.1). (b) F0F1 ATP synthase gene placements on bacterial chromosome for Streptococcus pneumoniae D39 (NCBI Reference Sequence: NC_008533.1), Streptococcus mitis strain SVGS_061 (GenBank: CP014326.1), and Streptococcus pyogenes M1 GAS (NCBI Reference Sequence: NC_002737.2). Subunit I is not present and subunit C is placed before subunit A. (c) Protein multiple sequence alignments, carried out by Clustal Omega (Sievers et al. 2011; Li et al. 2015; McWilliam et al. 2013) of F0F1 ATP synthase subunit I genes for E. coli, S. aureus, K. pneumoniae, P. aeruginosa and E. cloacae bacterial species in (a). Gene identifiers are WP_000116695.1, CAG41179.1, AAO05306.1, WP_004144991.1, WP_003458391.1, and WP_006177553.1 respectively.*


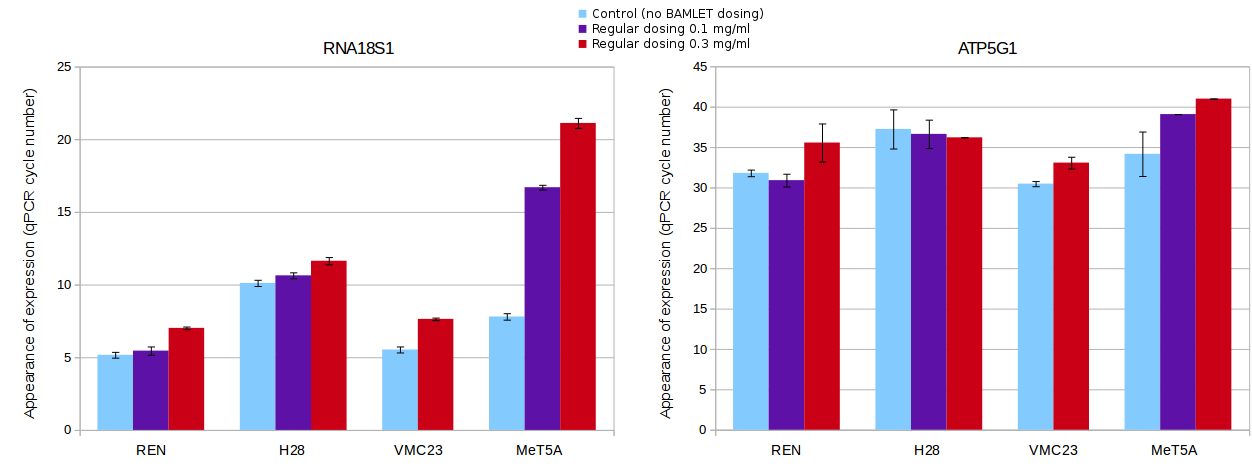


*Supplementary Figure F. qPCR expression levels of housekeeping RNA 18S ribosomal 1 gene (RNA18S1) and ATP synthase subunit c isoform 1 (ATP5G1). The higher the cycle number in which the gene RNA is detected by qPCR, the lower the amount of that gene present in the cells. We found that the higher the BAMLET dosing, the greater the downregulation of RNA18S1 as measured by qPCR cycle number, particularly for the control cell line MeT5A.*

| 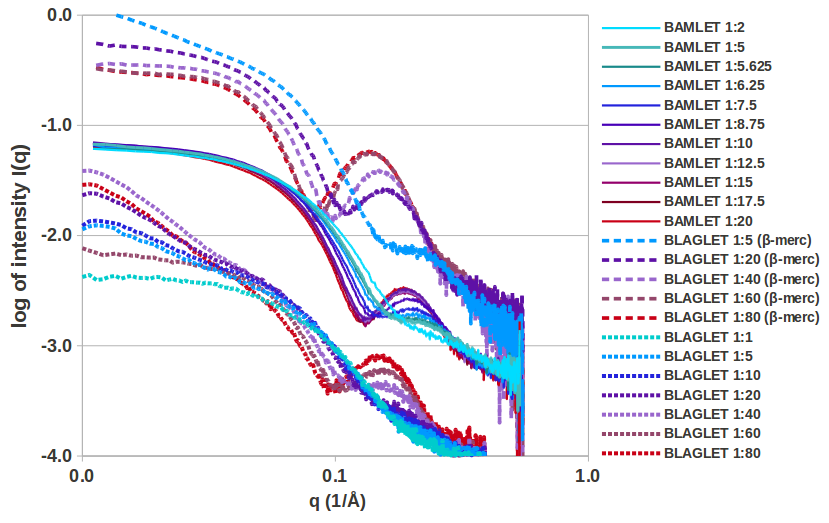(a) | |
| --- | --- |
| 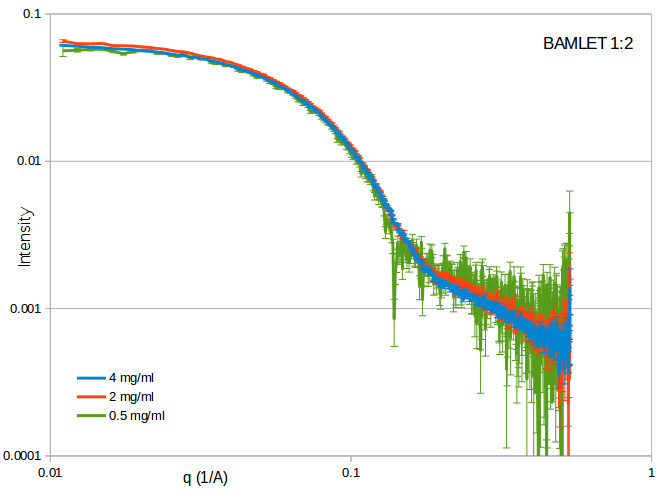(b) | 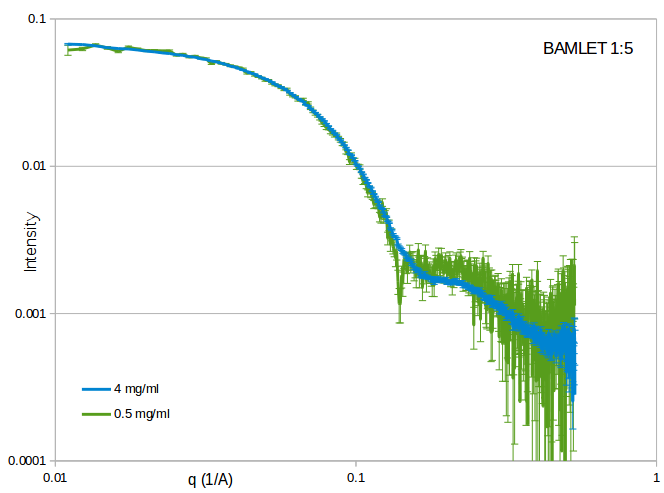(c) |
| 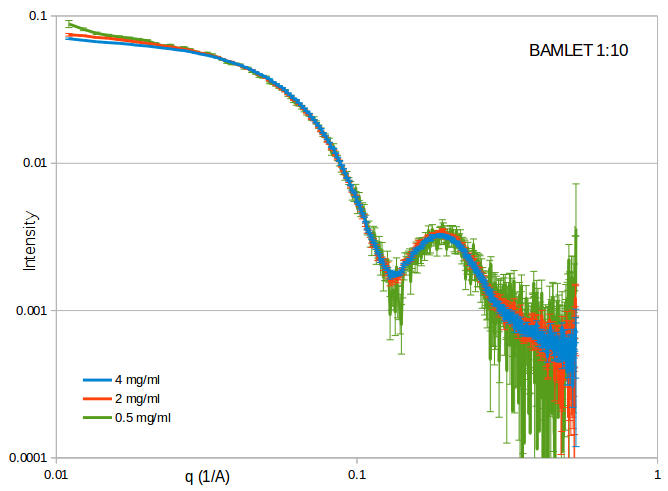(d) | 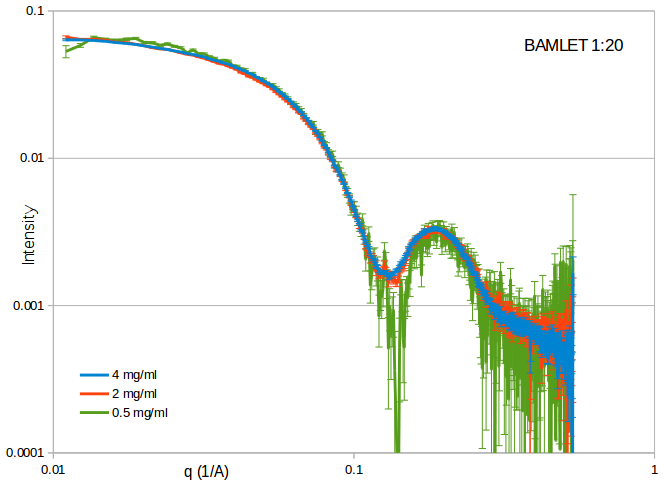(e) |
| 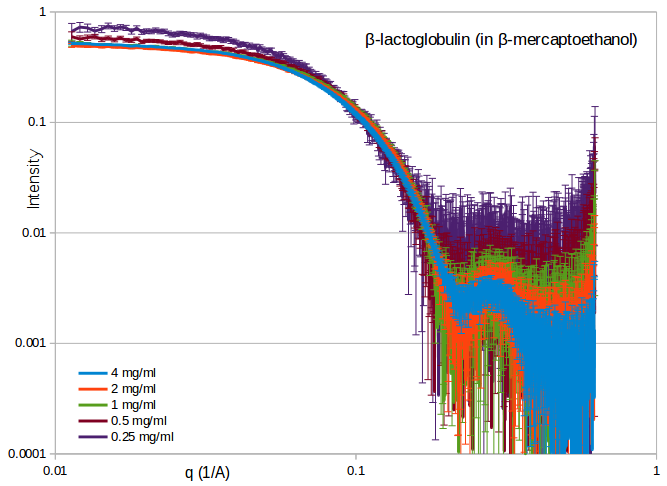  (f) | 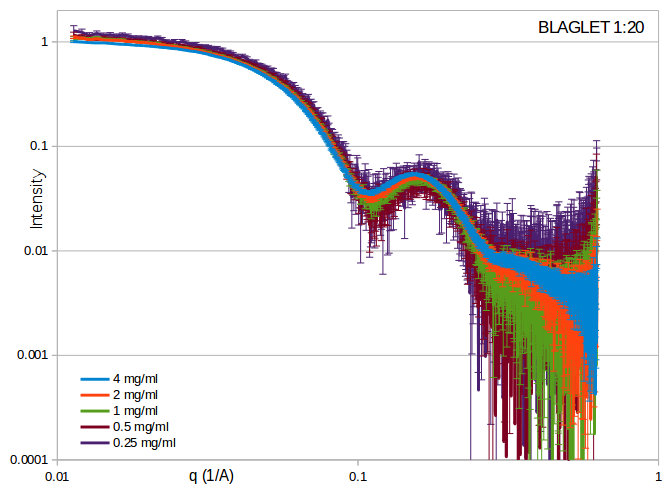  (g) |
| 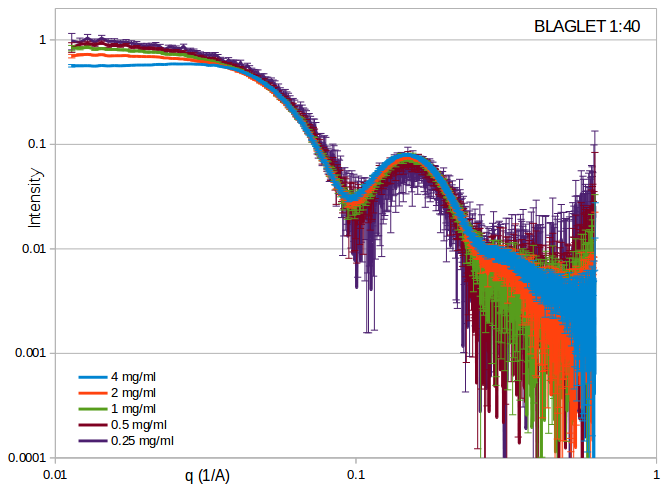(h) | 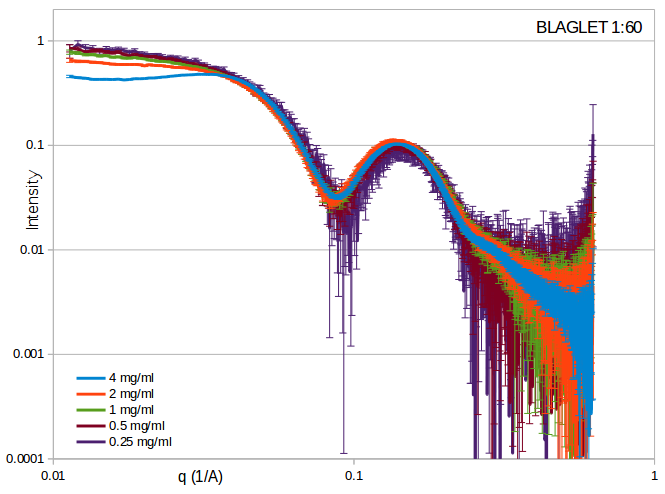(i) |
| 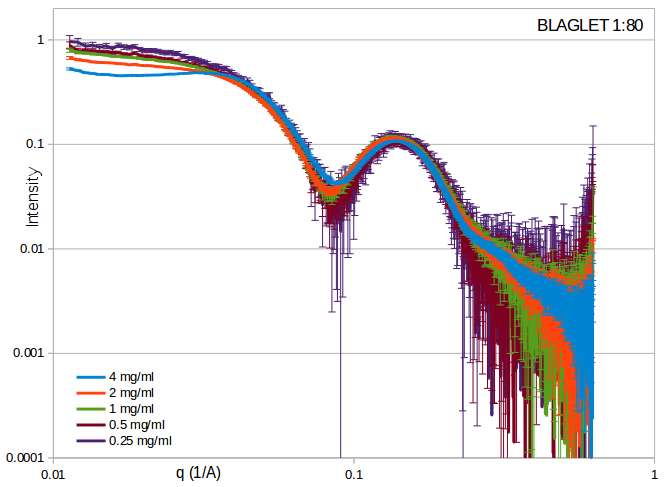  (j) |  |

*Supplementary Figure G. Experimental scattering curves with data scaled to be the same concentration per graph for each species demonstrating state of aggregation. (a) BAMLET and BLAGLET species in pH 12 that appear in Figure 3, shown here on an x-axis log scale that demonstrates the horizontal tendency of BAMLET (4°C) and BLAGLET in β-mercaptoethanol (10°C), indicating that complexes are not aggregated, and the increase in intensity at q → 0 for BLAGLET without β-mercaptoethanol (10°C) indicating that the complexes are aggregated. Concentration adjusted SAXS curves for BAMLET species in pH 12, 4°C, having input protein to lipid molecular ratios of (b) 1:2, (c) 1:5, (d) 1:10, and (e) 1:20, and BLAGLET species in pH 12, 2% β-mercaptoethanol, 10°C, having input protein to lipid molecular ratios of (f) 1:0 (β-lactoglobulin), (g) 1:20, (h) 1:40, (i) 1:60, and (j) 1:80, showing overlap when normalised by concentration, indicating that there is no concentration dependant aggregation or repulsion. The exceptions are the highest concentrations of BLAGLETs having high oleic acid content, and these low q points having intensity below the horizontal intensity curves of the lower concentrations, indicating intermolecular repulsion, were not included in analyses, and data for the lowest concentrations were used for modelling so as to avoid these intermolecular effects. The SAXS curves for the highest concentrations of BAMLET species exhibit no intermolecular aggregation or repulsion at low q and were used for modelling.*

| 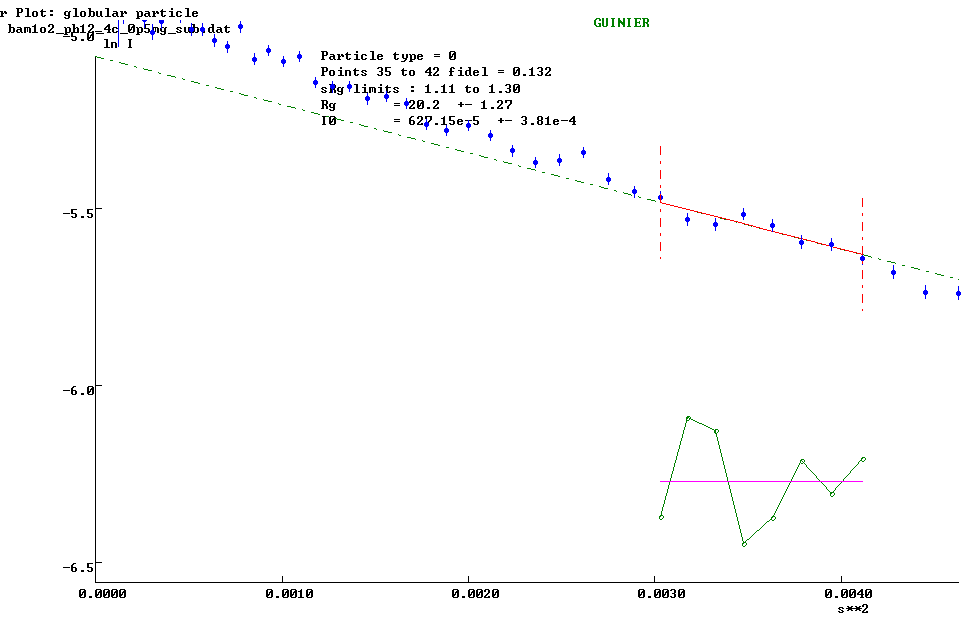(a) | 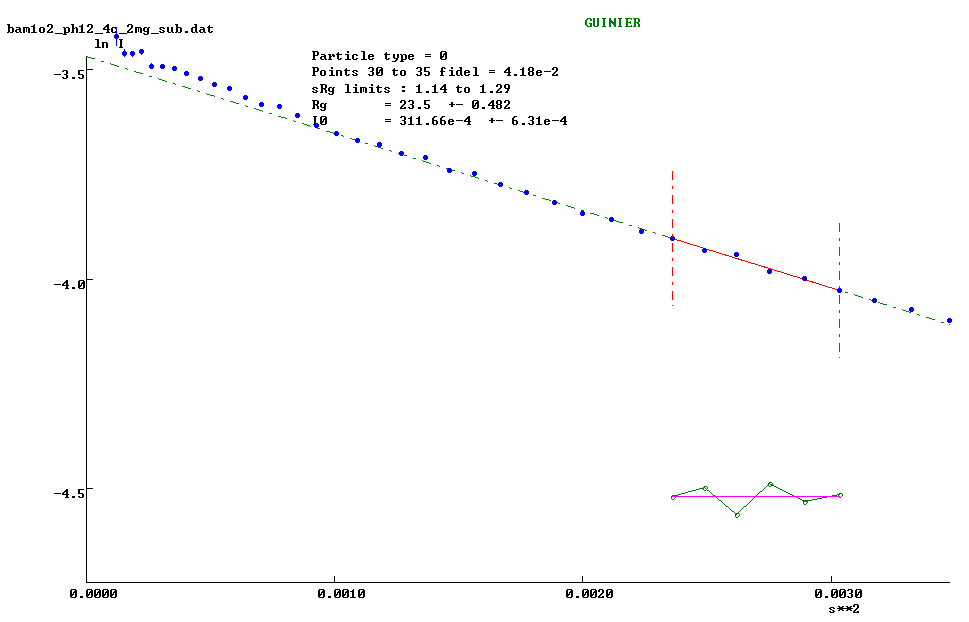 | 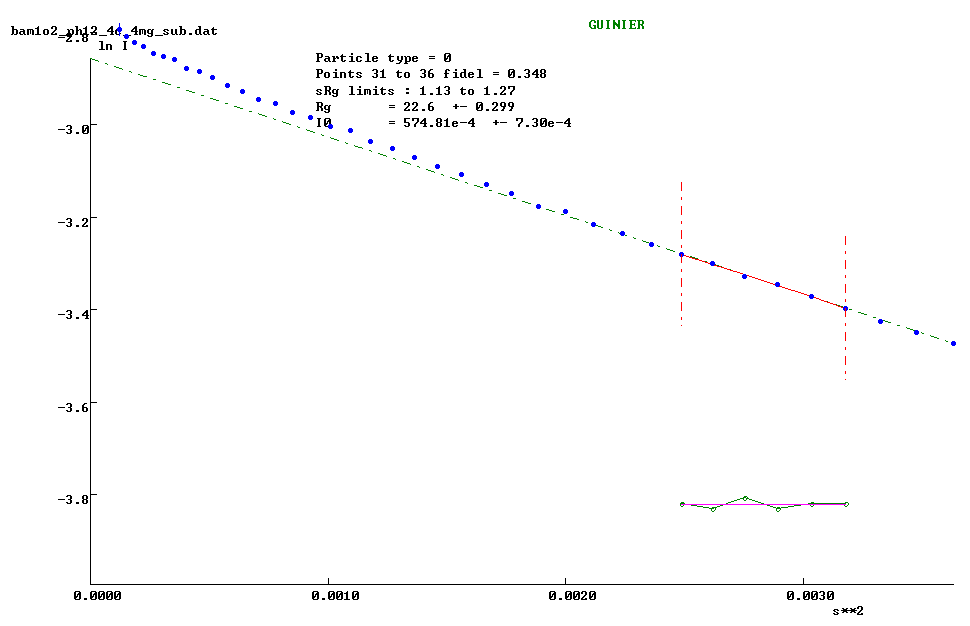 |
| --- | --- | --- |
| 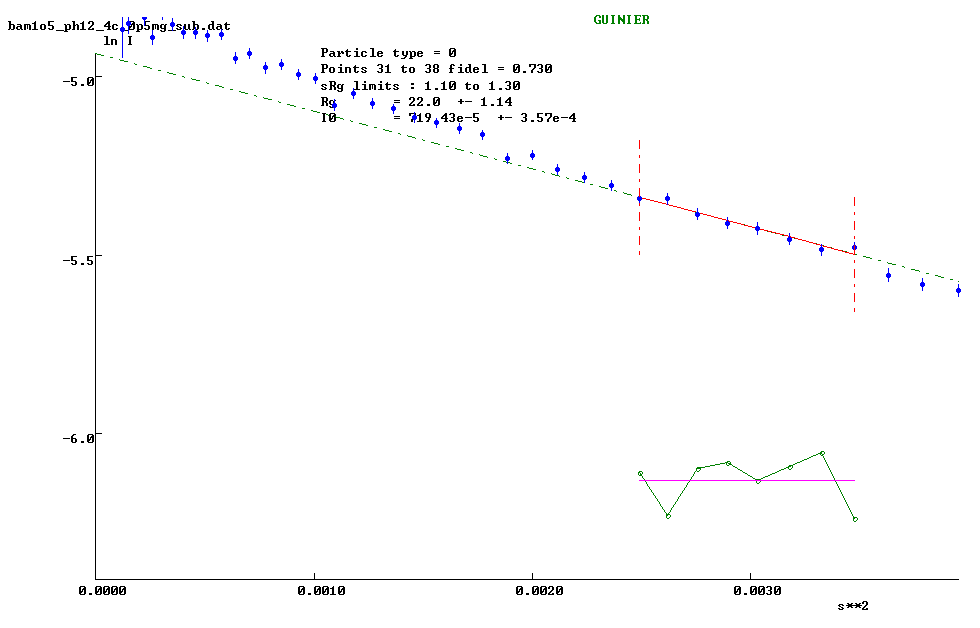(b) | 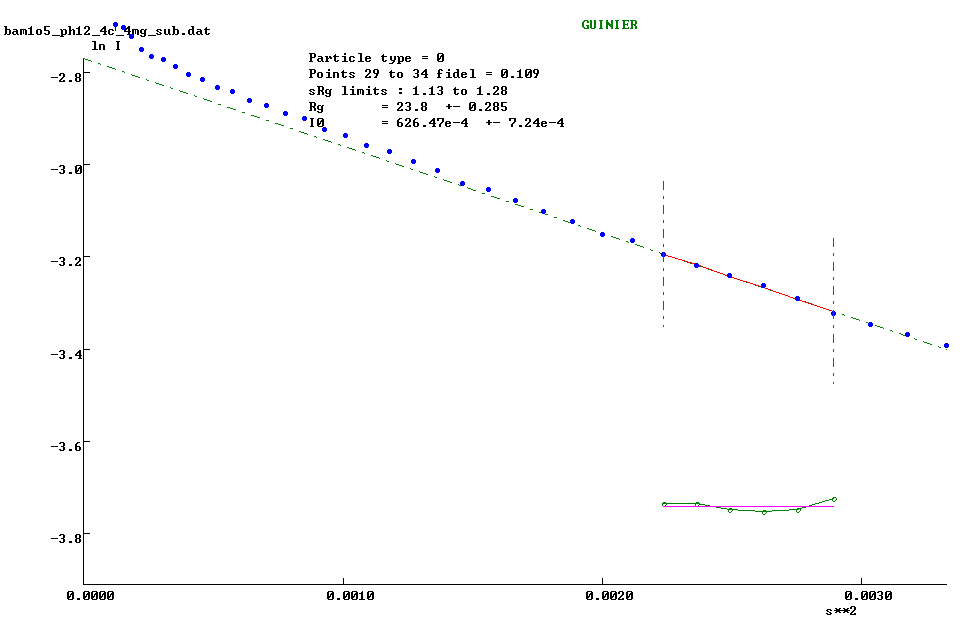 | 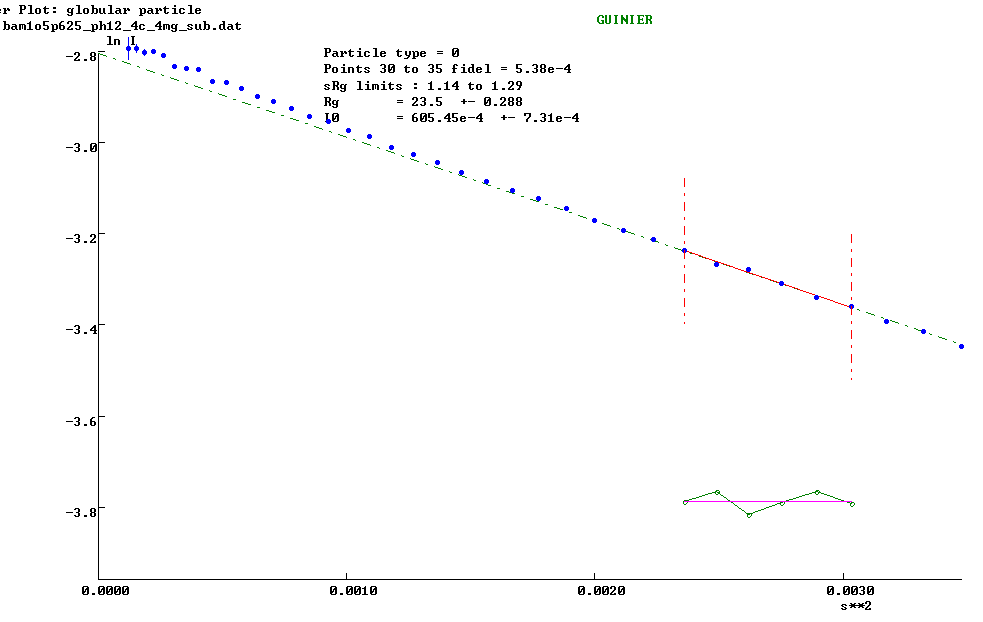 |
| 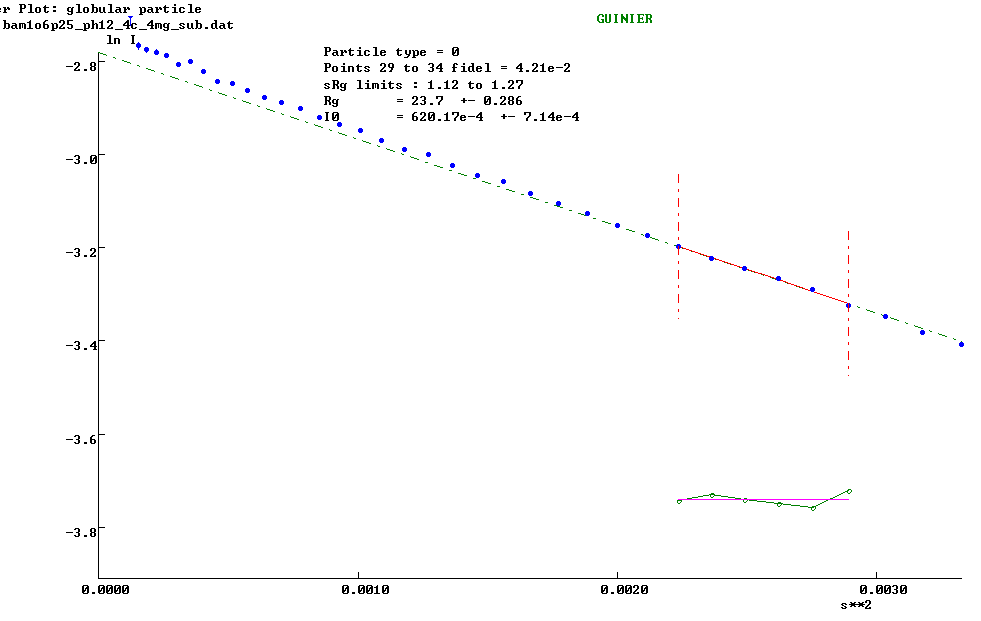(c) | 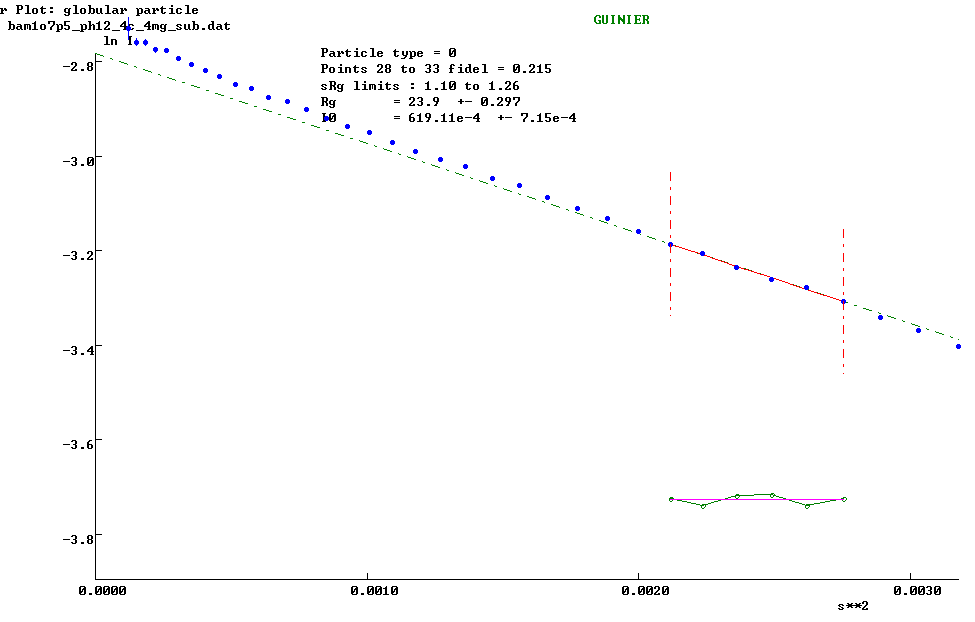 | 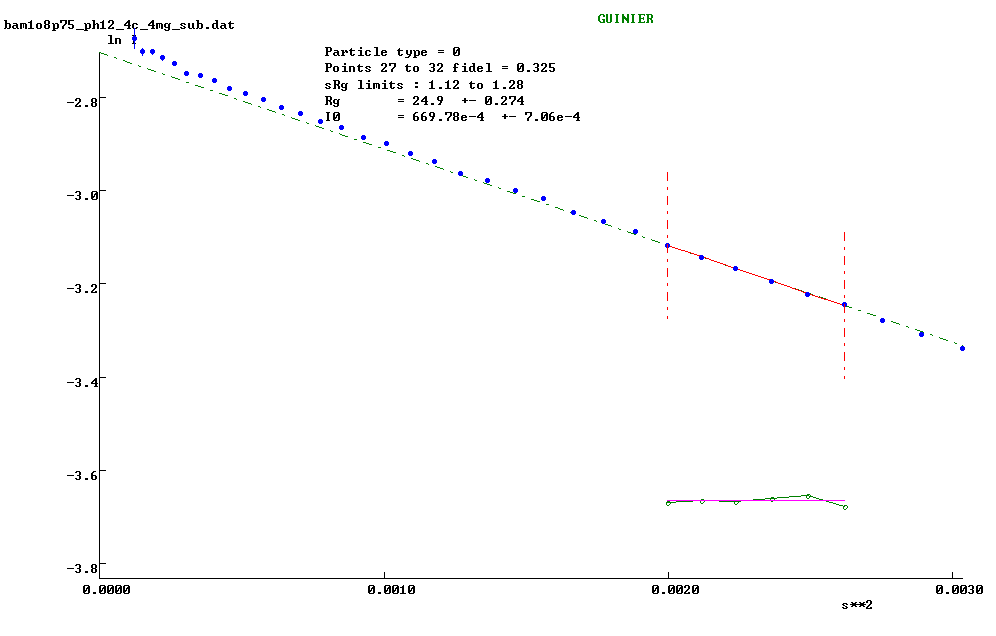 |
| 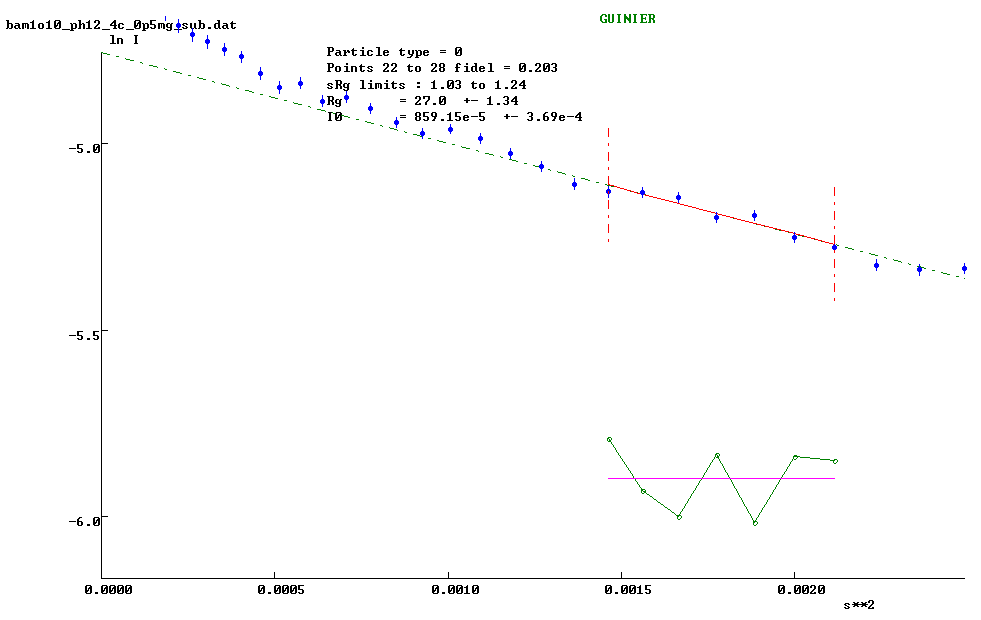(d) | 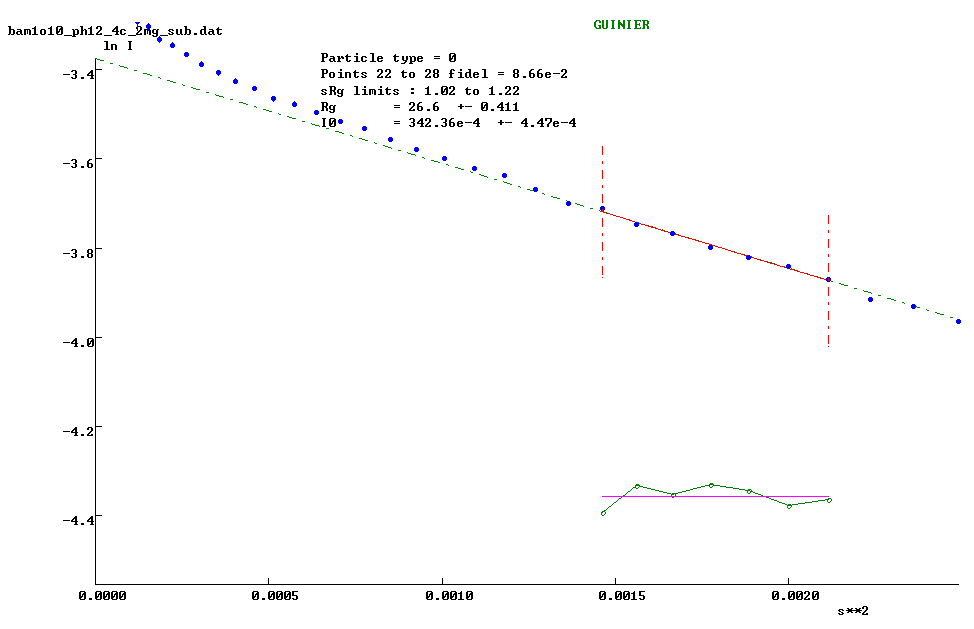 | 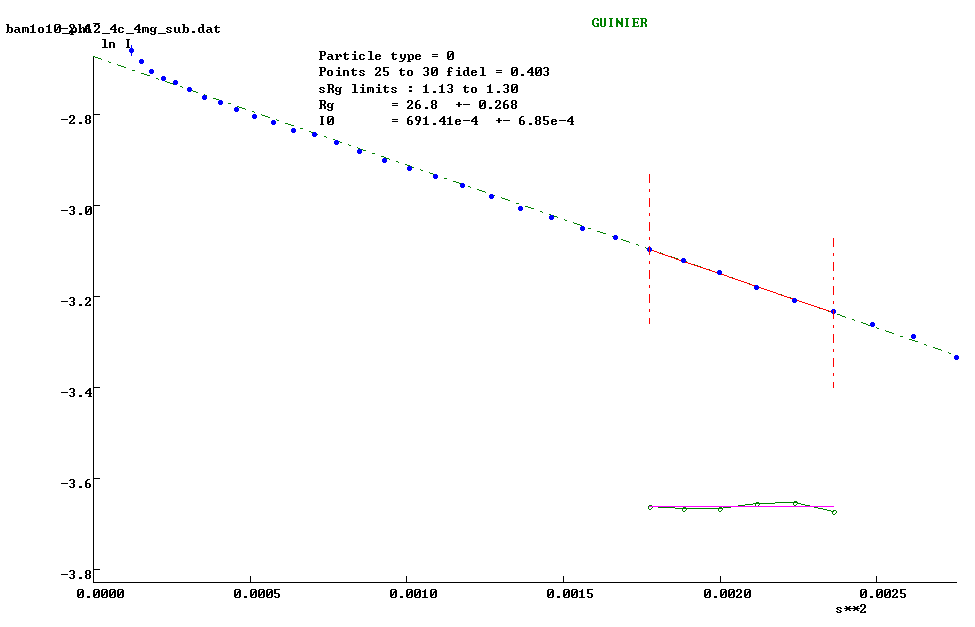 |
| 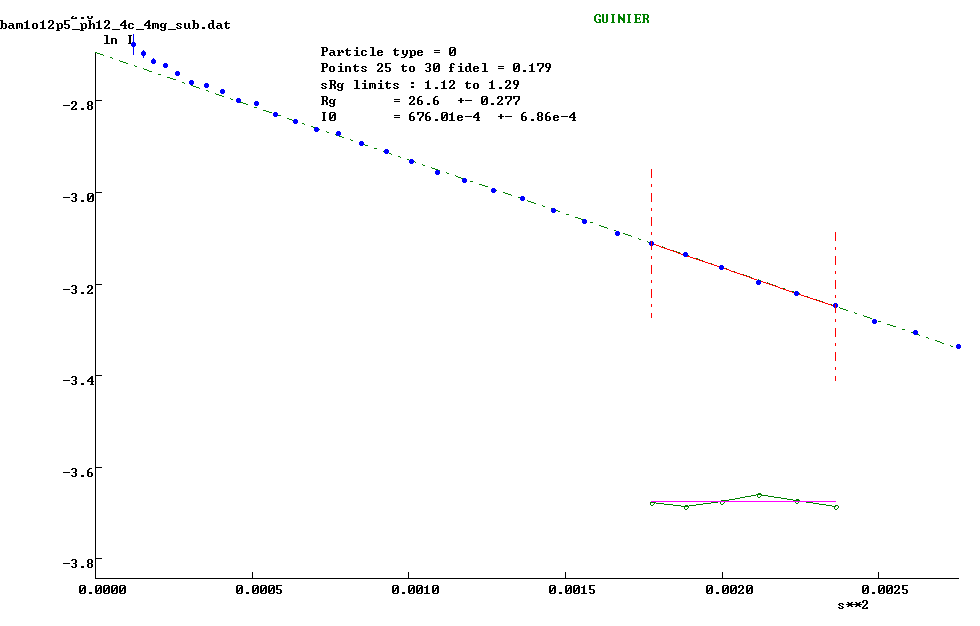(e) | 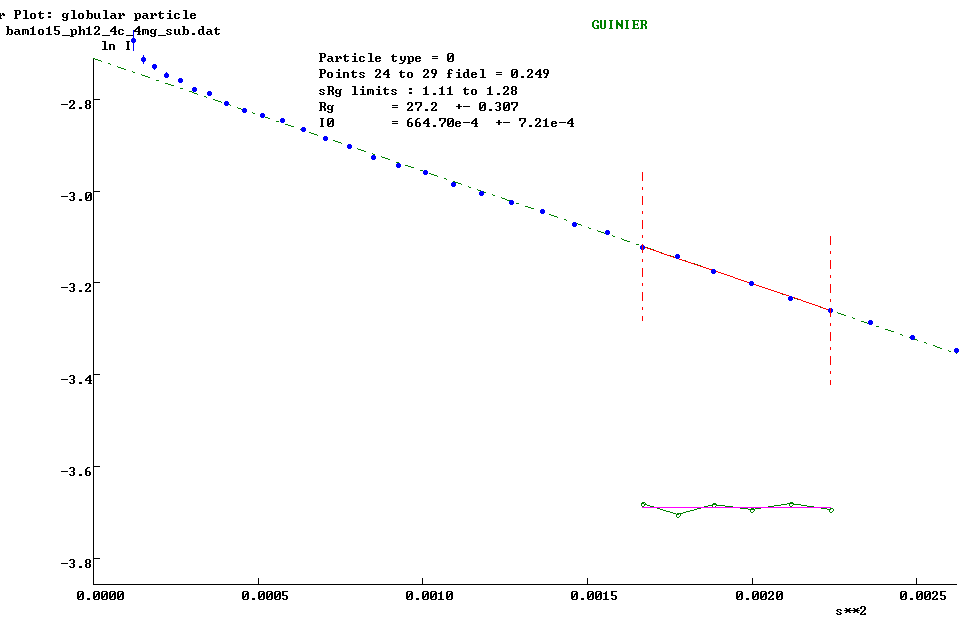 | 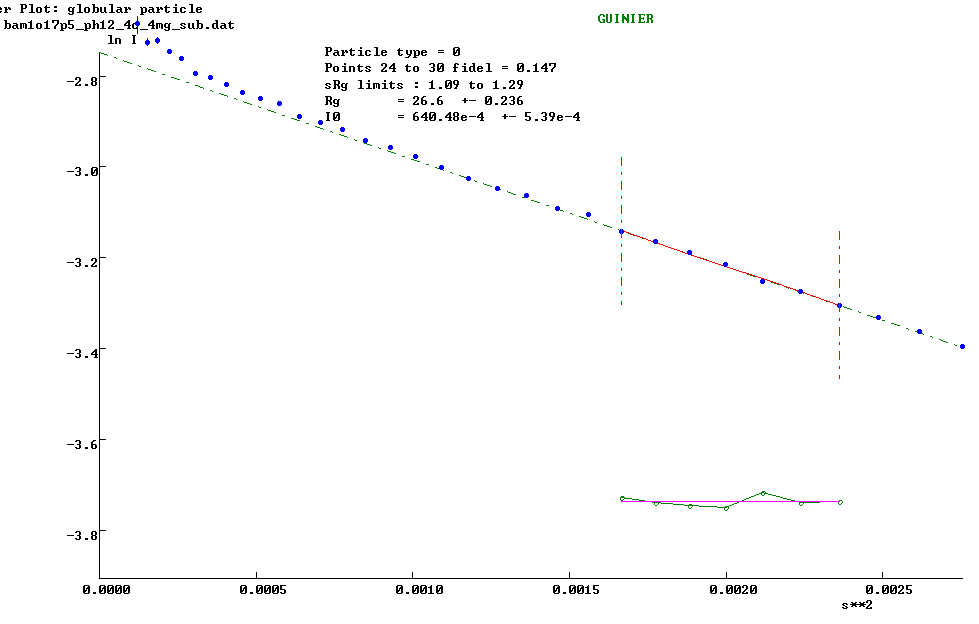 |
| 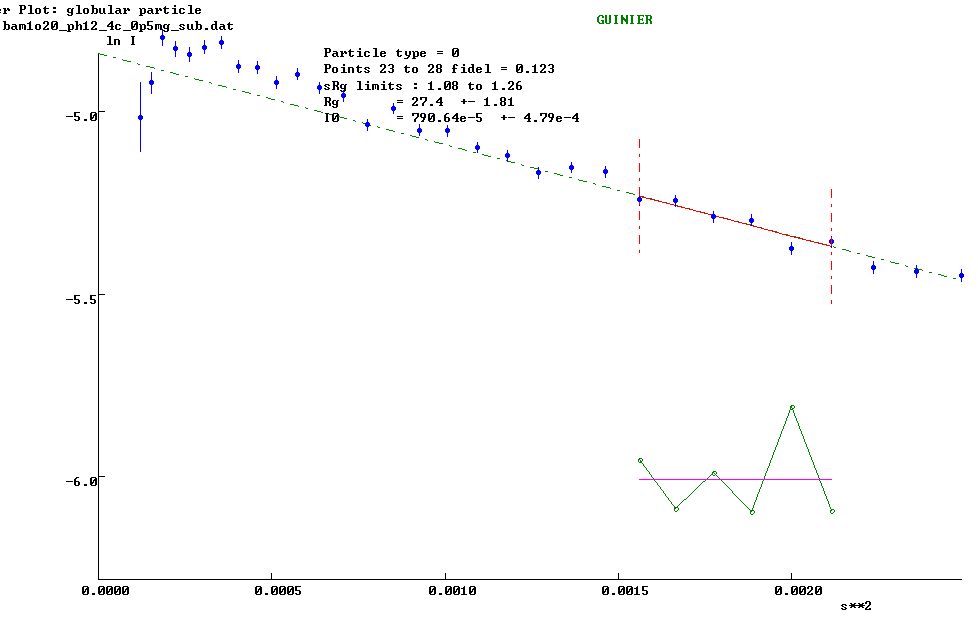(f) | 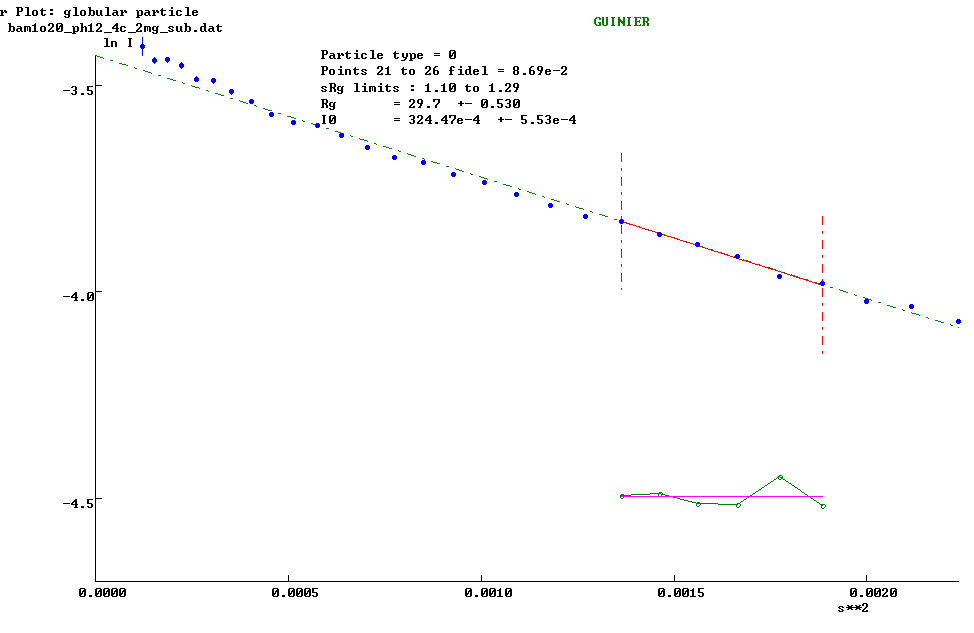 | 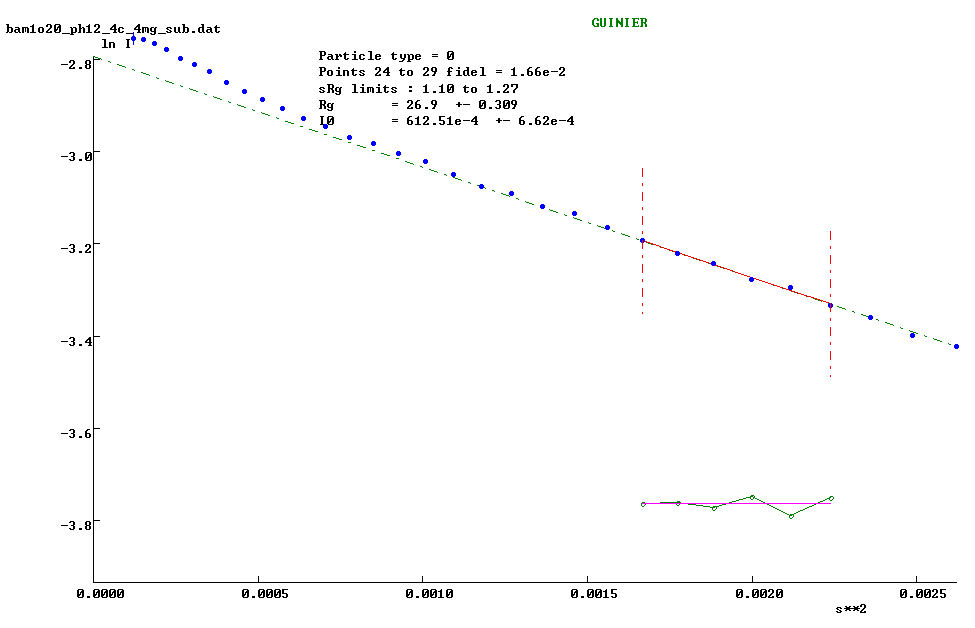 |
| 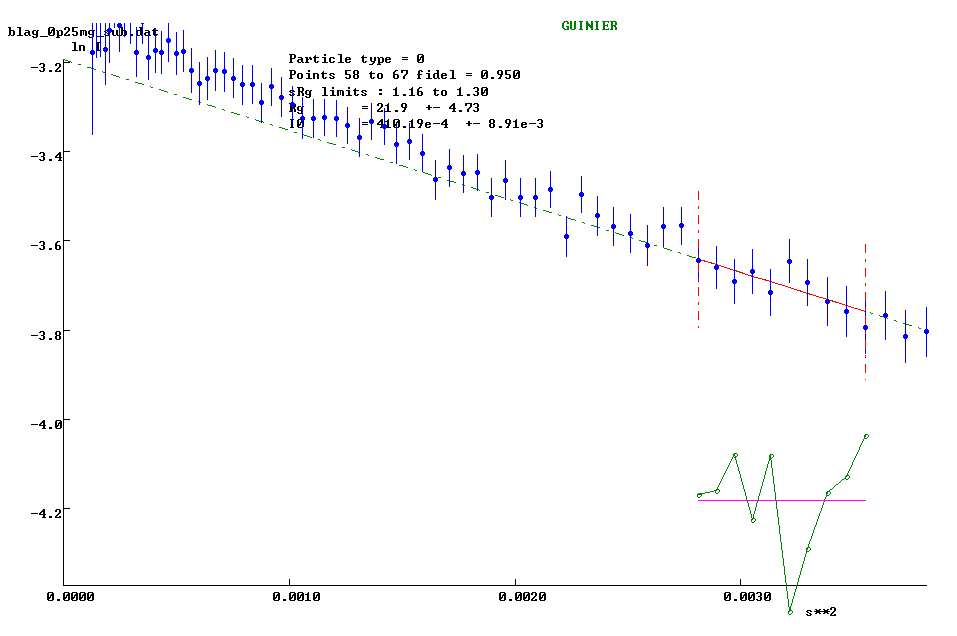 | 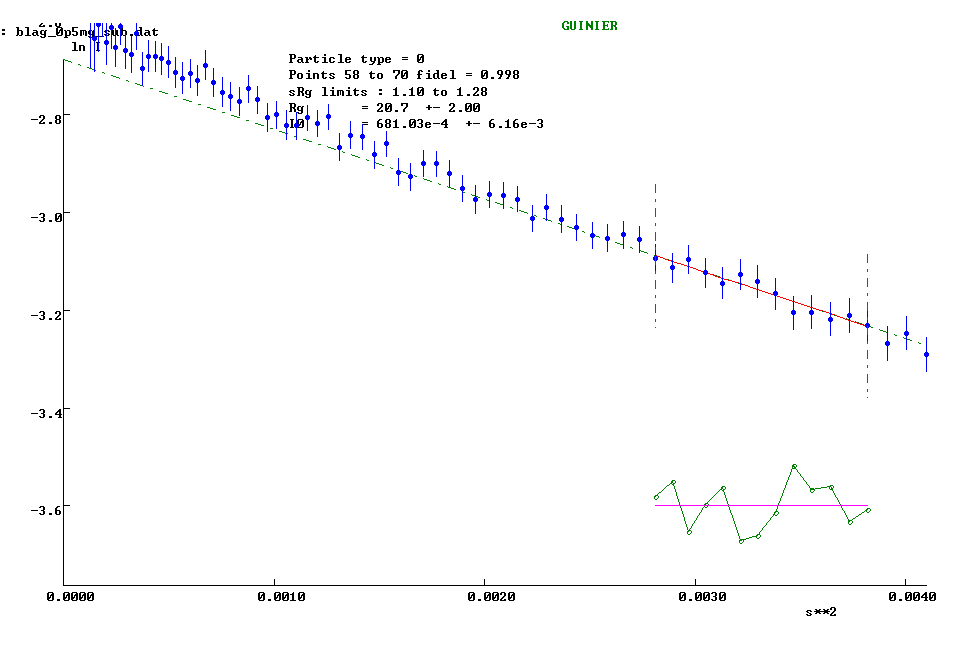 | 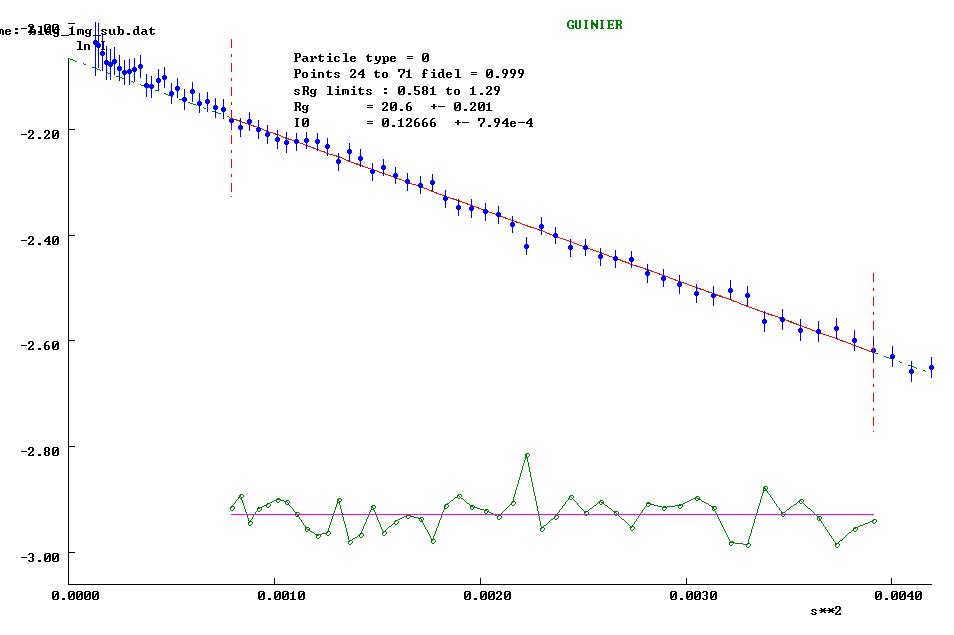 |
| 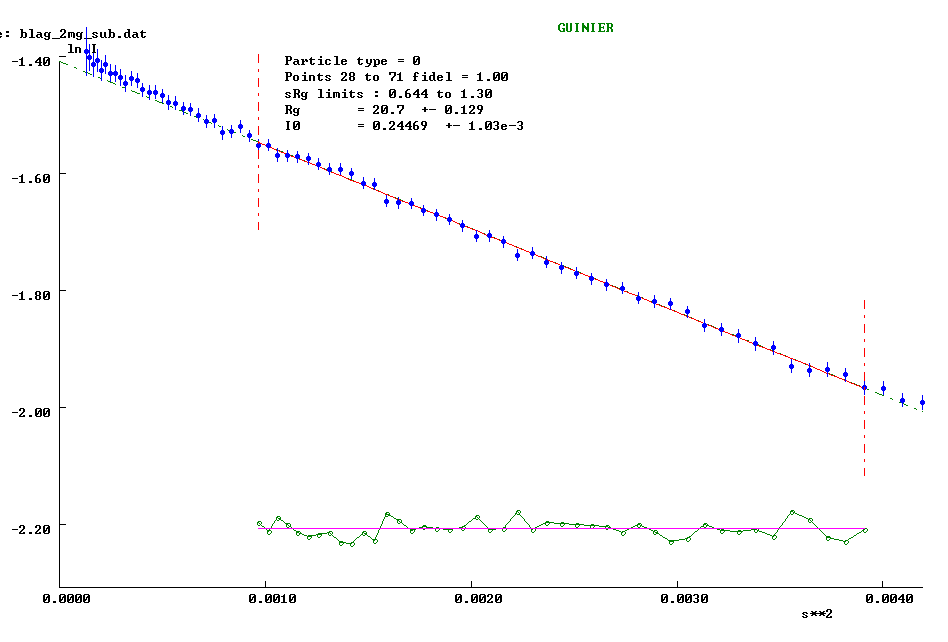(g) | 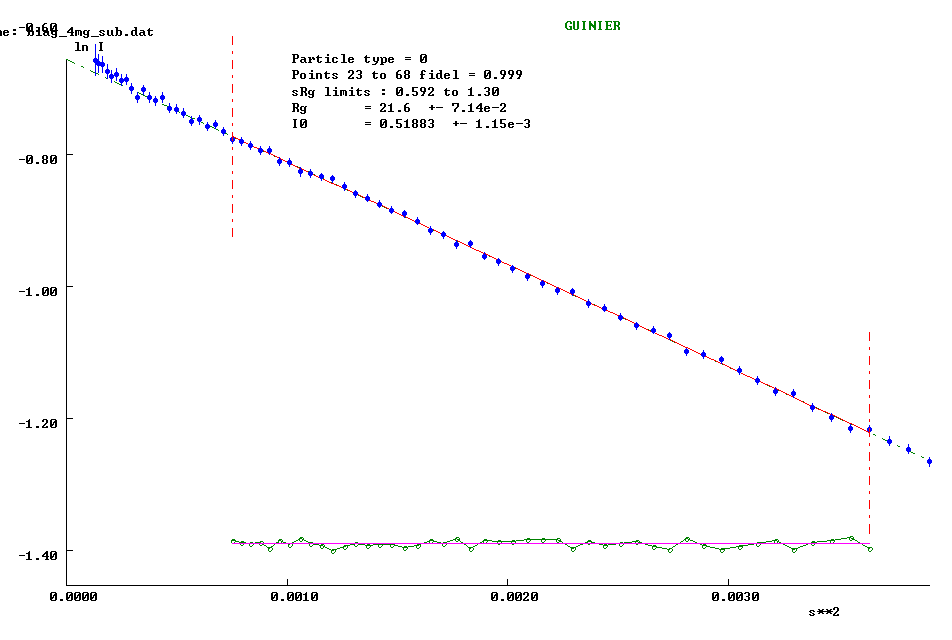 |  |
| 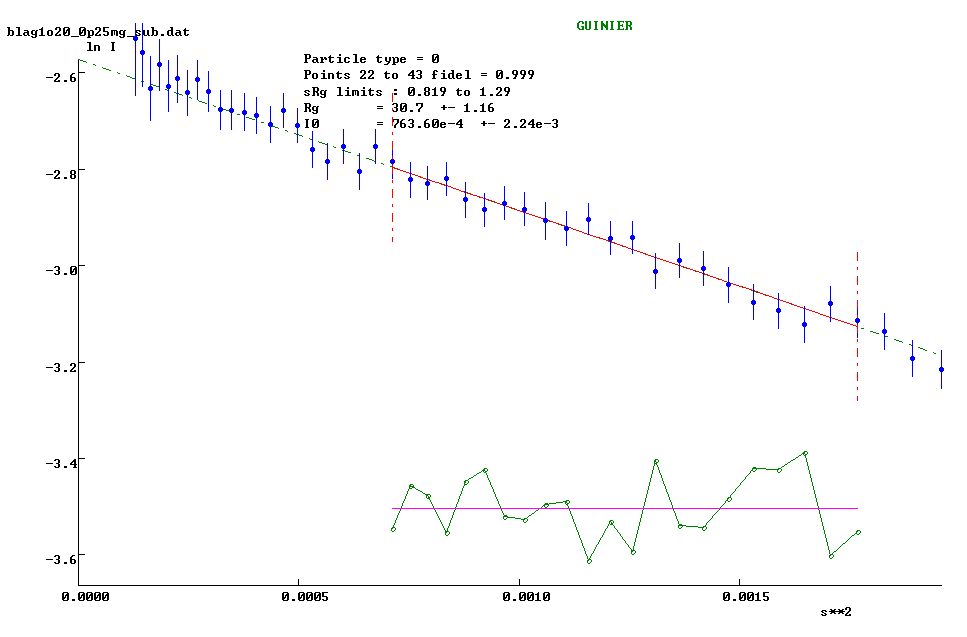 | 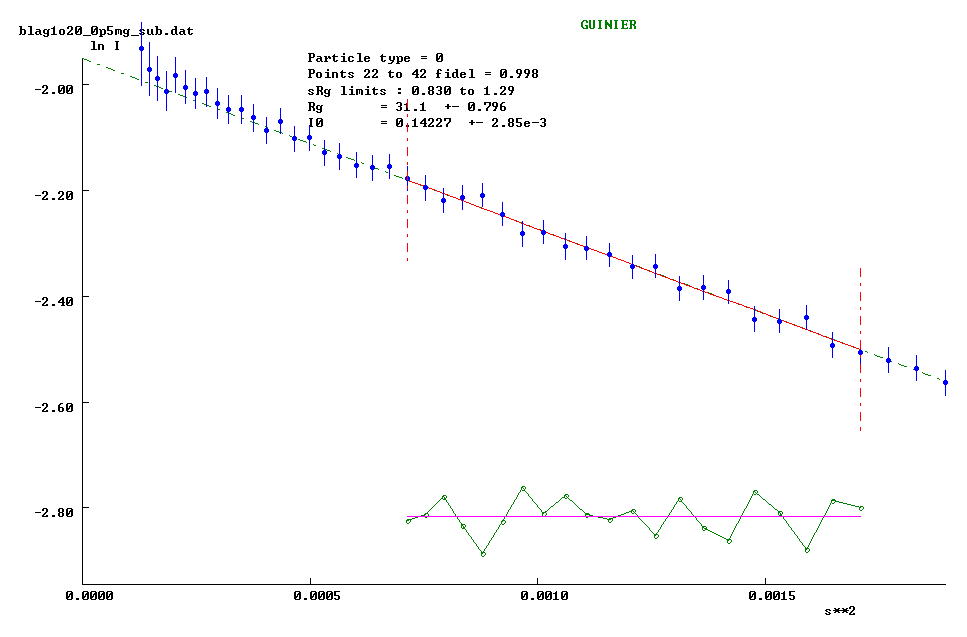 | 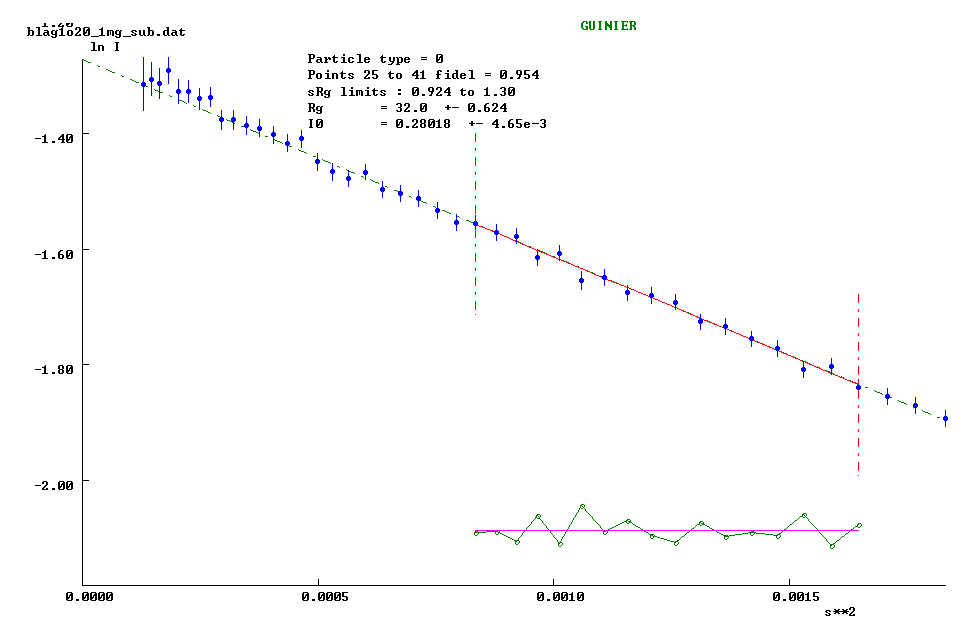 |
| 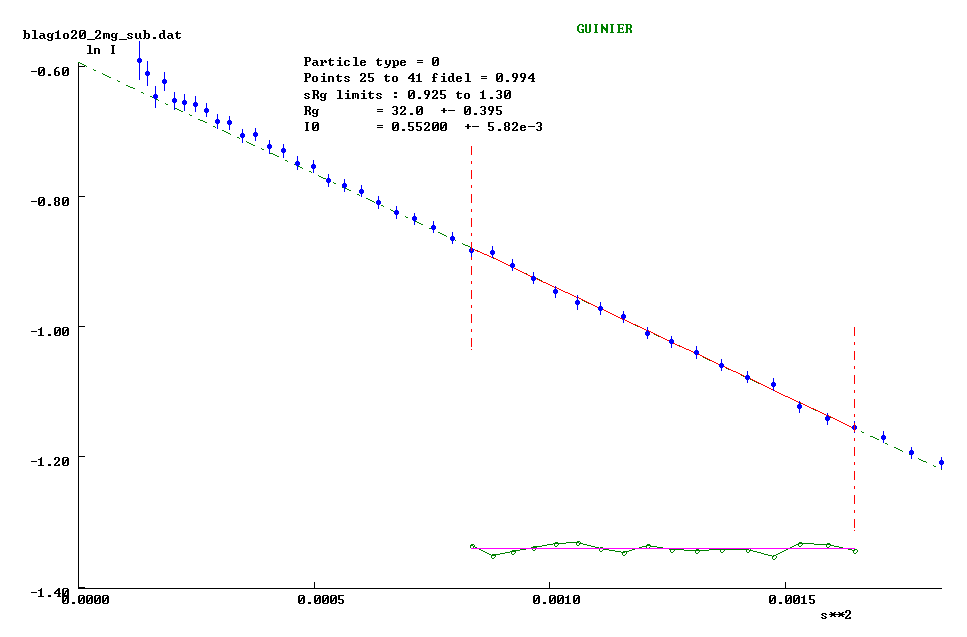(h) | 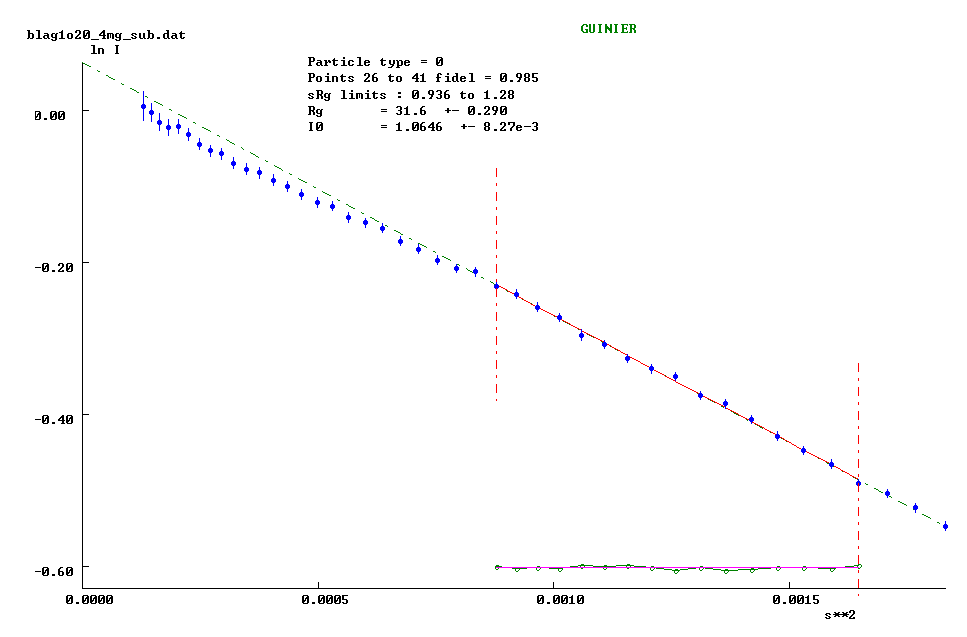 |  |
| 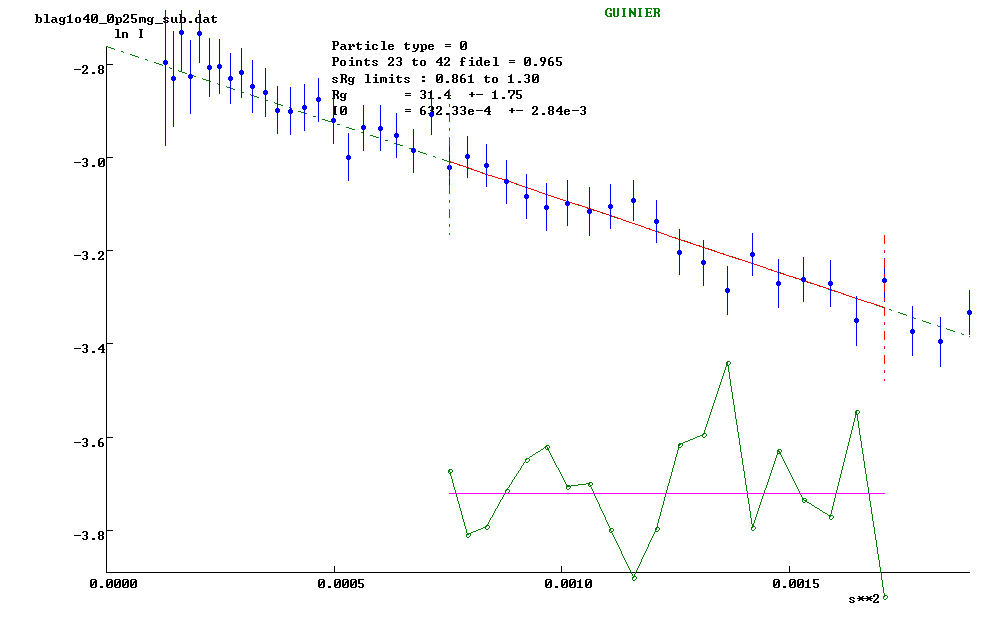 | 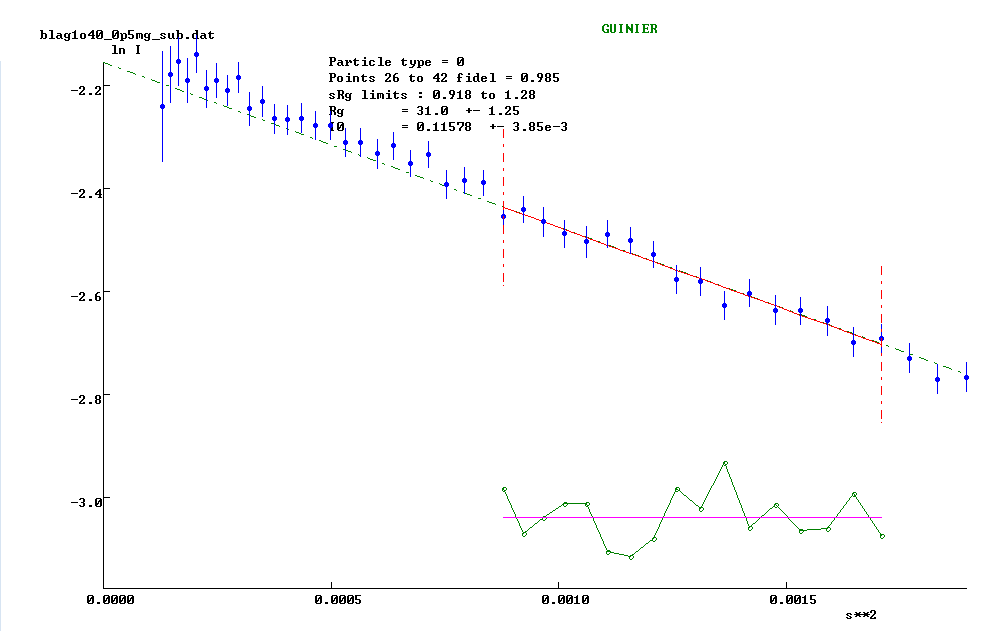 | 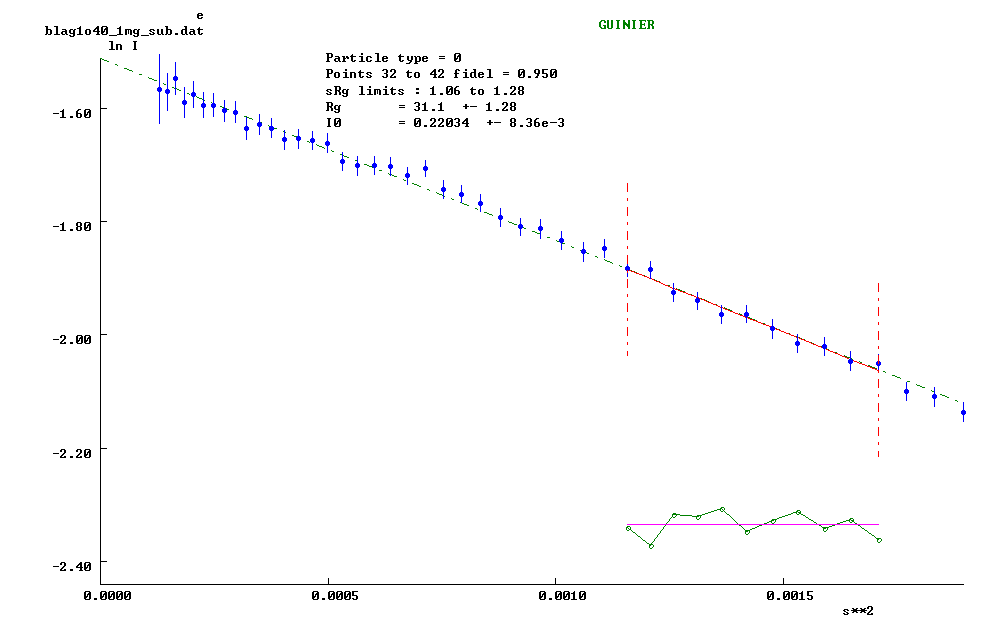 |
| 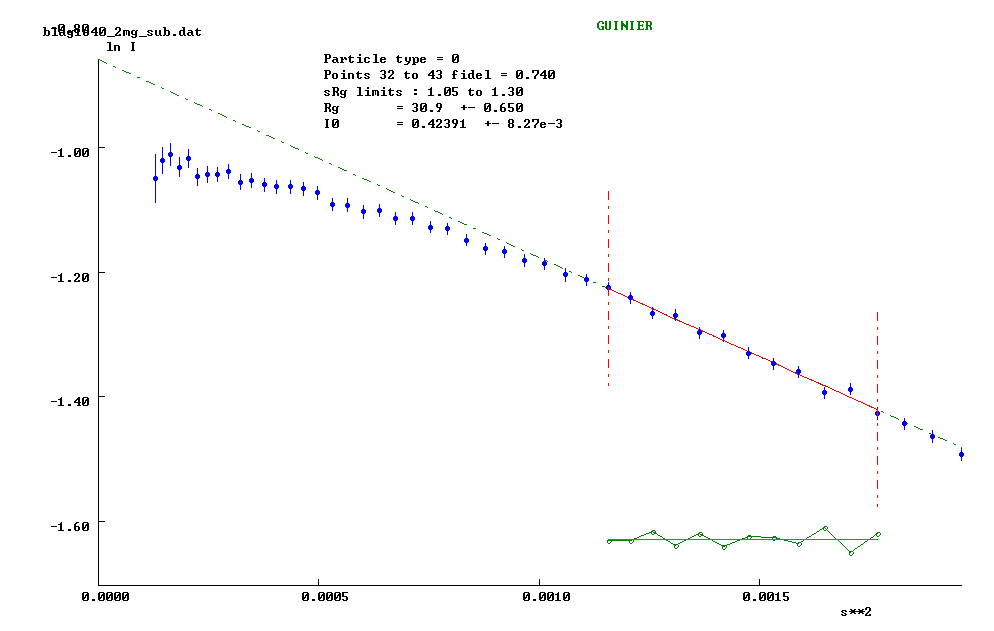  (i) | 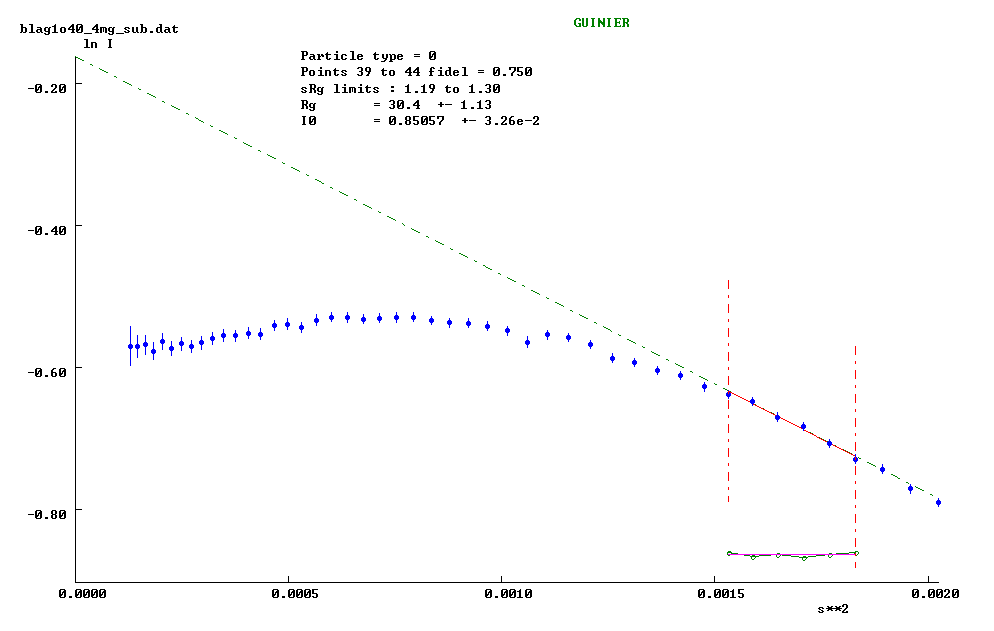 |  |
| 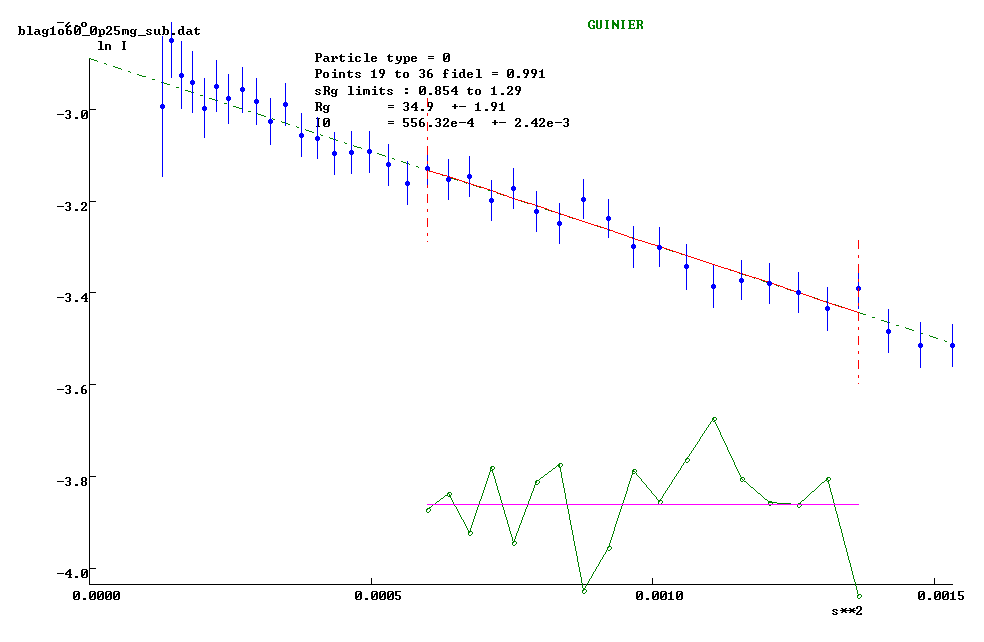 | 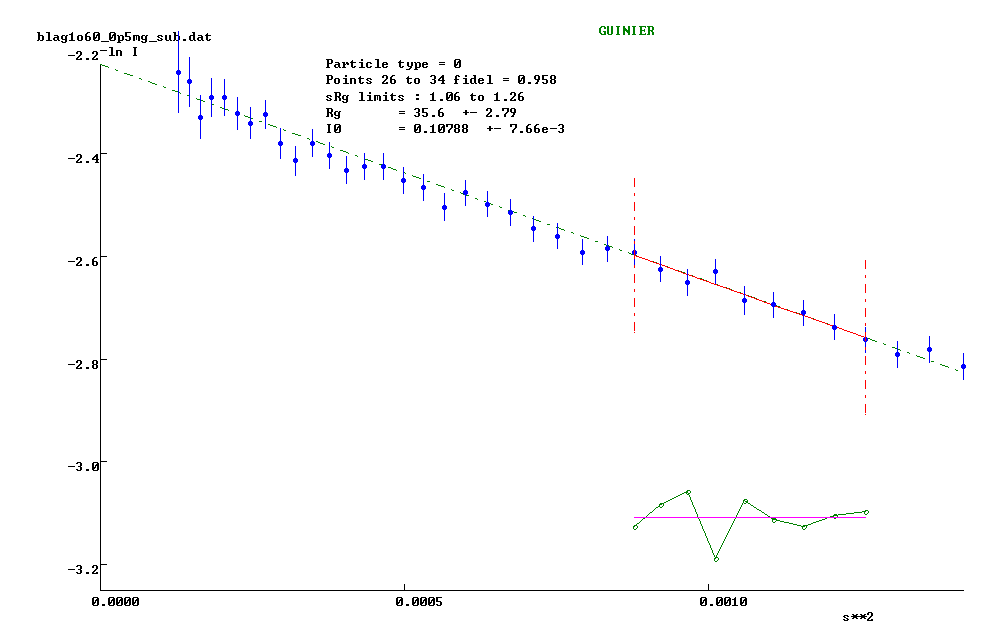 | 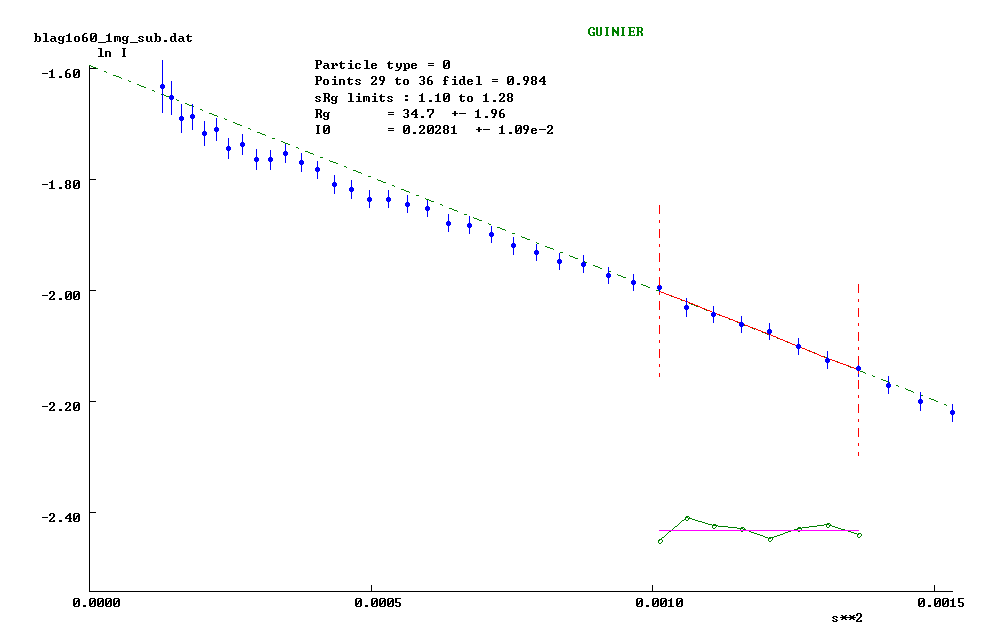 |
| 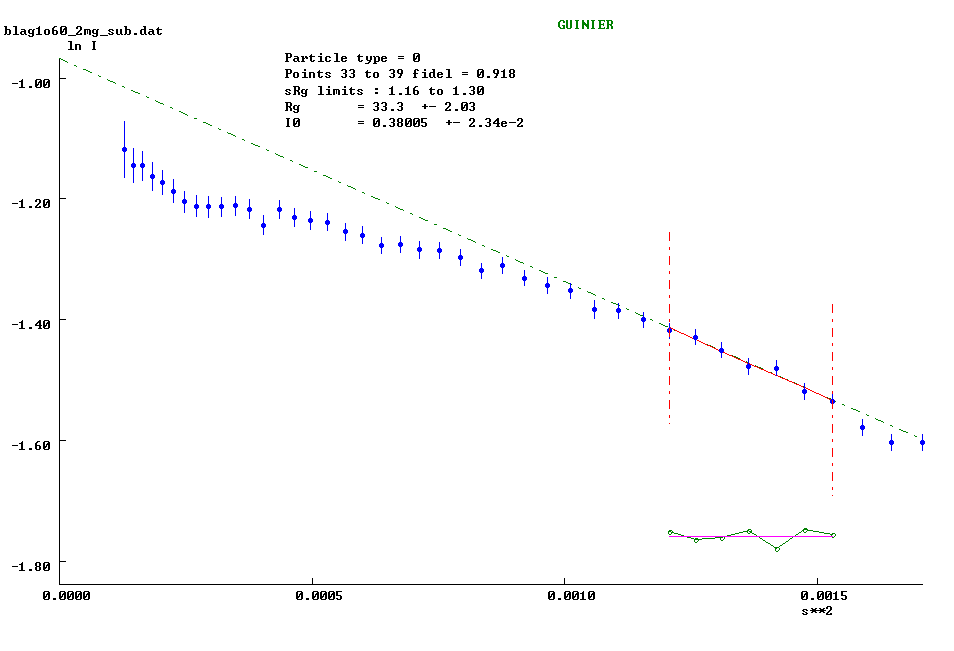(j) | 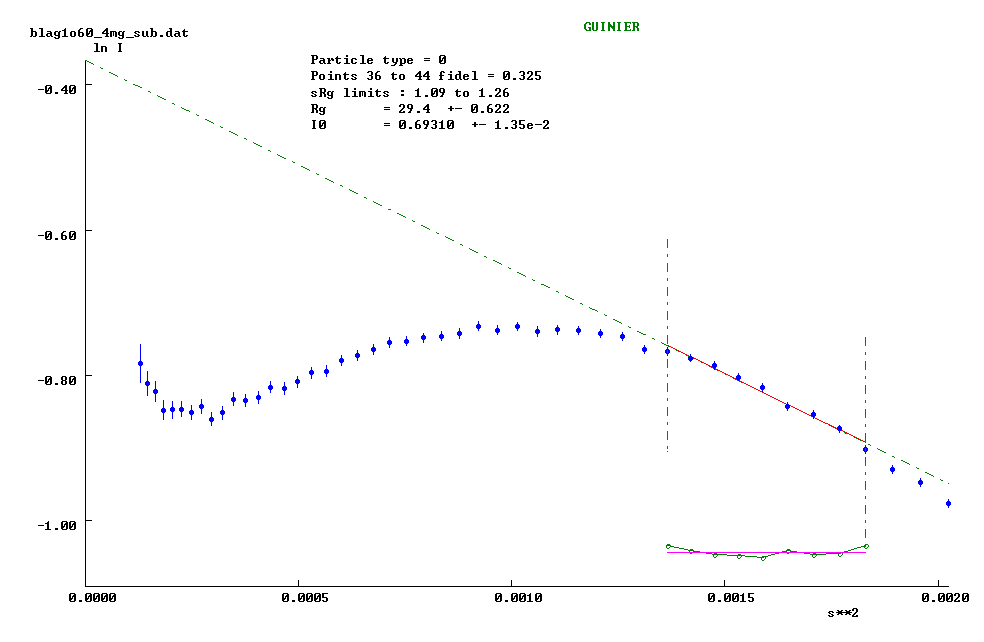 |  |
| 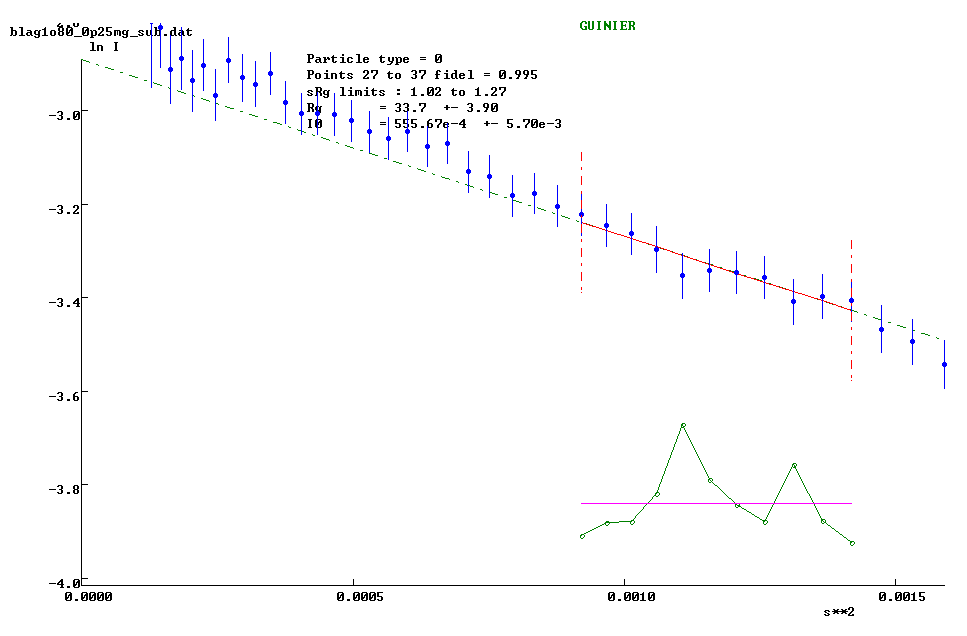 | 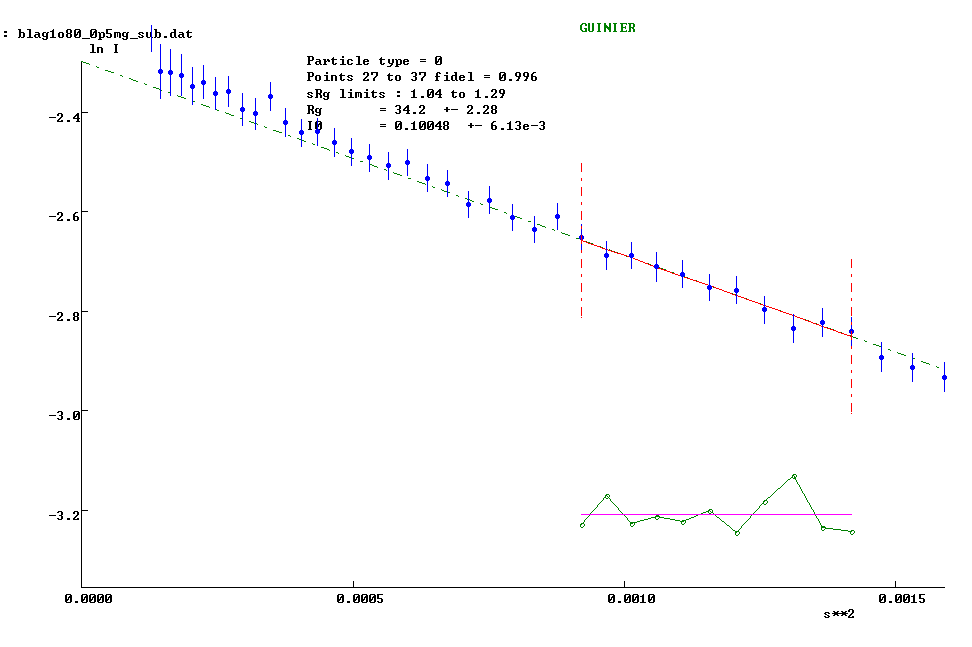 | 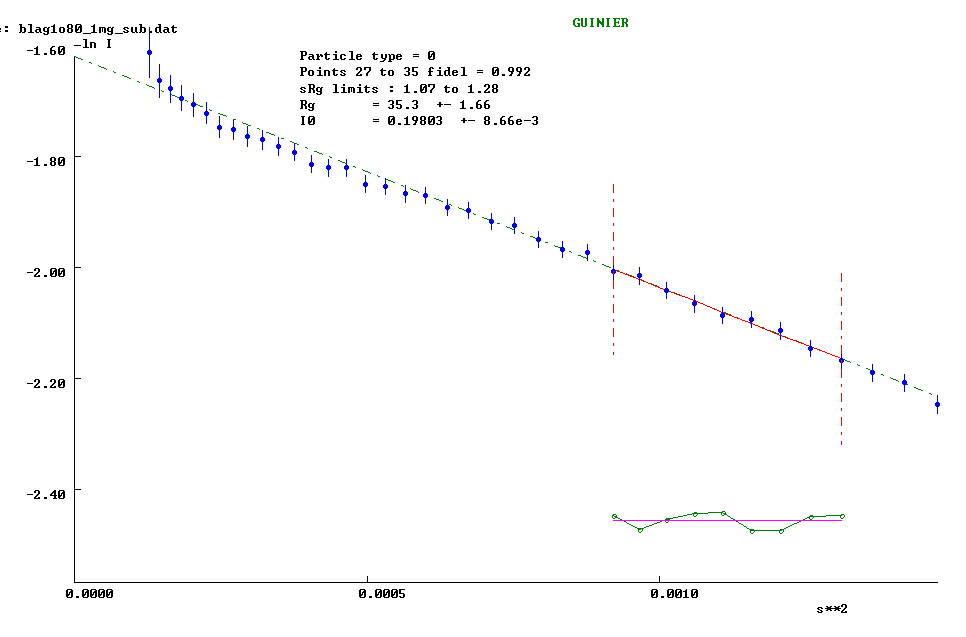 |
| 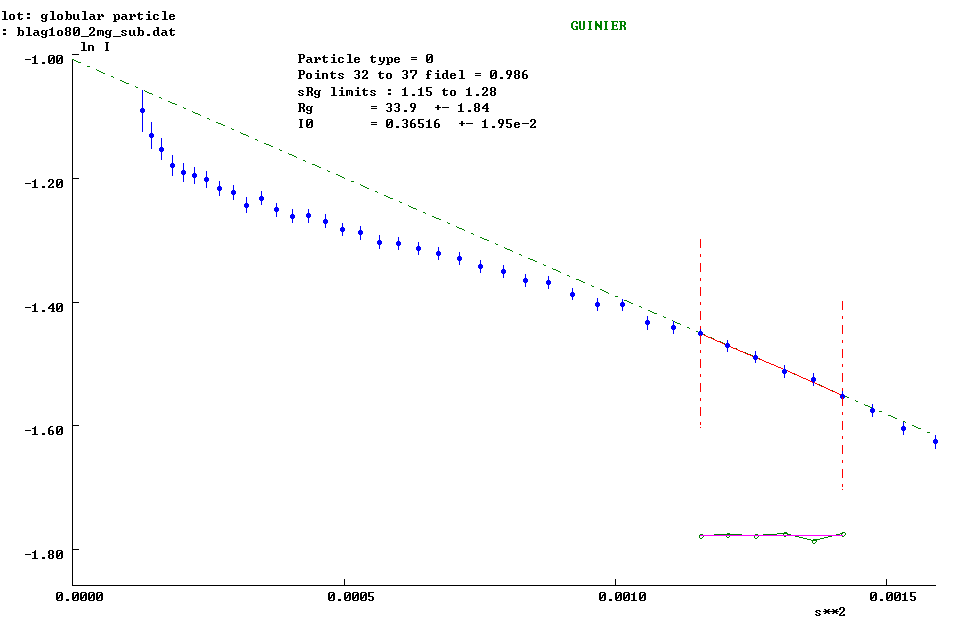(k) |  |  |

*Supplementary Figure H. Guinier plots (blue points) with linear fits (red dotted lines) and residuals (green lines) for the experimental scattering data for BAMLETs of protein:oleic acid ratios (a) 1:2, (b) 1:5, 1:5.625, (c) 1:6.25, 1:7.5, 1:8.75, (d) 1:10, (e) 1:12.5, 1:15, 1:17.5, (f) 1:20, at pH 12, 4°C, and (g) β-lactoglobulin and BLAGLETs of protein:oleic acid ratios (h) 1:20, (i) 1:40, (j) 1:60, (k) 1:80 at pH 12, 2% β-mercaptoethanol, 10°C. BAMLET species are 4 mg/ml or a concentration series of 0.5, 2, 4 mg/ml. BLAGLET species and β-lactoglobulin are a concentration series of 0.25, 0.5, 1, 2, 4 mg/ml. All measurements are on absolute scale.*

| 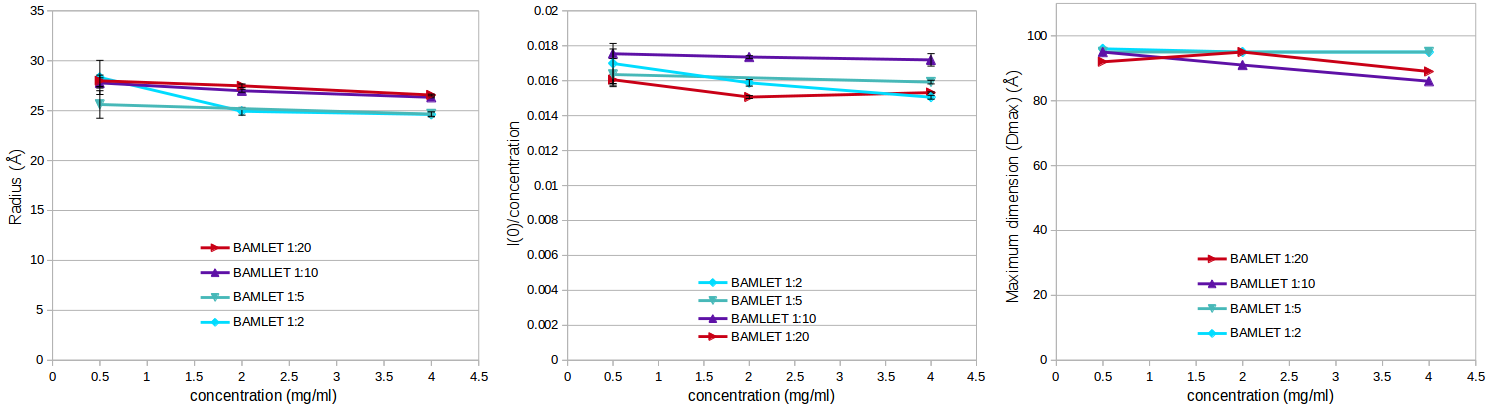(a) |
| --- |
| 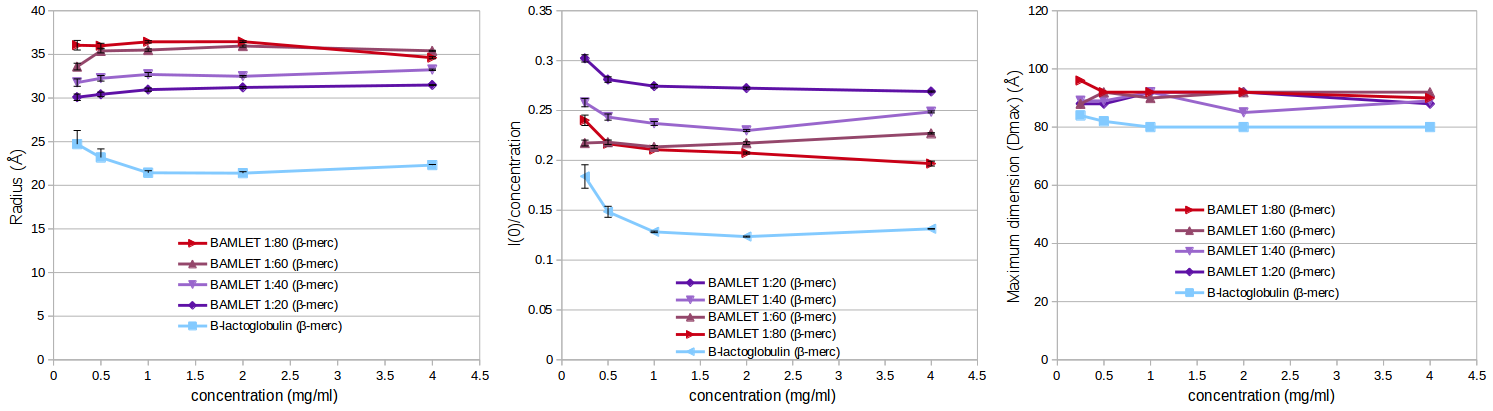(b) |
| 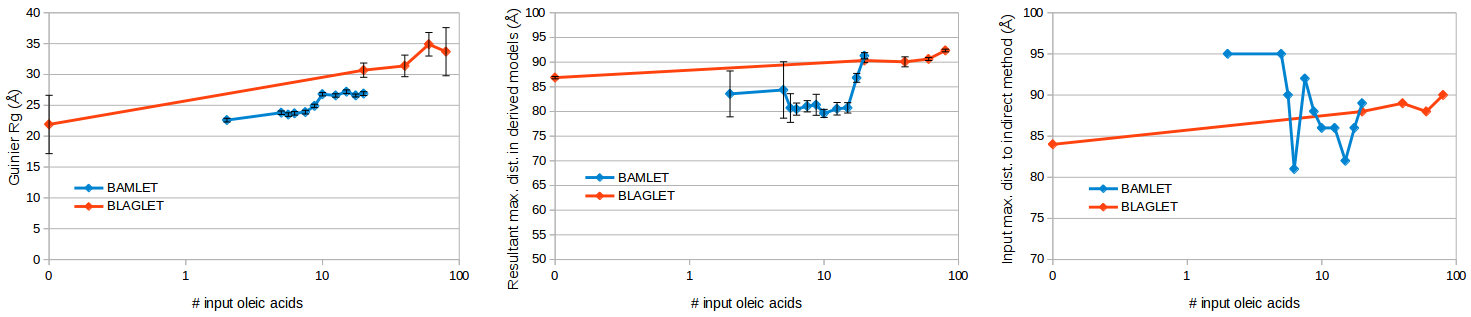(c) |

*Supplementary Figure I. Plots of SAXS-derived parameters vs. concentration: radius of gyration, R_g_, (left), intensity extrapolated to zero angle, I(0), (middle), and maximum molecular dimension, D_max_, (right) calculated by the Indirect Method (Glatter 1977) for (a) BAMLET (pH 12, 4°C) and (b) BLAGLET (pH 12, 2% β-mercaptoethanol, 10°C) species, showing that R_g_, I(0)/concentration, and D_max_ values remain constant over the concentration ranges measured (the plots are horizontal lines) indicating that these species are stable, mono-dispersed particles that are not subject to significant concentration-dependent aggregation or repulsion. (c) Plots of SAXS-derived parameters vs. input oleic acid content of species: radius of gyration, R_g_, (left, derived by the Guinier method (Guinier 1938)), resultant maximum dimension (middle, averaged from SAXS-derived models), and D_max_ (right, input to the Indirect Method to derive models), showing that the R_g_, which is associated with mass, increases for both BAMLET and BLAGLET as oleic acid content increases, whereas BAMLET maximum dimension decreases and BLAGLET maximum dimension remains at a constant size that is larger than the maximum dimension of β-lactoglobulin without oleic acid.*

| 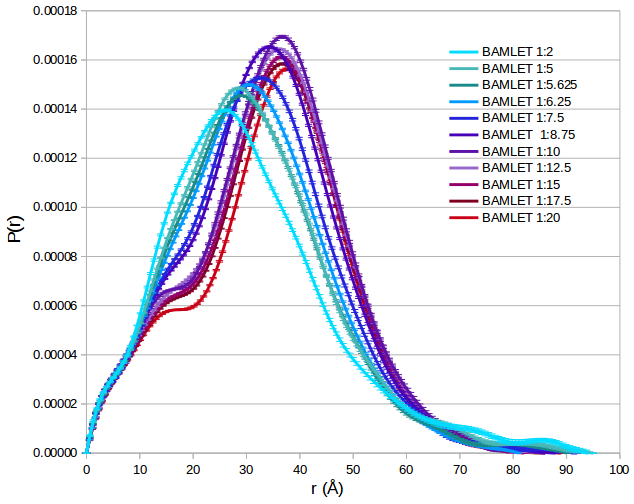(a) | 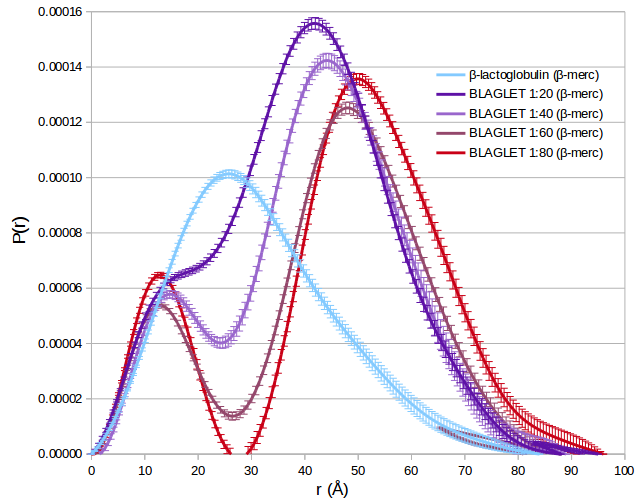(b) |
| --- | --- |

*Supplementary Figure J. Probability distribution profiles, P(r), for (a) BAMLET species in pH 12, 4°C and (b) BLAGLET species and β-lactoglobulin in pH 12, 2% β-mercaptoethanol, 10°C, derived from SAXS data by the Indirect Method (Glatter 1977) with the curves set to zero at zero and Dmax distances, used to create models of BAMLET and BLAGLET. The P(r) is a histogram of distances between scattering points (in this case, protein mass) in the complexes. Low and no oleic acid content BAMLETs and BLAGLETs have P(r) curves typical of globular proteins, with one maximum near the centre of the histogram. Medium and high oleic acid content BAMLET and BLAGLET P(r) curves have the shoulder that is characteristic of liprotides or the two maximums that is characteristic of liprotides and core-shell structure where the core does not significantly contribute to scattering (in this case, due to the similarity of its scattering length density with that of the aqueous solution).*

(a) BAMLET 1:2

| 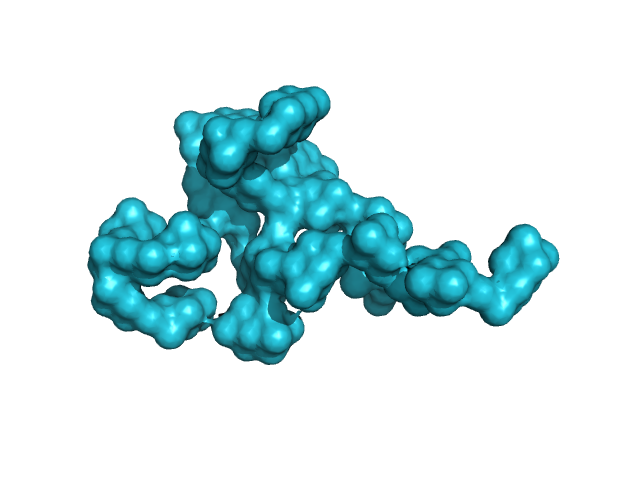 | 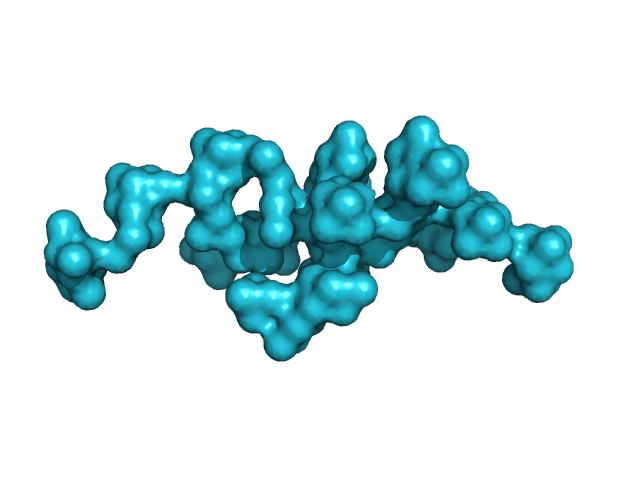 | 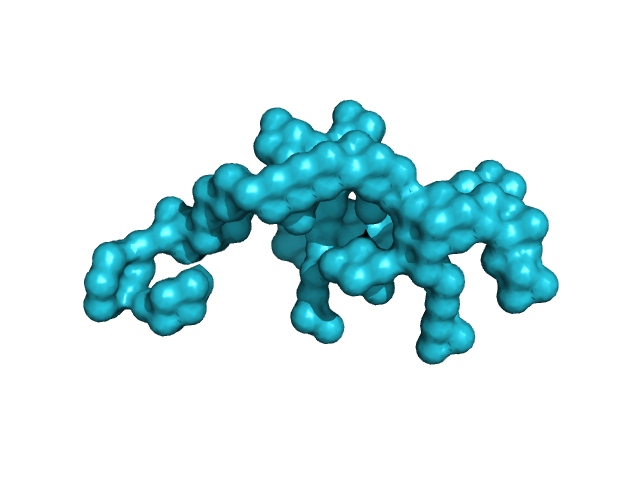 | 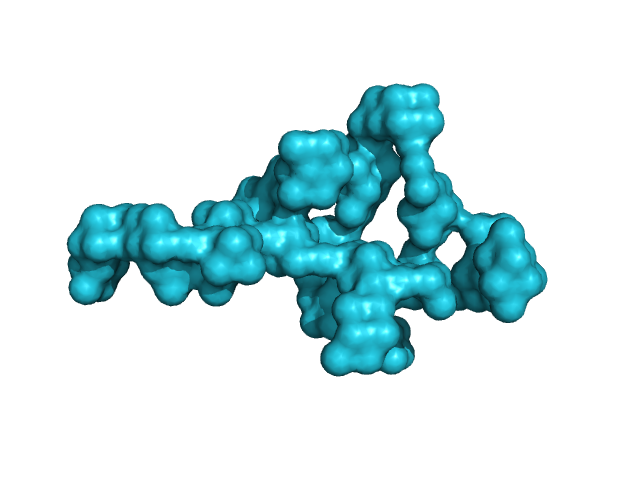 |
| --- | --- | --- | --- |
| 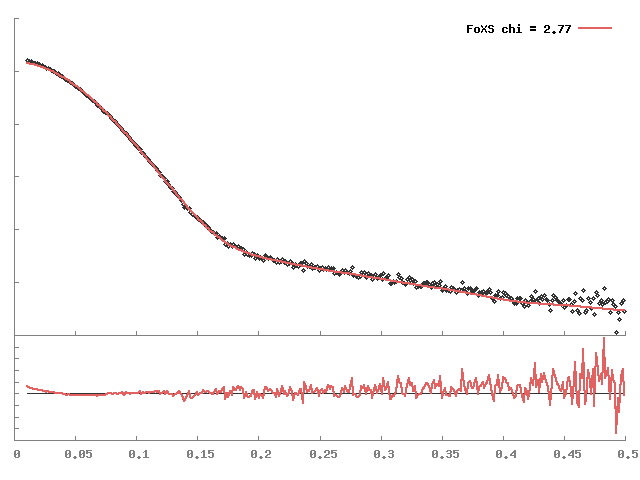 | 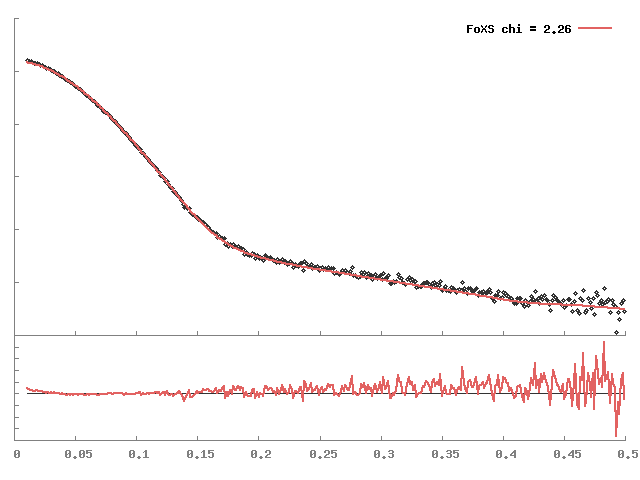 | 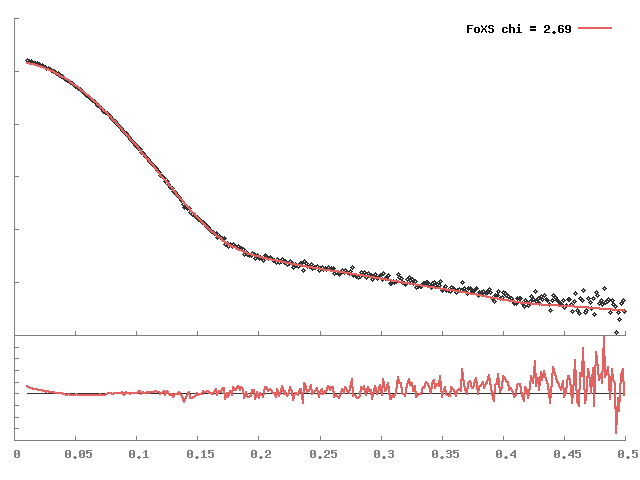 | 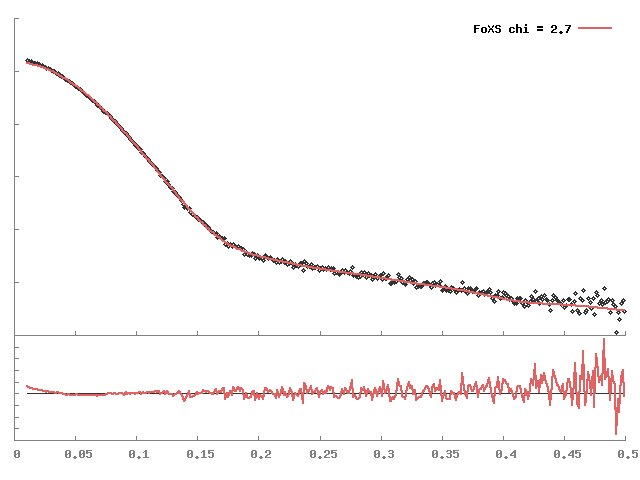 |

(b) BAMLET 1:5

| 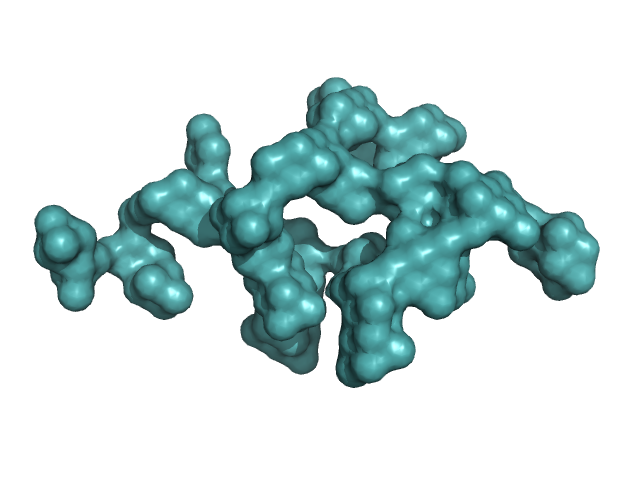 | 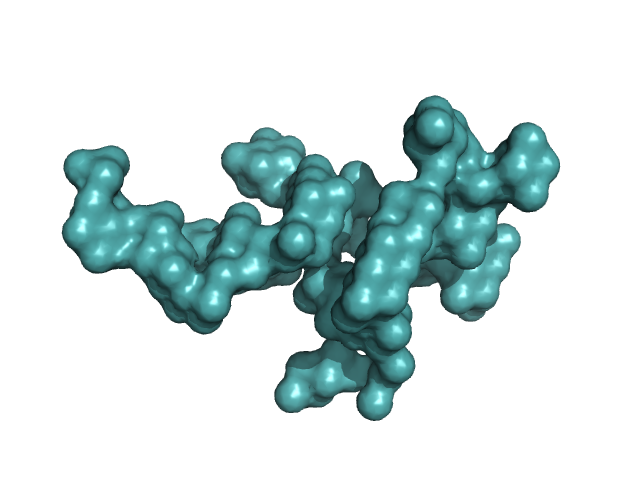 | 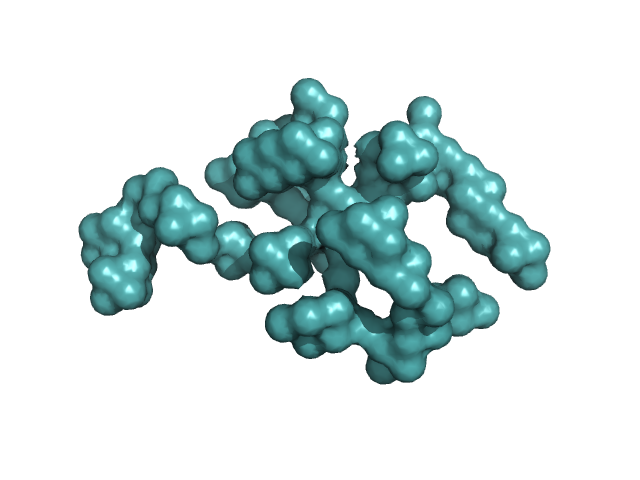 | 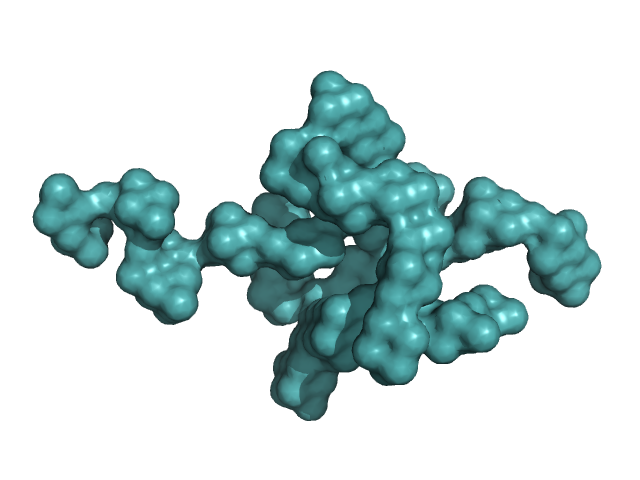 |
| --- | --- | --- | --- |
| 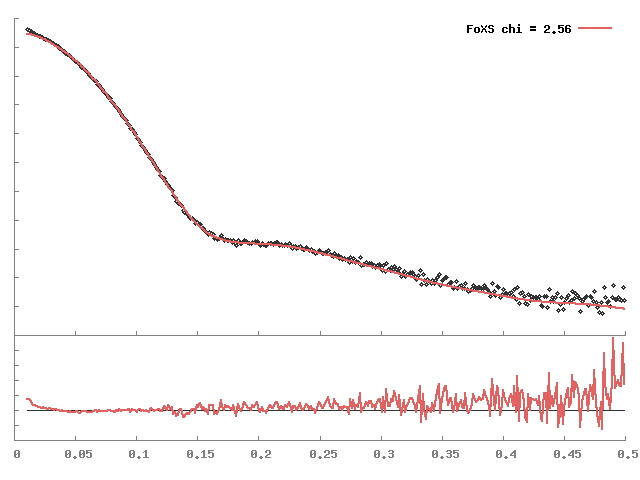 | 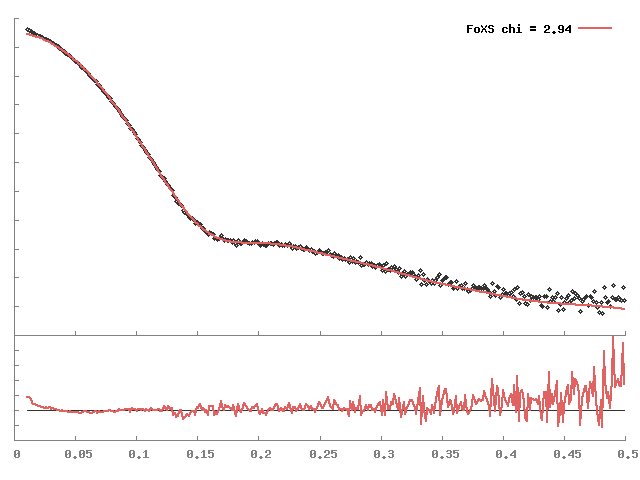 | 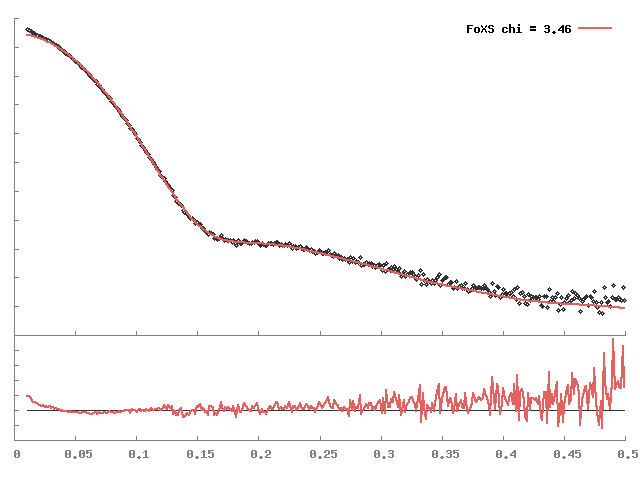 | 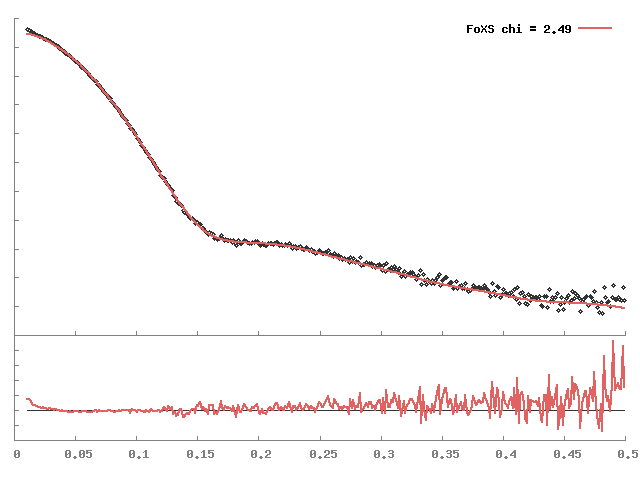 |

(c) BAMLET 1:5.625

| 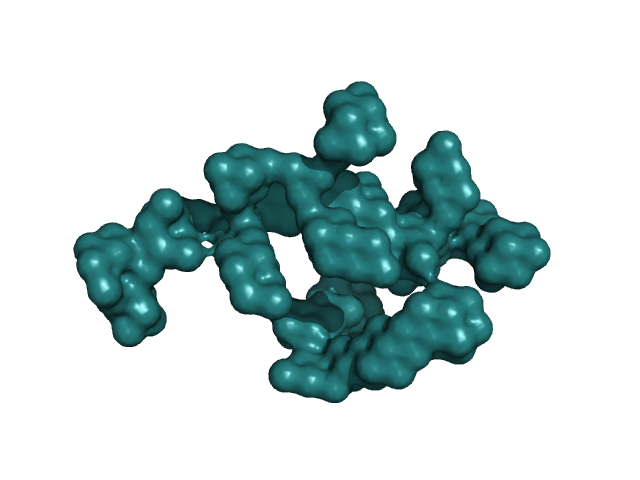 | 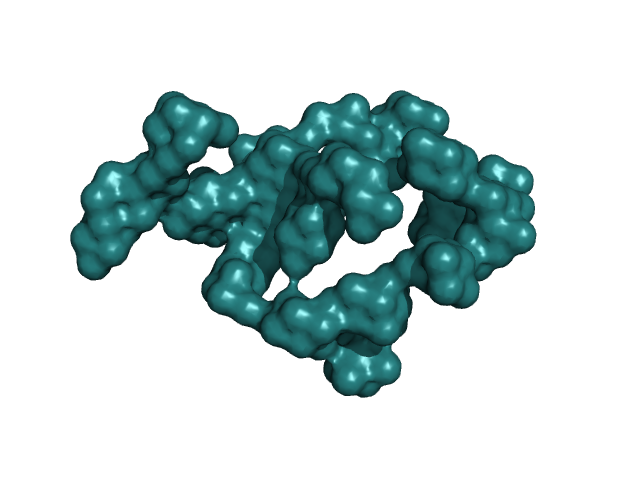 | 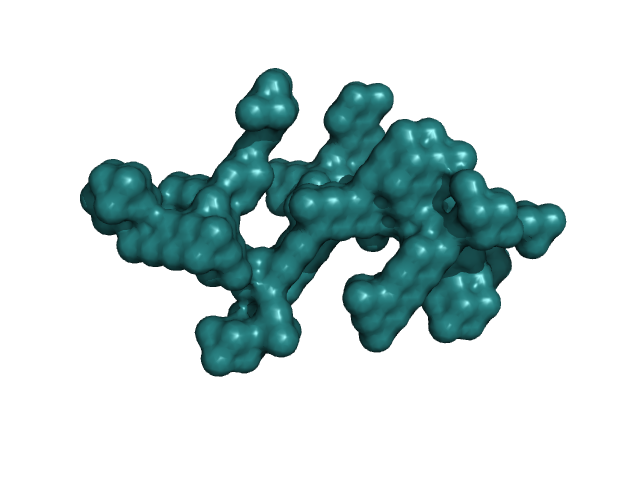 | 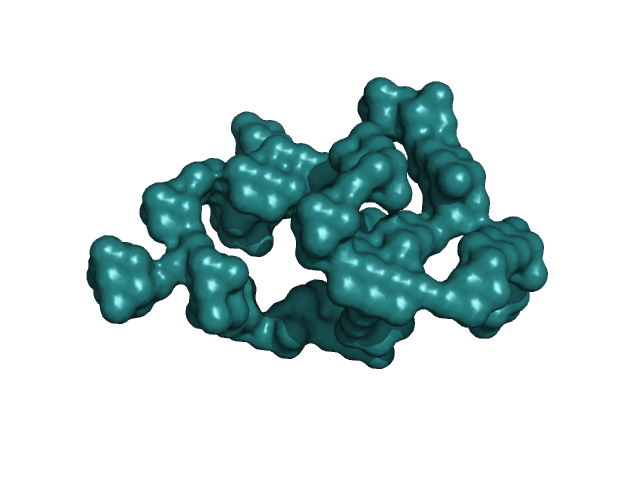 |
| --- | --- | --- | --- |
| 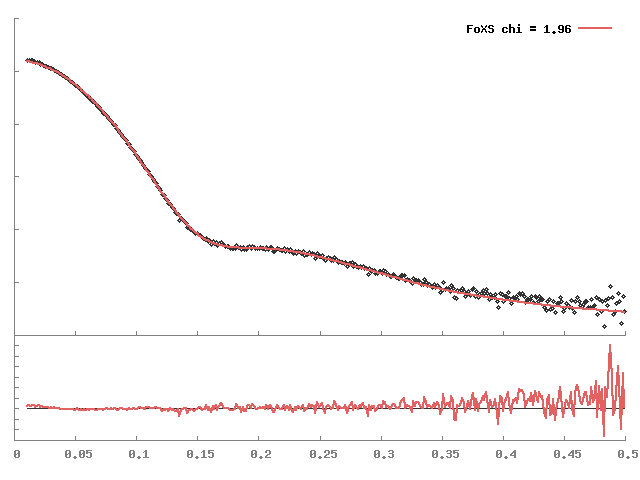 | 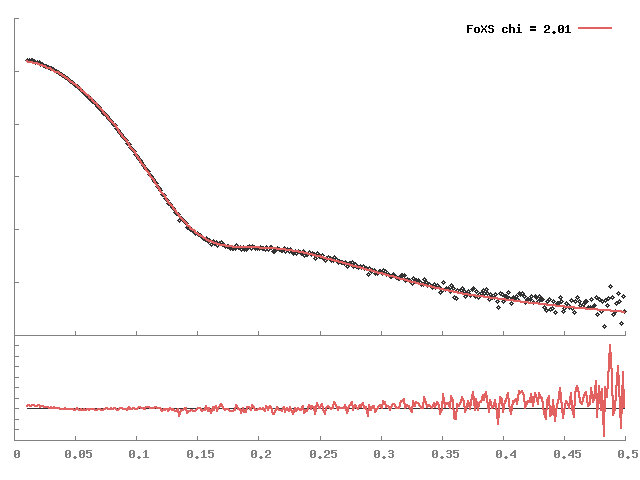 | 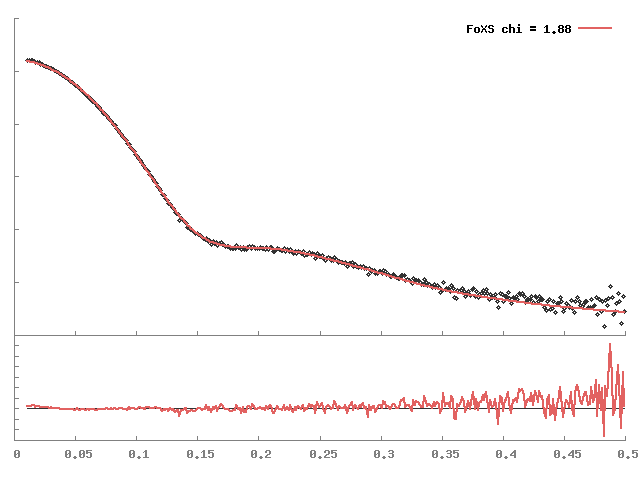 |  |

(d) BAMLET 1:6.25

|  |  |  |  |
| --- | --- | --- | --- |
|  |  |  |  |

(e) BAMLET 1:7.5

|  |  |  |  |
| --- | --- | --- | --- |
|  |  |  |  |

(f) BAMLET 1:8.75

|  |  |  |  |
| --- | --- | --- | --- |
|  |  |  |  |

(g) BAMLET 1:10

|  |  |  |  |
| --- | --- | --- | --- |
|  |  |  |  |

(h) BAMLET 1:12.5

|  |  |  |  |
| --- | --- | --- | --- |
|  |  |  |  |

(i) BAMLET 1:15

|  |  |  |  |
| --- | --- | --- | --- |
|  |  |  |  |

(j) BAMLET 1:17.5

|  |  |  |  |
| --- | --- | --- | --- |
|  |  |  |  |

(k) BAMLET 1:20

|  |  |  |  |
| --- | --- | --- | --- |
|  |  |  |  |

(l) β-LACTOGLOBULIN

|  |  |  |  |
| --- | --- | --- | --- |
|  |  |  |  |

(m) BLAGLET 1:20

|  |  |  |  |
| --- | --- | --- | --- |
|  |  |  |  |

(n) BLAGLET 1:40

|  |  |  |  |
| --- | --- | --- | --- |
|  |  |  |  |

(o) BLAGLET 1:60

|  |  |  |  |
| --- | --- | --- | --- |
|  |  |  |  |

(p) BLAGLET 1:80

|  |  |  |  |
| --- | --- | --- | --- |
|  |  |  |  |

*Supplementary Figure K. For a given BAMLET formulation, coloured models derived by DAMMIN from P(r) function derived from experimental scattering for BAMLET species (pH 12, 4°C) having input protein to lipid molecular ratios of (a) 1:2, (b) 1:5, (c) 1:5.625, (d) 1:6.25, (e) 1:7.5, (f) 1:8.75, (g) 1:10, (h) 1:12.5, (i) 1:15, (j) 1:17.5, and (k) 1:20, for (l) β-lactoglobulin (pH 12, β-mercaptoethanol, 10°C), and BLAGLET species (pH 12, β-mercaptoethanol, 10°C) having input protein to lipid molecular ratios of (m) 1:20, (n) 1:40, (o) 1:60, and (p) 1:80. Under each model is the FoXS server (Schneidman-Duhovny et al. 2013, Schneidman-Duhovny et al. 2016) simulated scattering curve, superimposed on the actual scattering curve, showing that simulated scattering matches actual scattering in the low- and mid-q regions that represent intramolecular distances of 20 – 100 Å (d = 2π/q), and thus the model is plausible. Simulated scattering overlaps actual scattering for most models in the high-q region of q > 0.3 Å representing short intramolecular distances. For some high oleic acid content species' models, high-q simulated scattering by FoXS server contains features and the curve is below the featureless high-q curve of the actual scattering whereas the simulated scattering by the DAMMIN program that produced the models is more featureless and better overlaps actual scattering. These differences in simulated scattering at high-q are due to differences in simulation treatment of short intramolecular scattering.*

| (a) | (b) |
| --- | --- |
| (c) | (d) |

*Supplementary Figure L. SEM of (a) BAMLET-high, (b) BLAGLET-high, (c) bovine α-lactalbumin powder, and (d) bovine β-lactoglobulin powder, at magnification 20 000×, 20 000×, 255×, and 320× respectively, using 10 kV beam accelerating voltage for all samples except β-lactoglobulin for which 5 kV was used in order to reduce shimmering of electrons not absorbed by the sample. The SEM image for β-lactoglobulin exhibits shimmering of electrons that is not present in the image for α-lactalbumin. This may indicate that the β-lactoglobulin sample is less conducting of the SEM electrons than the α-lactalbumin sample.*

| (a) | (b) |
| --- | --- |
| (c) | (d) |
| (e) | (f) |
| (g) | (h) |

*Supplementary Figure M. CD spectra of BLAGLET-high in pH 7 increasing temperature from 20 to 95°C (a,c,e,f) or decreasing temperature from 95°C to 20°C (b,d,f,h) at a rate of ~5°C per 5 min, showing that there is a small, incremental change in CD spectrum as temperature changes, indicating changes in secondary structure.*

|  | *Supplementary Figure N. Estimation of BLAGLET-high secondary structure from BLAGLET CD data presented in Supplementary Figure S9, using the Yang method (Venyaminov et al. 1993) as supplied with the CD instrument. β-Sheet structure is present at 20°C, is lost at 95°C, and is regained when temperature returns to 20°C, and this unfolding/refolding occurs over multiple heating cycles.* |
| --- | --- |

| (a) | (b) |
| --- | --- |
| (c) | (d) |
| (e) | (f) |
| (g) | (h) |

*Supplementary Figure O. CD spectra of apo-β-lactoglobulin at pH 7 increasing temperature from 20 to 95°C (a,c,e,f) or decreasing temperature from 95°C to 20°C (b,d,f,h) at a rate of ~5°C per 5 min, showing that at each temperature there has been a small, incremental change in CD spectrum, indicating changes in secondary structure.*
