# Supplementary figures and images for: BAMLET kills chemotherapy-resistant mesothelioma cells, holding oleic acid in an activated cytotoxic state
Source: PLoS One. 2018 Aug 29;13(8):e0203003. doi: 10.1371/journal.pone.0203003 (PMC6114908; doi:10.1371/journal.pone.0203003)

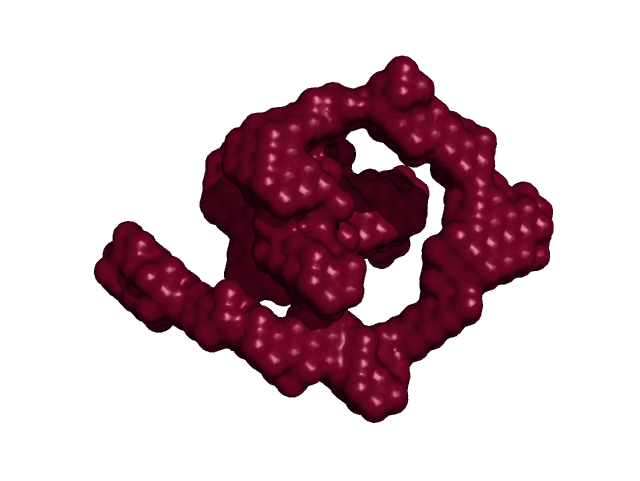

Supplement: S2 Dataset — (TGZ) [file pone.0203003.s003.tgz › BAMLET_and_BLAGLET_SAXS_and_models/SAXS_models_for_BAMLETs_pH12_4C/bam1o17p5_09.png]

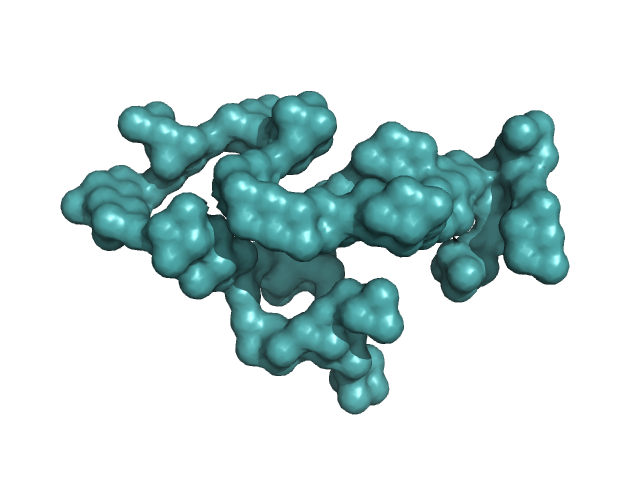

Supplement: S2 Dataset — (TGZ) [file pone.0203003.s003.tgz › BAMLET_and_BLAGLET_SAXS_and_models/SAXS_models_for_BAMLETs_pH12_4C/bam1o5_11.png]

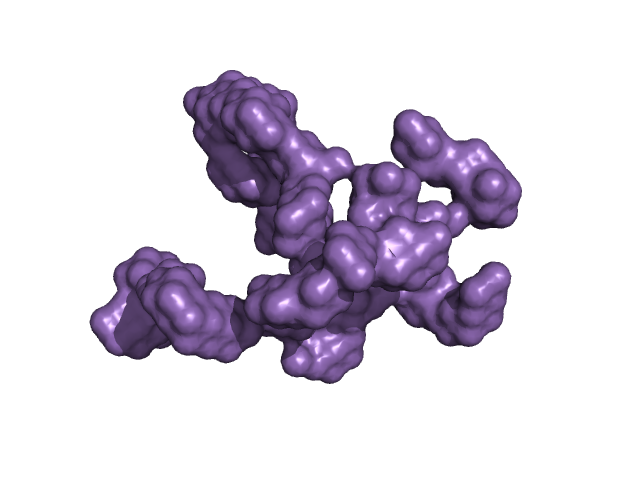

Supplement: S2 Dataset — (TGZ) [file pone.0203003.s003.tgz › BAMLET_and_BLAGLET_SAXS_and_models/SAXS_models_for_BAMLETs_pH12_4C/bam1o12p5_05.png]

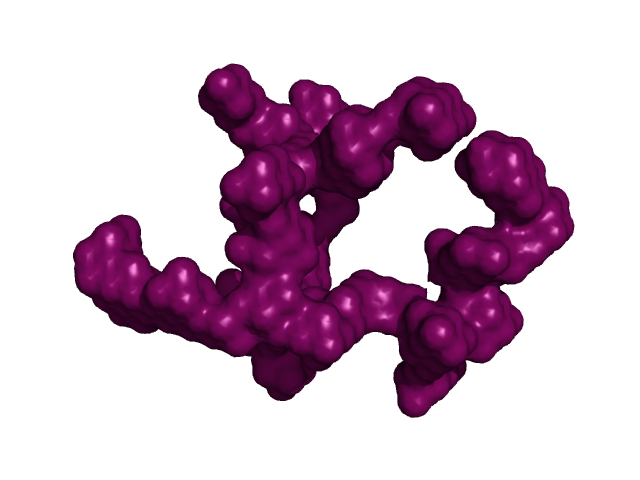

Supplement: S2 Dataset — (TGZ) [file pone.0203003.s003.tgz › BAMLET_and_BLAGLET_SAXS_and_models/SAXS_models_for_BAMLETs_pH12_4C/bam1o15_20.png]

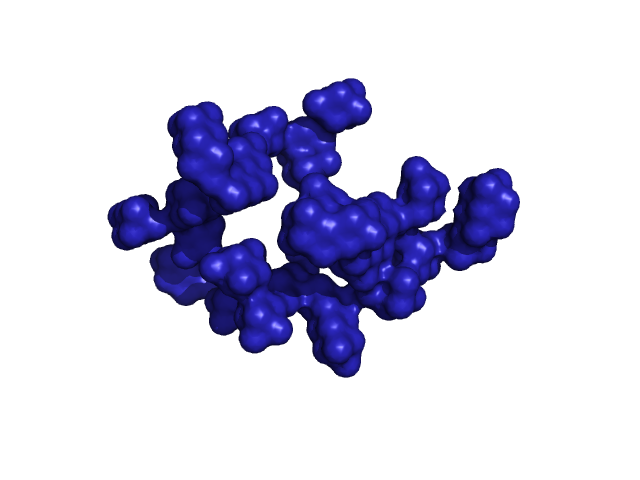

Supplement: S2 Dataset — (TGZ) [file pone.0203003.s003.tgz › BAMLET_and_BLAGLET_SAXS_and_models/SAXS_models_for_BAMLETs_pH12_4C/bam1o7p5_10.png]

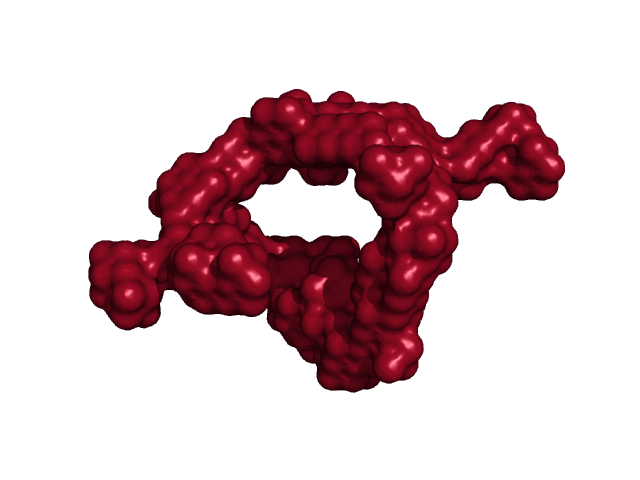

Supplement: S2 Dataset — (TGZ) [file pone.0203003.s003.tgz › BAMLET_and_BLAGLET_SAXS_and_models/SAXS_models_for_BAMLETs_pH12_4C/bam1o20_10.png]

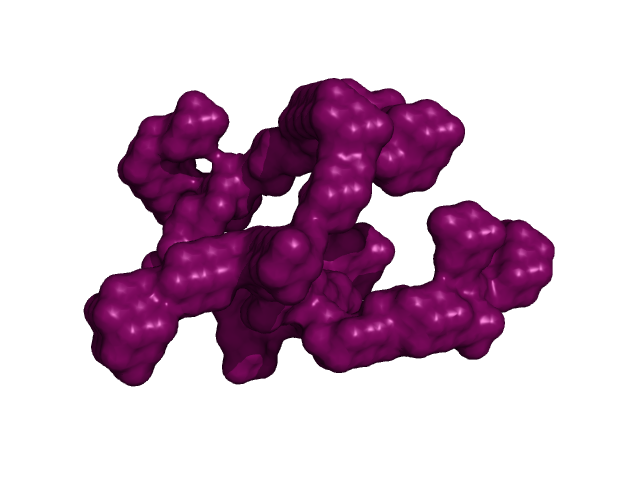

Supplement: S2 Dataset — (TGZ) [file pone.0203003.s003.tgz › BAMLET_and_BLAGLET_SAXS_and_models/SAXS_models_for_BAMLETs_pH12_4C/bam1o15_09.png]

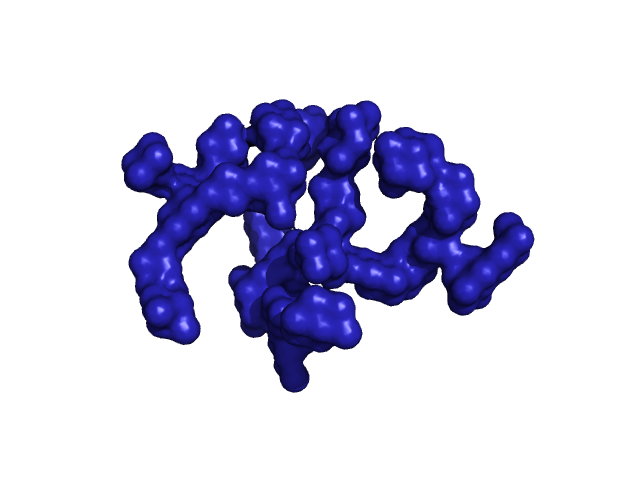

Supplement: S2 Dataset — (TGZ) [file pone.0203003.s003.tgz › BAMLET_and_BLAGLET_SAXS_and_models/SAXS_models_for_BAMLETs_pH12_4C/bam1o7p5_04.png]

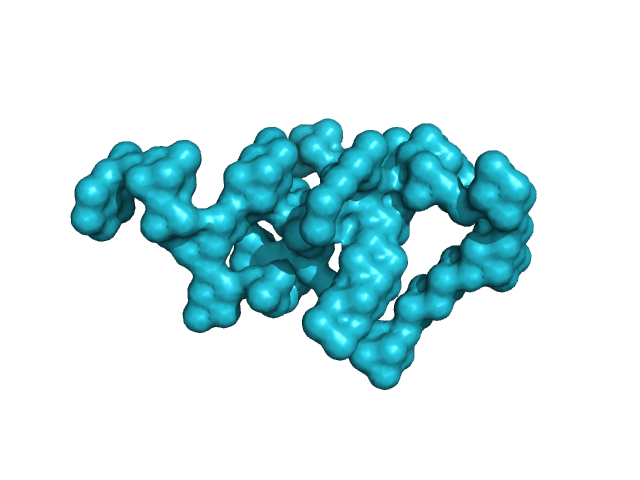

Supplement: S2 Dataset — (TGZ) [file pone.0203003.s003.tgz › BAMLET_and_BLAGLET_SAXS_and_models/SAXS_models_for_BAMLETs_pH12_4C/bam1o2_12.png]

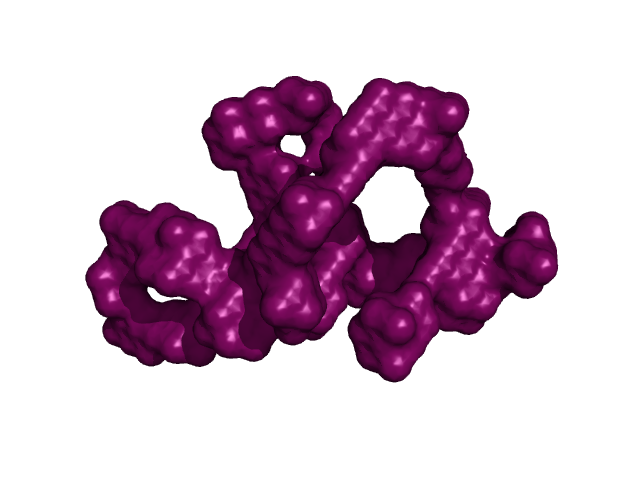

Supplement: S2 Dataset — (TGZ) [file pone.0203003.s003.tgz › BAMLET_and_BLAGLET_SAXS_and_models/SAXS_models_for_BAMLETs_pH12_4C/bam1o15_02.png]

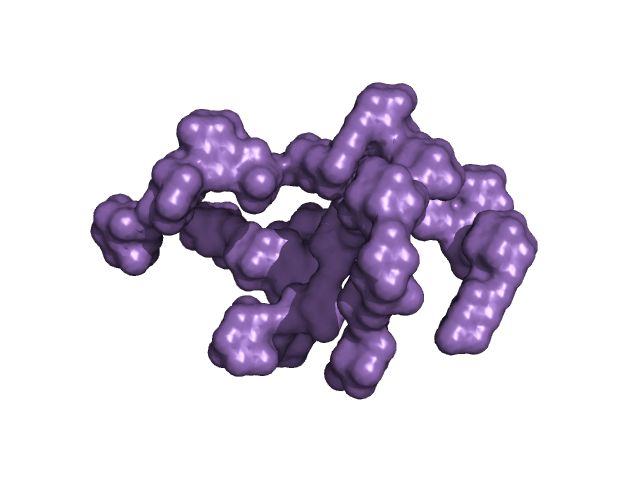

Supplement: S2 Dataset — (TGZ) [file pone.0203003.s003.tgz › BAMLET_and_BLAGLET_SAXS_and_models/SAXS_models_for_BAMLETs_pH12_4C/bam1o12p5_13.png]

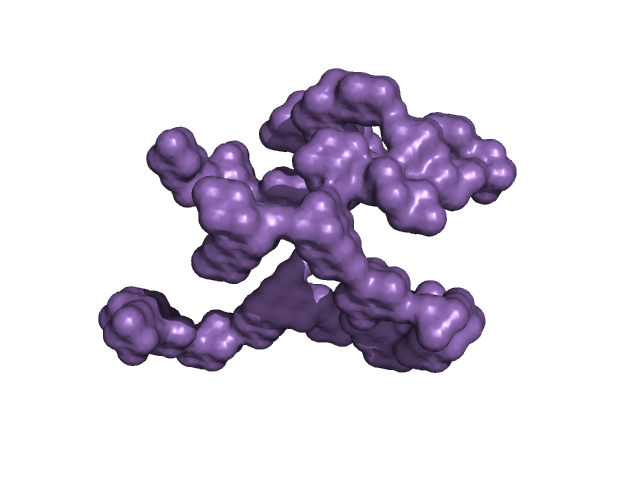

Supplement: S2 Dataset — (TGZ) [file pone.0203003.s003.tgz › BAMLET_and_BLAGLET_SAXS_and_models/SAXS_models_for_BAMLETs_pH12_4C/bam1o12p5_20.png]

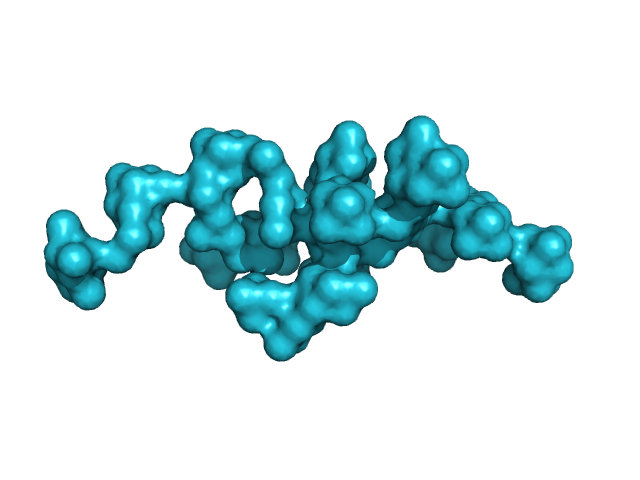

Supplement: S2 Dataset — (TGZ) [file pone.0203003.s003.tgz › BAMLET_and_BLAGLET_SAXS_and_models/SAXS_models_for_BAMLETs_pH12_4C/bam1o2_05.png]

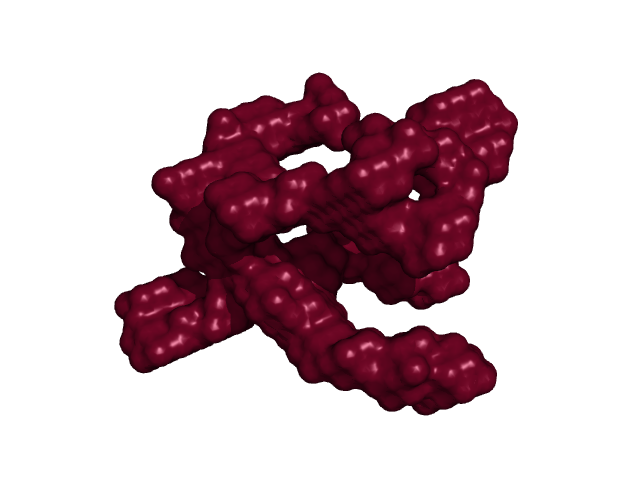

Supplement: S2 Dataset — (TGZ) [file pone.0203003.s003.tgz › BAMLET_and_BLAGLET_SAXS_and_models/SAXS_models_for_BAMLETs_pH12_4C/bam1o17p5_08.png]

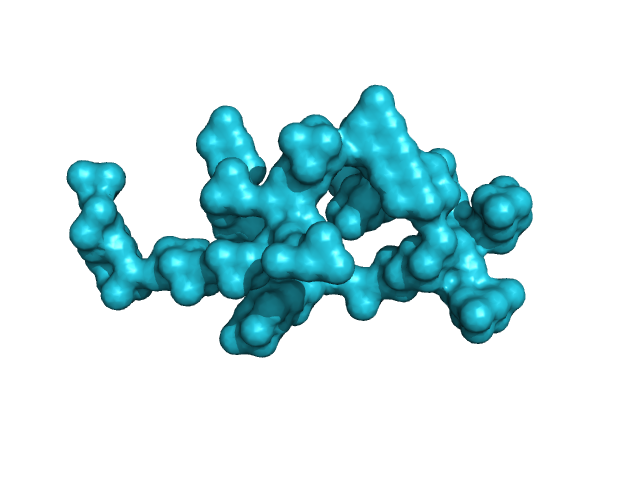

Supplement: S2 Dataset — (TGZ) [file pone.0203003.s003.tgz › BAMLET_and_BLAGLET_SAXS_and_models/SAXS_models_for_BAMLETs_pH12_4C/bam1o2_16.png]

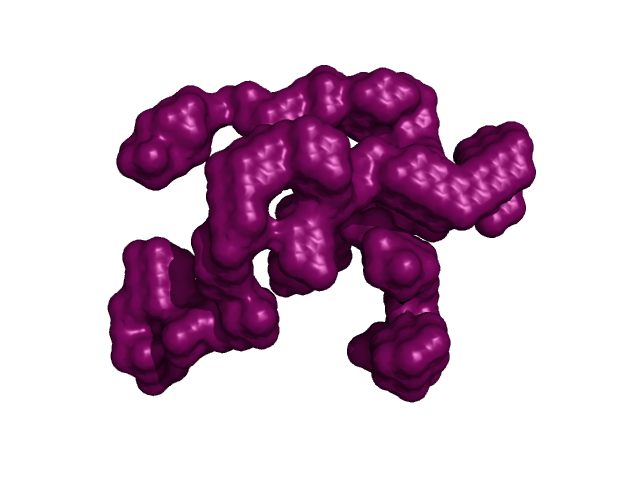

Supplement: S2 Dataset — (TGZ) [file pone.0203003.s003.tgz › BAMLET_and_BLAGLET_SAXS_and_models/SAXS_models_for_BAMLETs_pH12_4C/bam1o15_17.png]

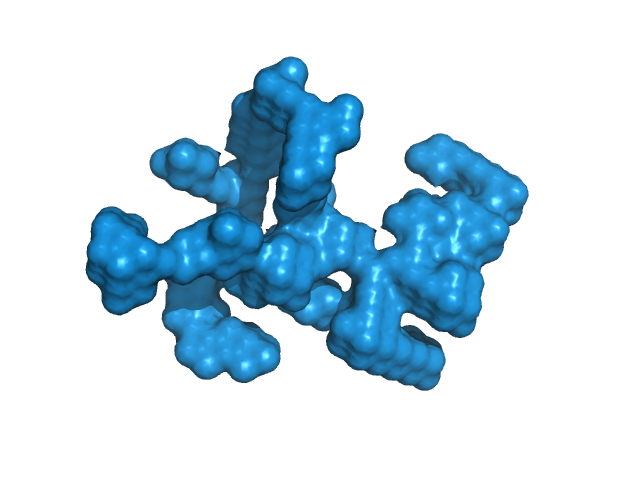

Supplement: S2 Dataset — (TGZ) [file pone.0203003.s003.tgz › BAMLET_and_BLAGLET_SAXS_and_models/SAXS_models_for_BAMLETs_pH12_4C/bam1o6p25_10.png]

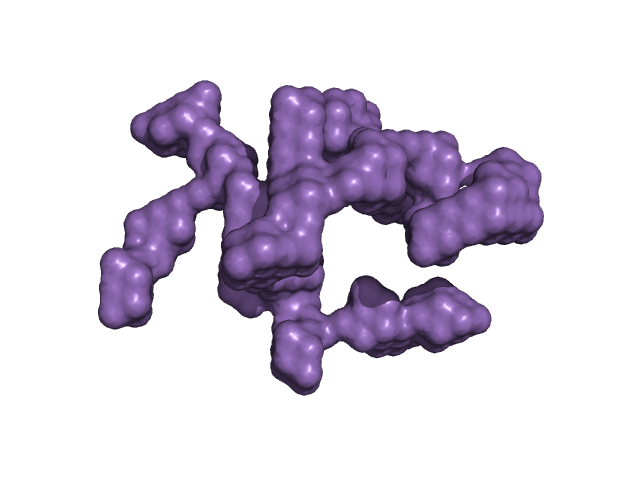

Supplement: S2 Dataset — (TGZ) [file pone.0203003.s003.tgz › BAMLET_and_BLAGLET_SAXS_and_models/SAXS_models_for_BAMLETs_pH12_4C/bam1o12p5_19.png]

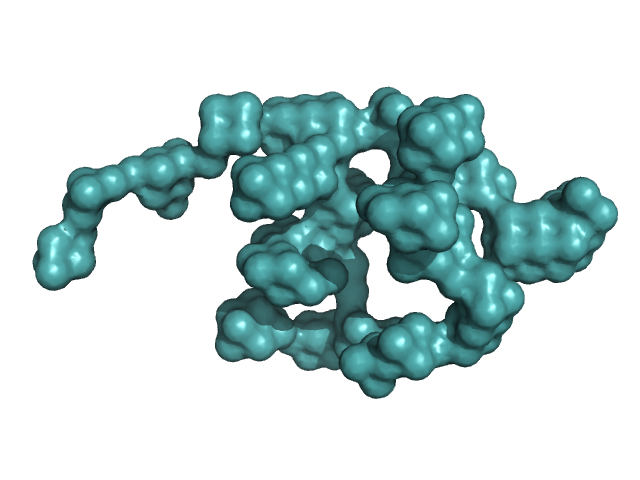

Supplement: S2 Dataset — (TGZ) [file pone.0203003.s003.tgz › BAMLET_and_BLAGLET_SAXS_and_models/SAXS_models_for_BAMLETs_pH12_4C/bam1o5_03.png]

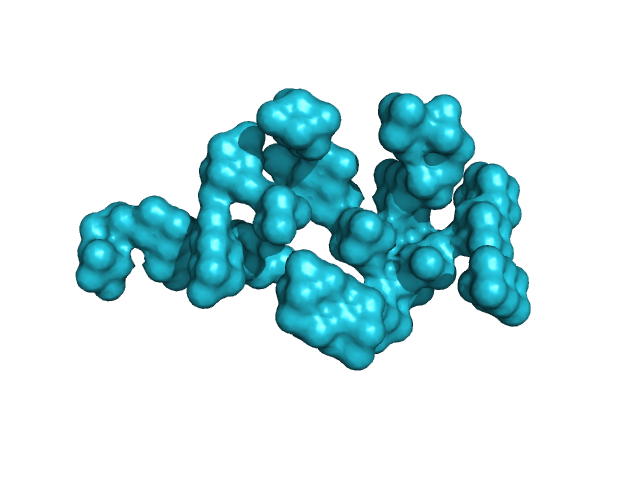

Supplement: S2 Dataset — (TGZ) [file pone.0203003.s003.tgz › BAMLET_and_BLAGLET_SAXS_and_models/SAXS_models_for_BAMLETs_pH12_4C/bam1o2_19.png]

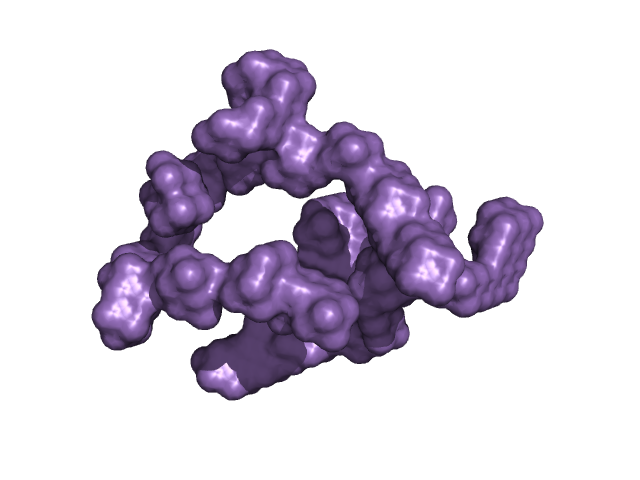

Supplement: S2 Dataset — (TGZ) [file pone.0203003.s003.tgz › BAMLET_and_BLAGLET_SAXS_and_models/SAXS_models_for_BAMLETs_pH12_4C/bam1o12p5_16.png]

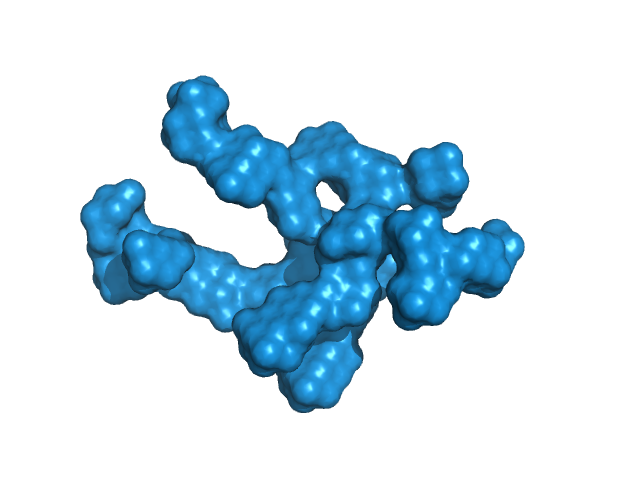

Supplement: S2 Dataset — (TGZ) [file pone.0203003.s003.tgz › BAMLET_and_BLAGLET_SAXS_and_models/SAXS_models_for_BAMLETs_pH12_4C/bam1o6p25_13.png]

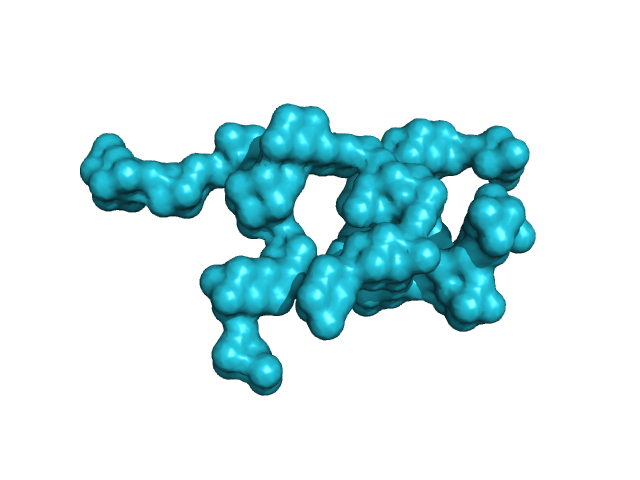

Supplement: S2 Dataset — (TGZ) [file pone.0203003.s003.tgz › BAMLET_and_BLAGLET_SAXS_and_models/SAXS_models_for_BAMLETs_pH12_4C/bam1o2_08.png]

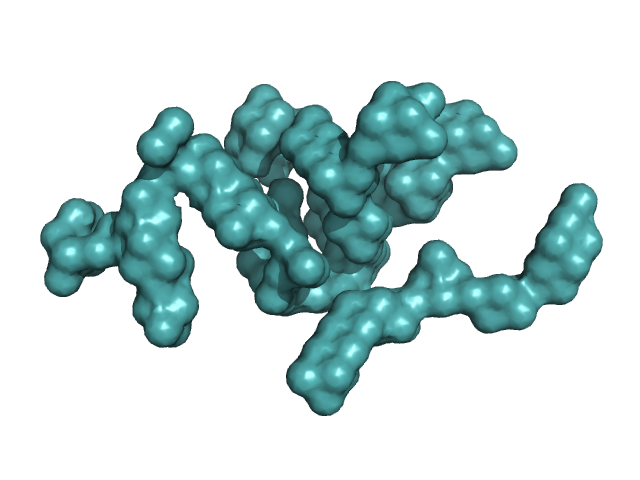

Supplement: S2 Dataset — (TGZ) [file pone.0203003.s003.tgz › BAMLET_and_BLAGLET_SAXS_and_models/SAXS_models_for_BAMLETs_pH12_4C/bam1o5_18.png]

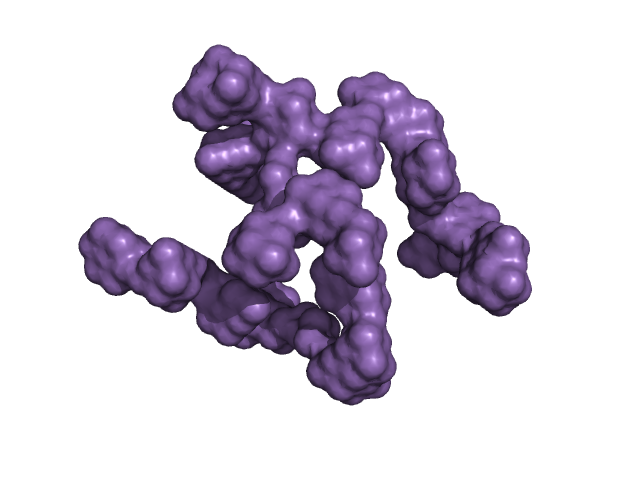

Supplement: S2 Dataset — (TGZ) [file pone.0203003.s003.tgz › BAMLET_and_BLAGLET_SAXS_and_models/SAXS_models_for_BAMLETs_pH12_4C/bam1o12p5_06.png]

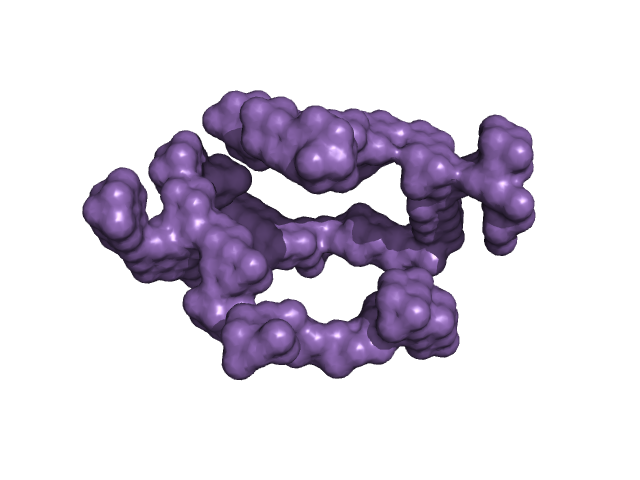

Supplement: S2 Dataset — (TGZ) [file pone.0203003.s003.tgz › BAMLET_and_BLAGLET_SAXS_and_models/SAXS_models_for_BAMLETs_pH12_4C/bam1o12p5_10.png]

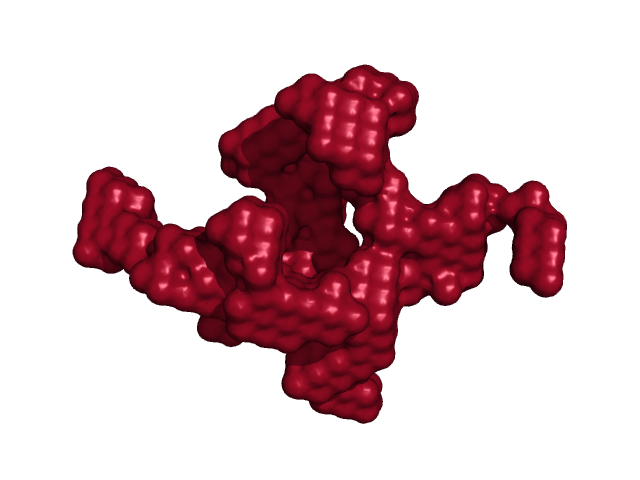

Supplement: S2 Dataset — (TGZ) [file pone.0203003.s003.tgz › BAMLET_and_BLAGLET_SAXS_and_models/SAXS_models_for_BAMLETs_pH12_4C/bam1o20_15.png]

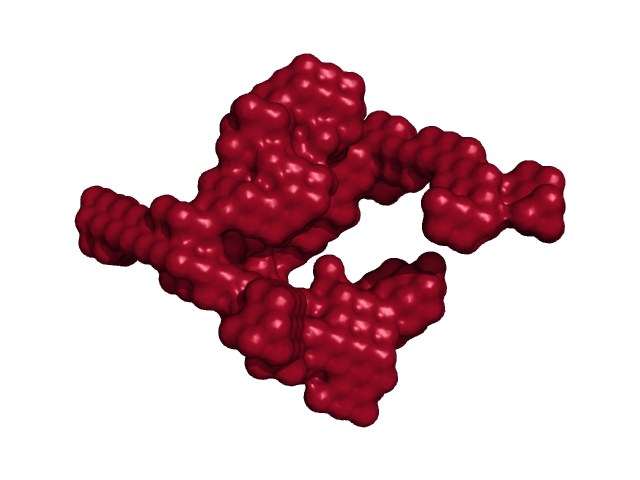

Supplement: S2 Dataset — (TGZ) [file pone.0203003.s003.tgz › BAMLET_and_BLAGLET_SAXS_and_models/SAXS_models_for_BAMLETs_pH12_4C/bam1o20_06.png]

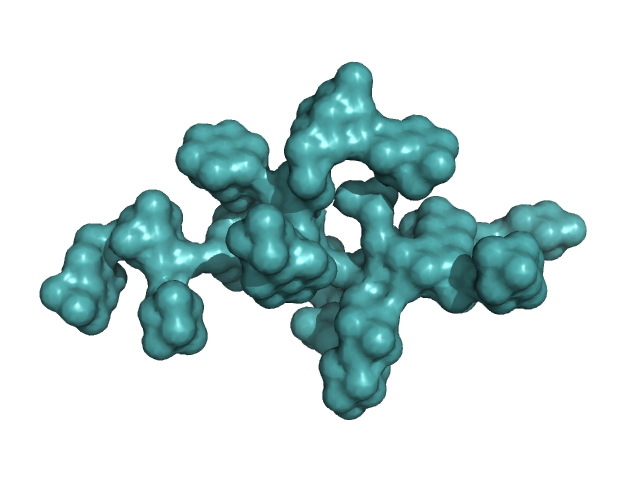

Supplement: S2 Dataset — (TGZ) [file pone.0203003.s003.tgz › BAMLET_and_BLAGLET_SAXS_and_models/SAXS_models_for_BAMLETs_pH12_4C/bam1o5_14.png]

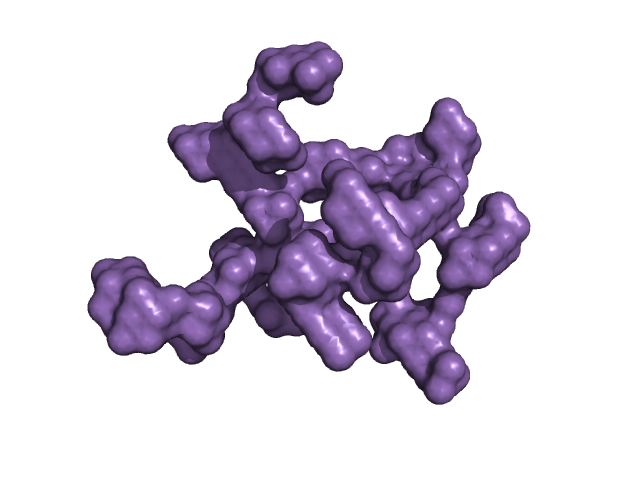

Supplement: S2 Dataset — (TGZ) [file pone.0203003.s003.tgz › BAMLET_and_BLAGLET_SAXS_and_models/SAXS_models_for_BAMLETs_pH12_4C/bam1o12p5_17.png]

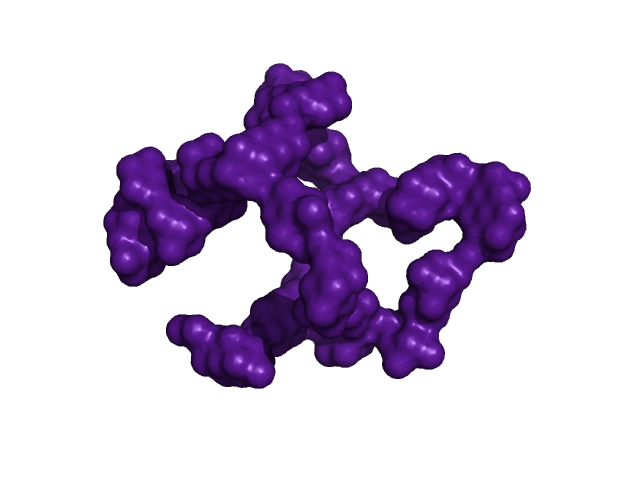

Supplement: S2 Dataset — (TGZ) [file pone.0203003.s003.tgz › BAMLET_and_BLAGLET_SAXS_and_models/SAXS_models_for_BAMLETs_pH12_4C/bam1o10_16.png]

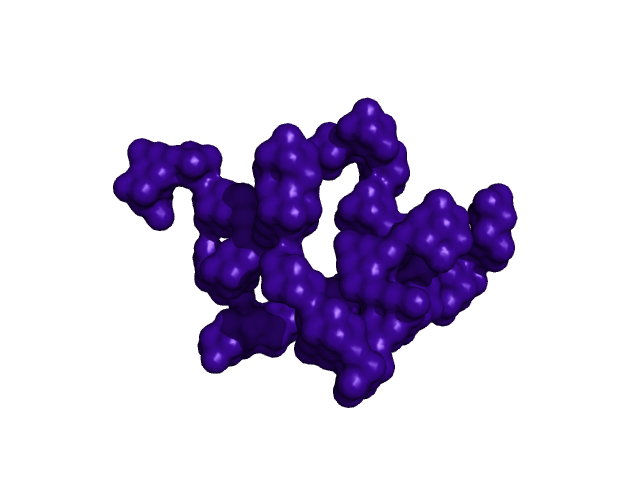

Supplement: S2 Dataset — (TGZ) [file pone.0203003.s003.tgz › BAMLET_and_BLAGLET_SAXS_and_models/SAXS_models_for_BAMLETs_pH12_4C/bam1o8p75_05.png]

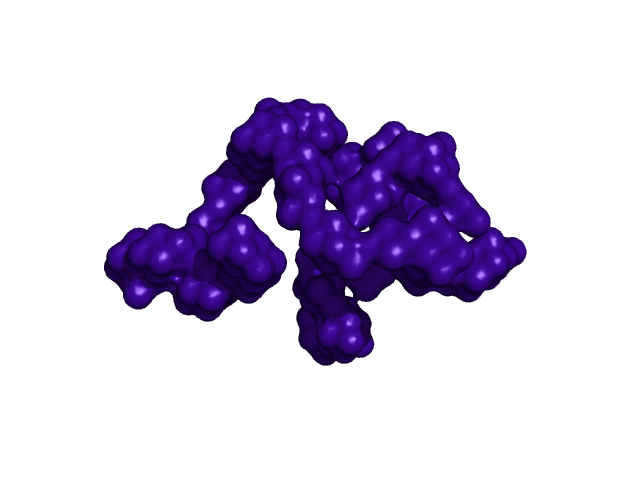

Supplement: S2 Dataset — (TGZ) [file pone.0203003.s003.tgz › BAMLET_and_BLAGLET_SAXS_and_models/SAXS_models_for_BAMLETs_pH12_4C/bam1o8p75_14.png]

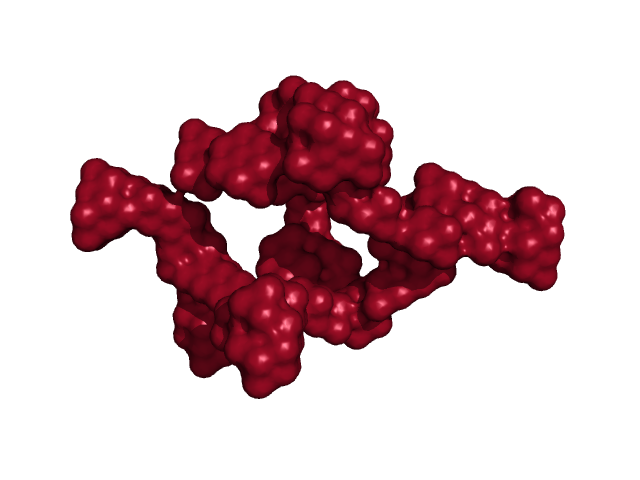

Supplement: S2 Dataset — (TGZ) [file pone.0203003.s003.tgz › BAMLET_and_BLAGLET_SAXS_and_models/SAXS_models_for_BAMLETs_pH12_4C/bam1o20_09.png]

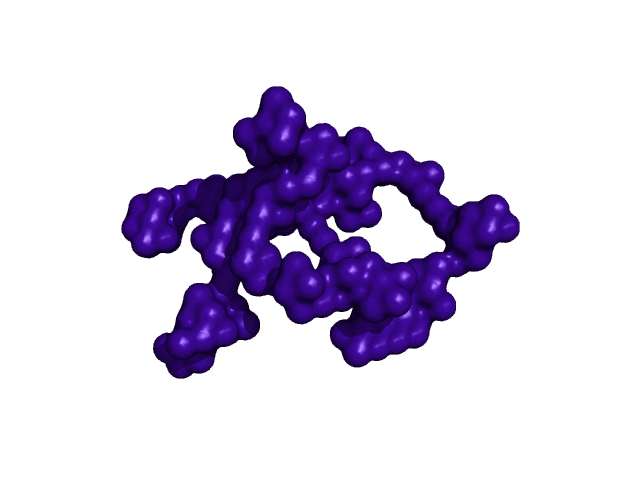

Supplement: S2 Dataset — (TGZ) [file pone.0203003.s003.tgz › BAMLET_and_BLAGLET_SAXS_and_models/SAXS_models_for_BAMLETs_pH12_4C/bam1o8p75_03.png]

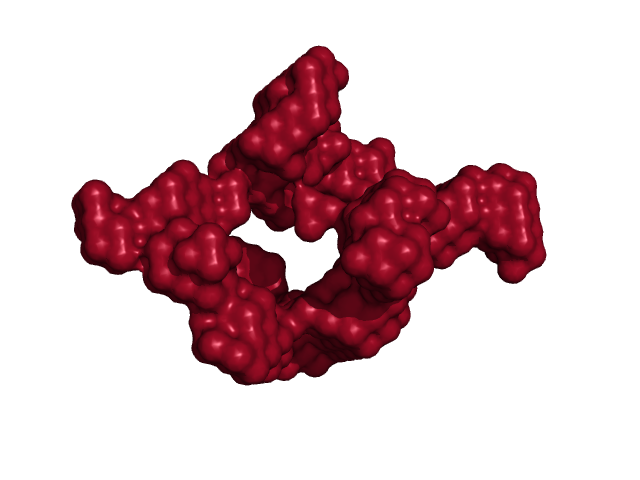

Supplement: S2 Dataset — (TGZ) [file pone.0203003.s003.tgz › BAMLET_and_BLAGLET_SAXS_and_models/SAXS_models_for_BAMLETs_pH12_4C/bam1o20_19.png]

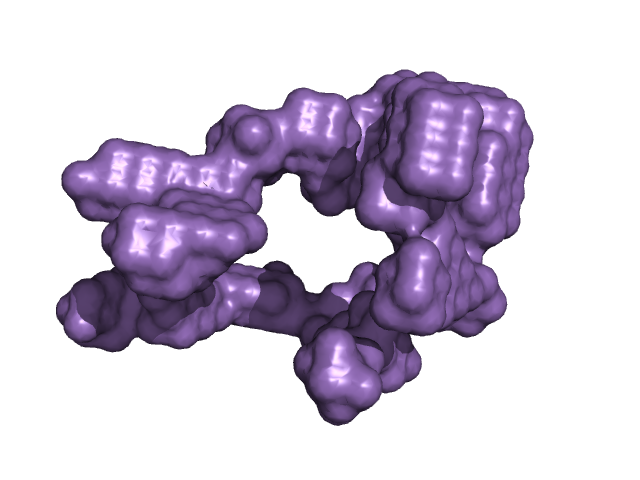

Supplement: S2 Dataset — (TGZ) [file pone.0203003.s003.tgz › BAMLET_and_BLAGLET_SAXS_and_models/SAXS_models_for_BAMLETs_pH12_4C/bam1o12p5_00.png]

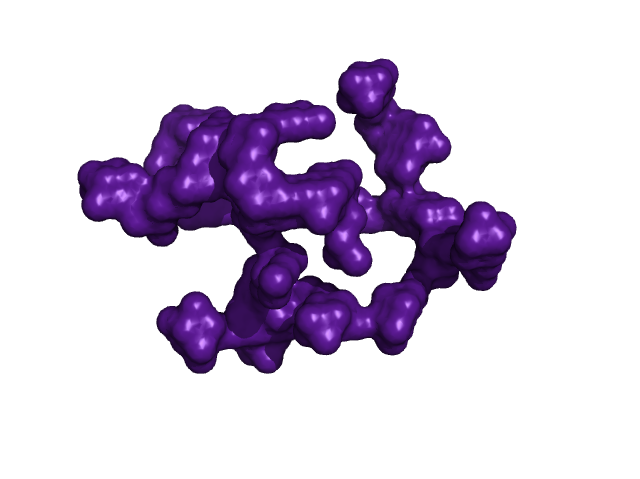

Supplement: S2 Dataset — (TGZ) [file pone.0203003.s003.tgz › BAMLET_and_BLAGLET_SAXS_and_models/SAXS_models_for_BAMLETs_pH12_4C/bam1o10_19.png]

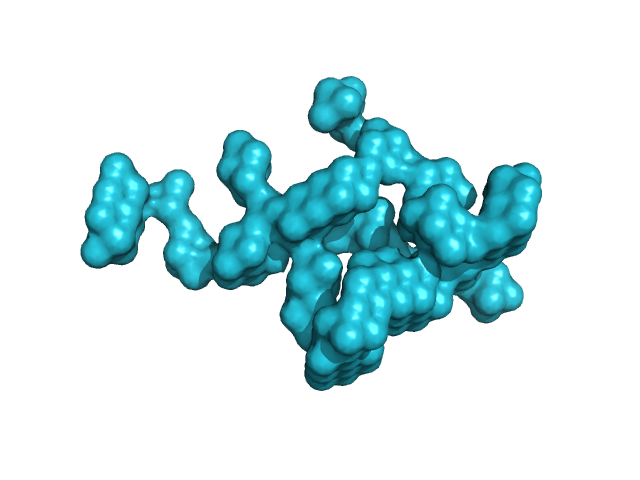

Supplement: S2 Dataset — (TGZ) [file pone.0203003.s003.tgz › BAMLET_and_BLAGLET_SAXS_and_models/SAXS_models_for_BAMLETs_pH12_4C/bam1o2_09.png]

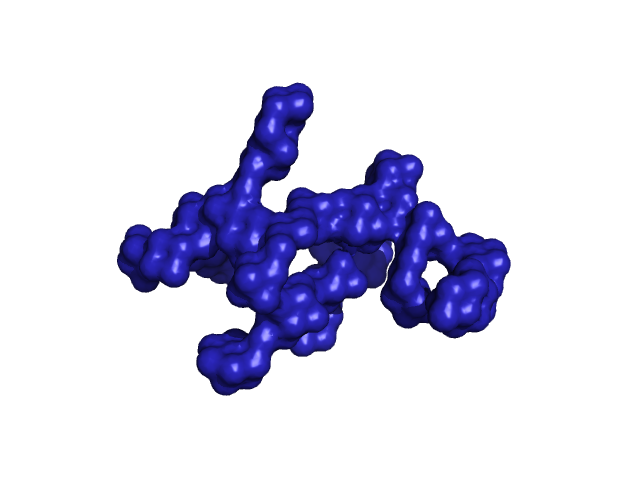

Supplement: S2 Dataset — (TGZ) [file pone.0203003.s003.tgz › BAMLET_and_BLAGLET_SAXS_and_models/SAXS_models_for_BAMLETs_pH12_4C/bam1o7p5_12.png]

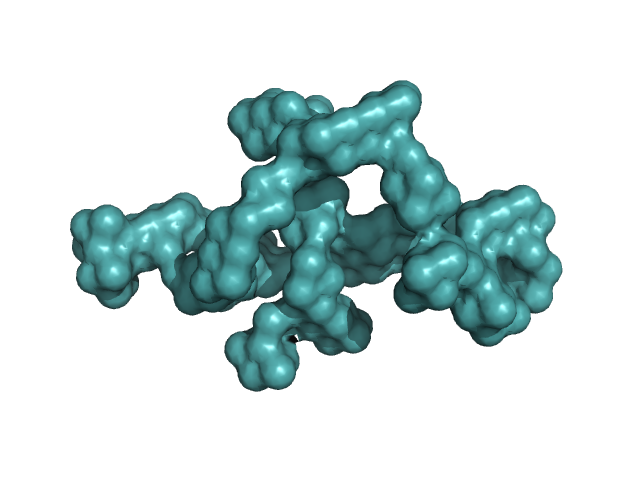

Supplement: S2 Dataset — (TGZ) [file pone.0203003.s003.tgz › BAMLET_and_BLAGLET_SAXS_and_models/SAXS_models_for_BAMLETs_pH12_4C/bam1o5_01.png]

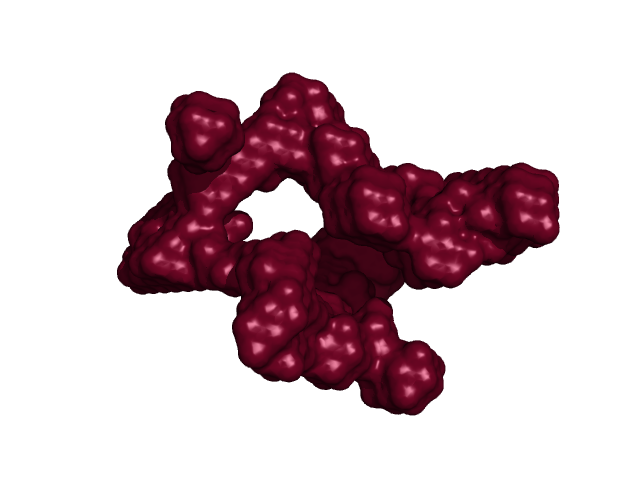

Supplement: S2 Dataset — (TGZ) [file pone.0203003.s003.tgz › BAMLET_and_BLAGLET_SAXS_and_models/SAXS_models_for_BAMLETs_pH12_4C/bam1o17p5_10.png]

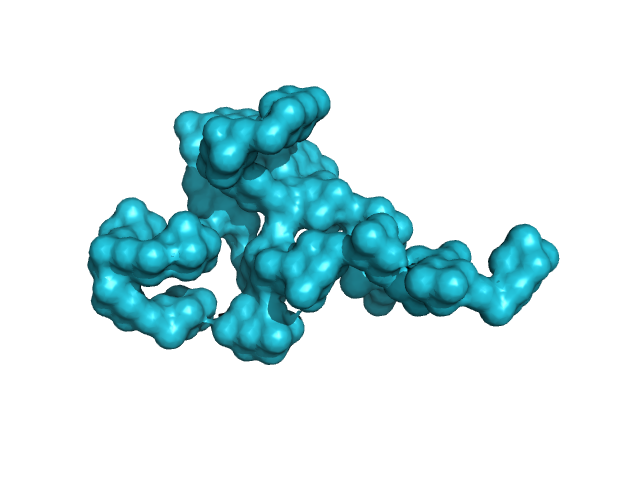

Supplement: S2 Dataset — (TGZ) [file pone.0203003.s003.tgz › BAMLET_and_BLAGLET_SAXS_and_models/SAXS_models_for_BAMLETs_pH12_4C/bam1o2_01.png]

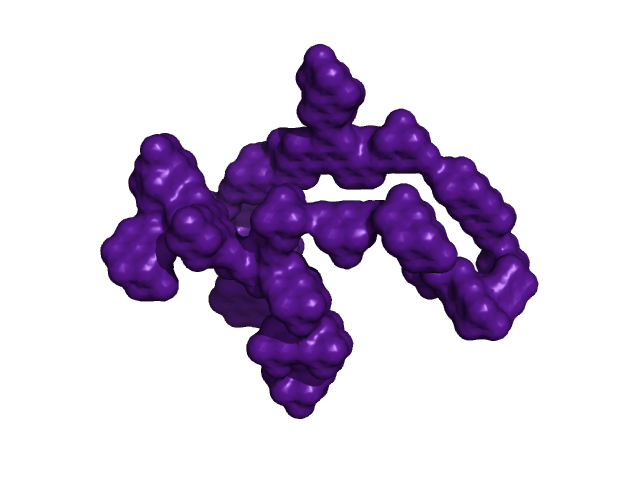

Supplement: S2 Dataset — (TGZ) [file pone.0203003.s003.tgz › BAMLET_and_BLAGLET_SAXS_and_models/SAXS_models_for_BAMLETs_pH12_4C/bam1o10_02.png]

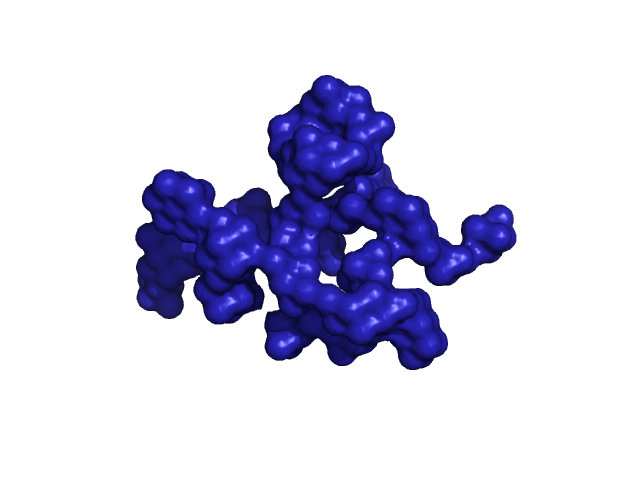

Supplement: S2 Dataset — (TGZ) [file pone.0203003.s003.tgz › BAMLET_and_BLAGLET_SAXS_and_models/SAXS_models_for_BAMLETs_pH12_4C/bam1o7p5_20.png]

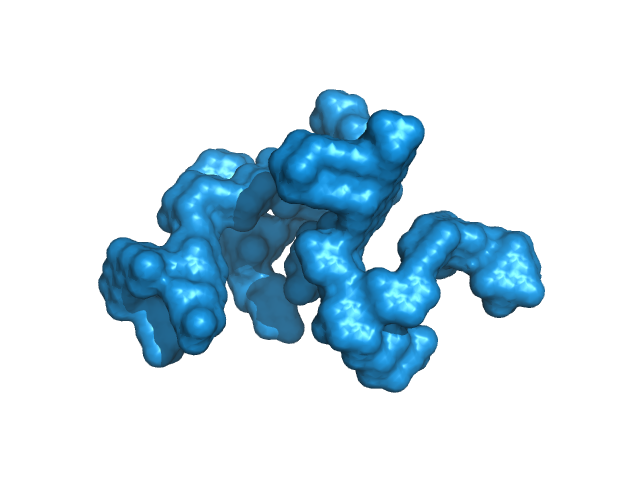

Supplement: S2 Dataset — (TGZ) [file pone.0203003.s003.tgz › BAMLET_and_BLAGLET_SAXS_and_models/SAXS_models_for_BAMLETs_pH12_4C/bam1o6p25_02.png]

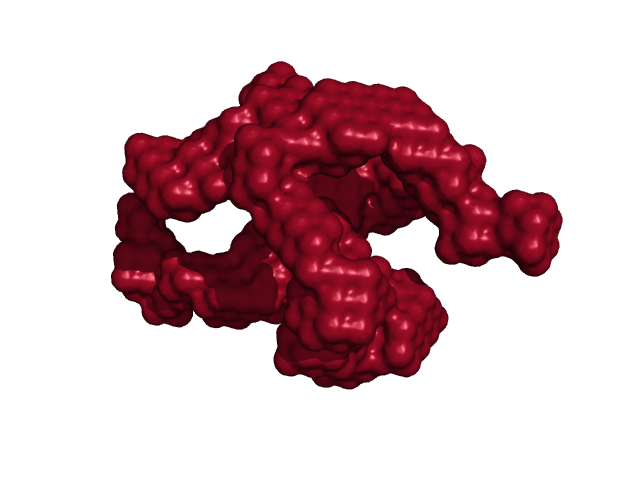

Supplement: S2 Dataset — (TGZ) [file pone.0203003.s003.tgz › BAMLET_and_BLAGLET_SAXS_and_models/SAXS_models_for_BAMLETs_pH12_4C/bam1o20_03.png]

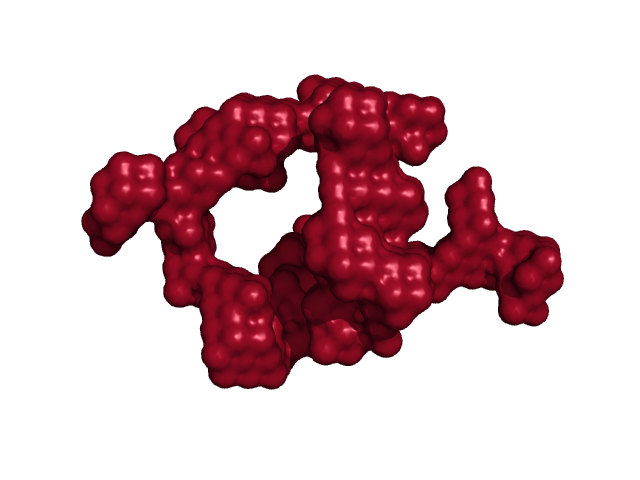

Supplement: S2 Dataset — (TGZ) [file pone.0203003.s003.tgz › BAMLET_and_BLAGLET_SAXS_and_models/SAXS_models_for_BAMLETs_pH12_4C/bam1o20_11.png]

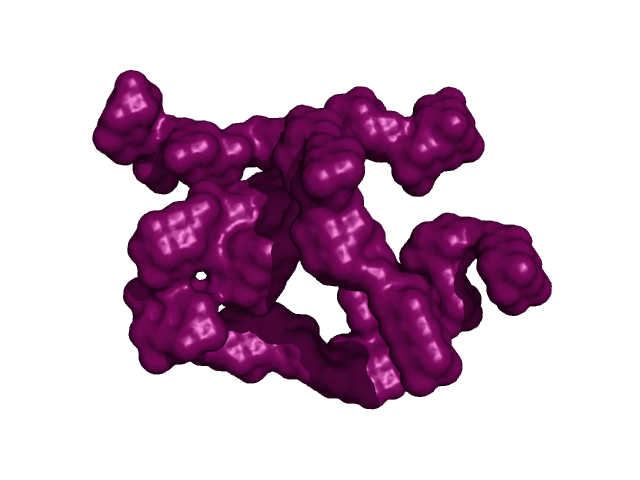

Supplement: S2 Dataset — (TGZ) [file pone.0203003.s003.tgz › BAMLET_and_BLAGLET_SAXS_and_models/SAXS_models_for_BAMLETs_pH12_4C/bam1o15_15.png]

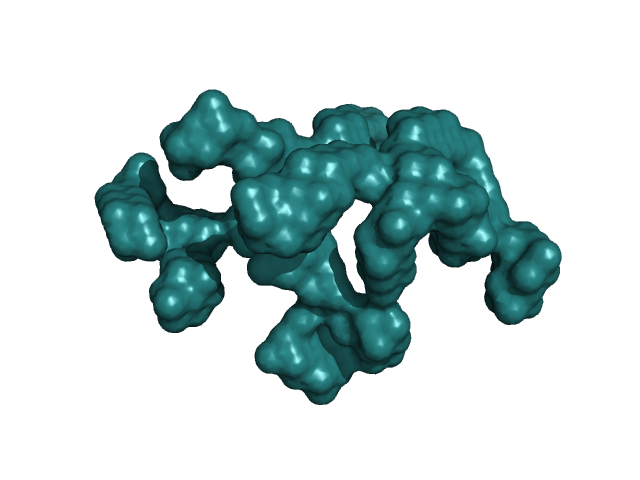

Supplement: S2 Dataset — (TGZ) [file pone.0203003.s003.tgz › BAMLET_and_BLAGLET_SAXS_and_models/SAXS_models_for_BAMLETs_pH12_4C/bam1o5p625_15.png]

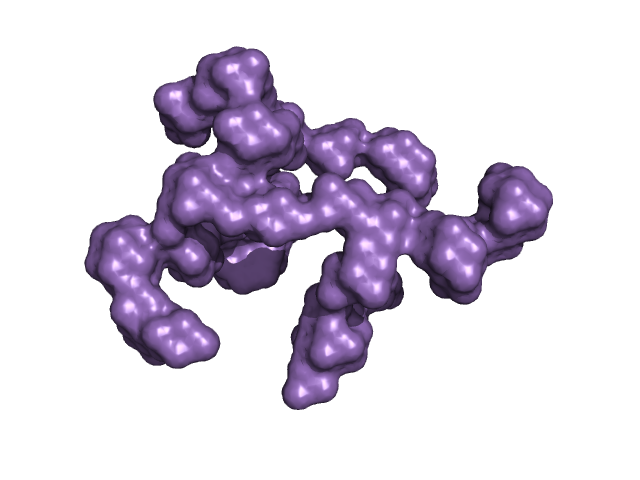

Supplement: S2 Dataset — (TGZ) [file pone.0203003.s003.tgz › BAMLET_and_BLAGLET_SAXS_and_models/SAXS_models_for_BAMLETs_pH12_4C/bam1o12p5_14.png]

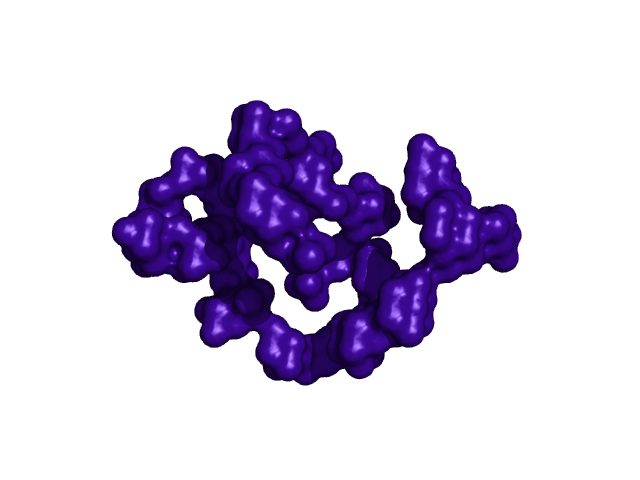

Supplement: S2 Dataset — (TGZ) [file pone.0203003.s003.tgz › BAMLET_and_BLAGLET_SAXS_and_models/SAXS_models_for_BAMLETs_pH12_4C/bam1o8p75_02.png]

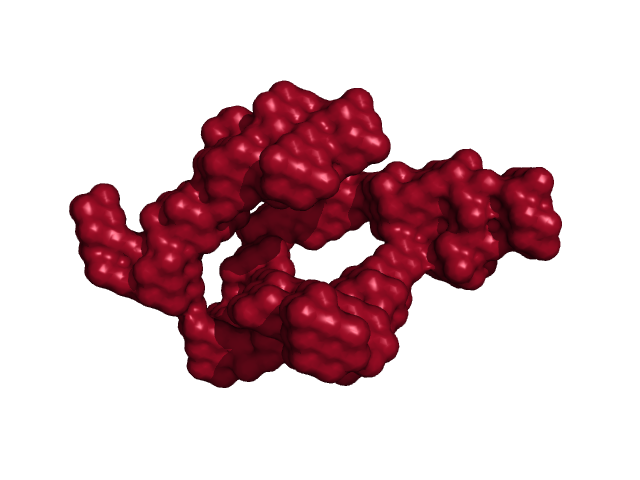

Supplement: S2 Dataset — (TGZ) [file pone.0203003.s003.tgz › BAMLET_and_BLAGLET_SAXS_and_models/SAXS_models_for_BAMLETs_pH12_4C/bam1o20_12.png]

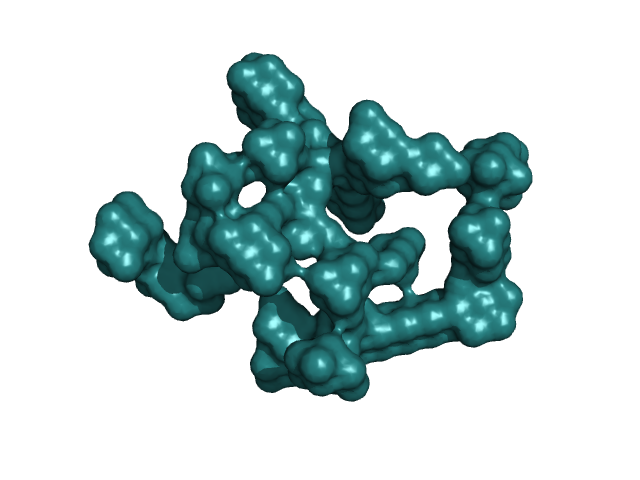

Supplement: S2 Dataset — (TGZ) [file pone.0203003.s003.tgz › BAMLET_and_BLAGLET_SAXS_and_models/SAXS_models_for_BAMLETs_pH12_4C/bam1o5p625_16.png]

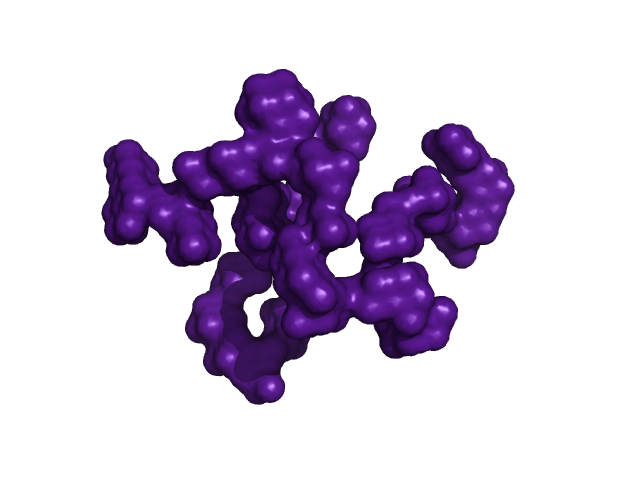

Supplement: S2 Dataset — (TGZ) [file pone.0203003.s003.tgz › BAMLET_and_BLAGLET_SAXS_and_models/SAXS_models_for_BAMLETs_pH12_4C/bam1o10_14.png]

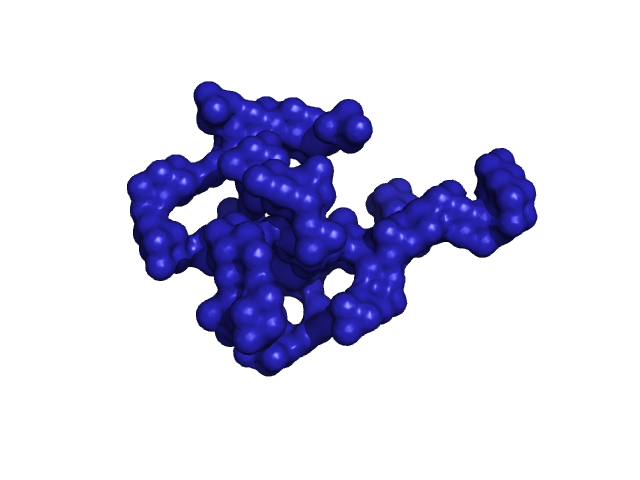

Supplement: S2 Dataset — (TGZ) [file pone.0203003.s003.tgz › BAMLET_and_BLAGLET_SAXS_and_models/SAXS_models_for_BAMLETs_pH12_4C/bam1o7p5_19.png]

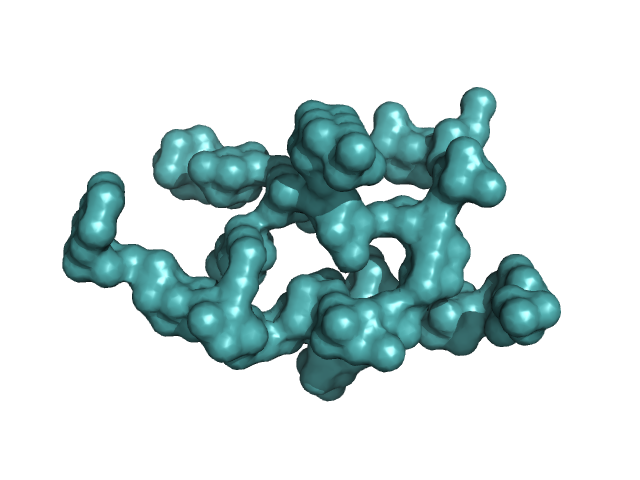

Supplement: S2 Dataset — (TGZ) [file pone.0203003.s003.tgz › BAMLET_and_BLAGLET_SAXS_and_models/SAXS_models_for_BAMLETs_pH12_4C/bam1o5_16.png]

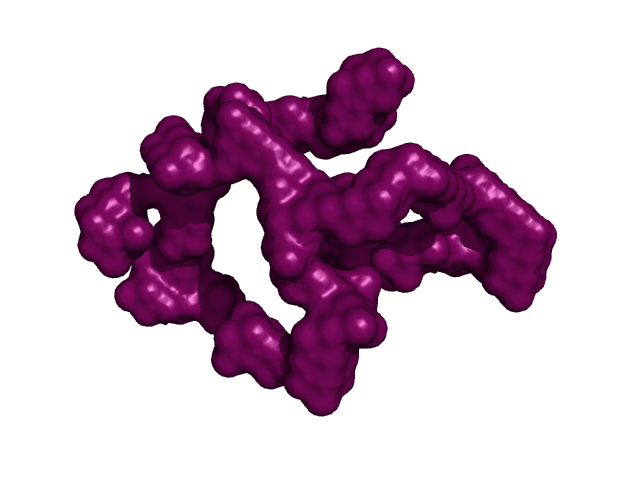

Supplement: S2 Dataset — (TGZ) [file pone.0203003.s003.tgz › BAMLET_and_BLAGLET_SAXS_and_models/SAXS_models_for_BAMLETs_pH12_4C/bam1o15_08.png]

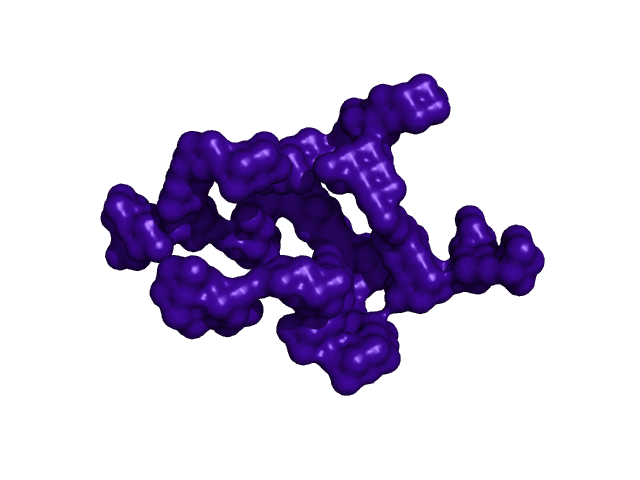

Supplement: S2 Dataset — (TGZ) [file pone.0203003.s003.tgz › BAMLET_and_BLAGLET_SAXS_and_models/SAXS_models_for_BAMLETs_pH12_4C/bam1o8p75_01.png]

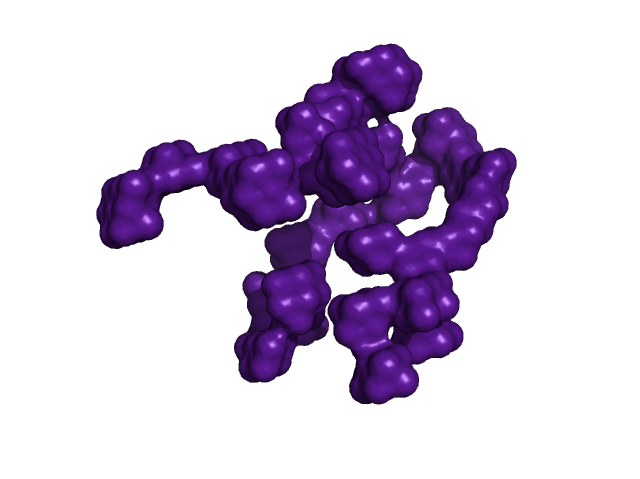

Supplement: S2 Dataset — (TGZ) [file pone.0203003.s003.tgz › BAMLET_and_BLAGLET_SAXS_and_models/SAXS_models_for_BAMLETs_pH12_4C/bam1o10_06.png]

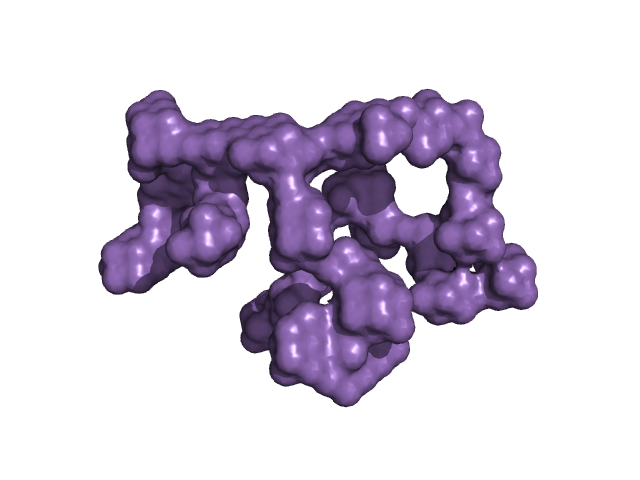

Supplement: S2 Dataset — (TGZ) [file pone.0203003.s003.tgz › BAMLET_and_BLAGLET_SAXS_and_models/SAXS_models_for_BAMLETs_pH12_4C/bam1o12p5_15.png]

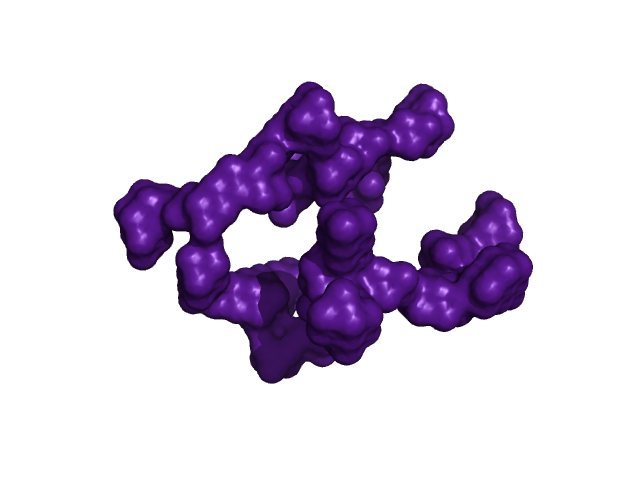

Supplement: S2 Dataset — (TGZ) [file pone.0203003.s003.tgz › BAMLET_and_BLAGLET_SAXS_and_models/SAXS_models_for_BAMLETs_pH12_4C/bam1o10_12.png]

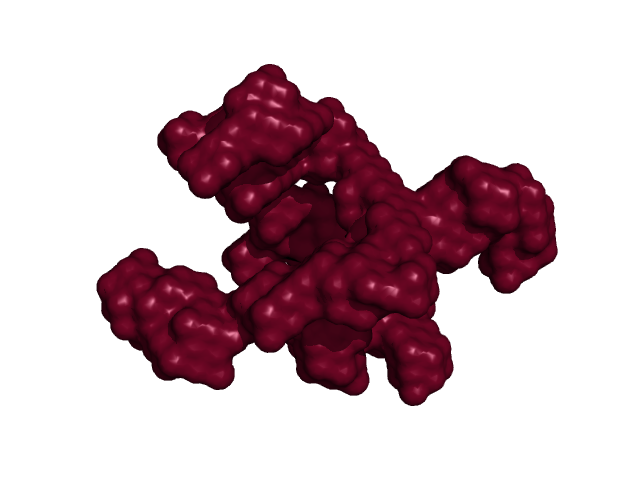

Supplement: S2 Dataset — (TGZ) [file pone.0203003.s003.tgz › BAMLET_and_BLAGLET_SAXS_and_models/SAXS_models_for_BAMLETs_pH12_4C/bam1o17p5_17.png]

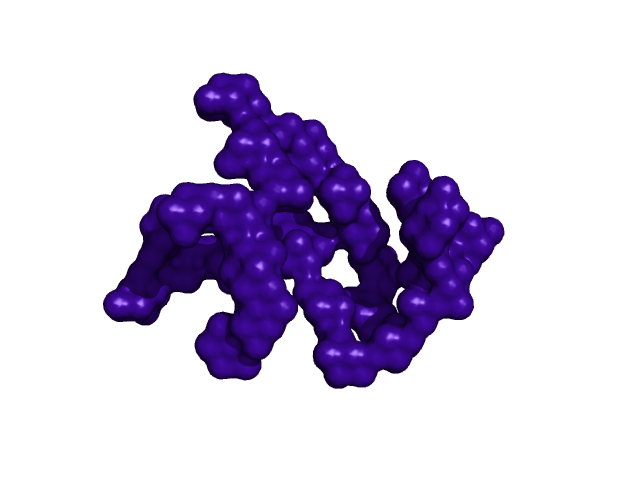

Supplement: S2 Dataset — (TGZ) [file pone.0203003.s003.tgz › BAMLET_and_BLAGLET_SAXS_and_models/SAXS_models_for_BAMLETs_pH12_4C/bam1o8p75_06.png]

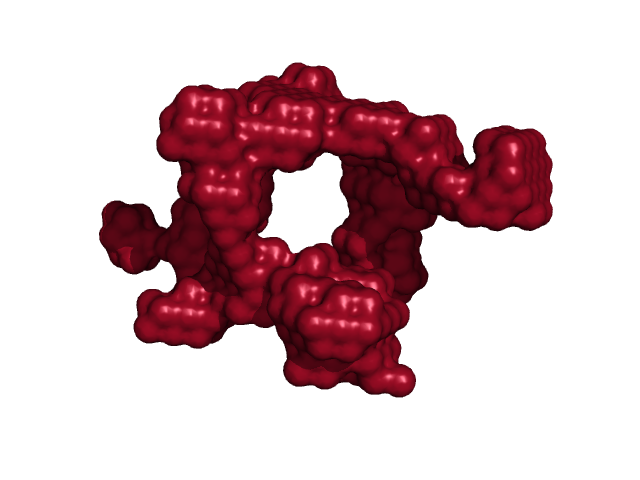

Supplement: S2 Dataset — (TGZ) [file pone.0203003.s003.tgz › BAMLET_and_BLAGLET_SAXS_and_models/SAXS_models_for_BAMLETs_pH12_4C/bam1o20_13.png]

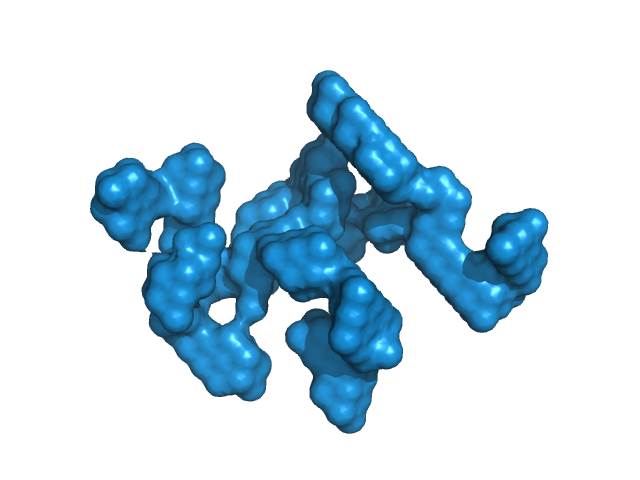

Supplement: S2 Dataset — (TGZ) [file pone.0203003.s003.tgz › BAMLET_and_BLAGLET_SAXS_and_models/SAXS_models_for_BAMLETs_pH12_4C/bam1o6p25_12.png]

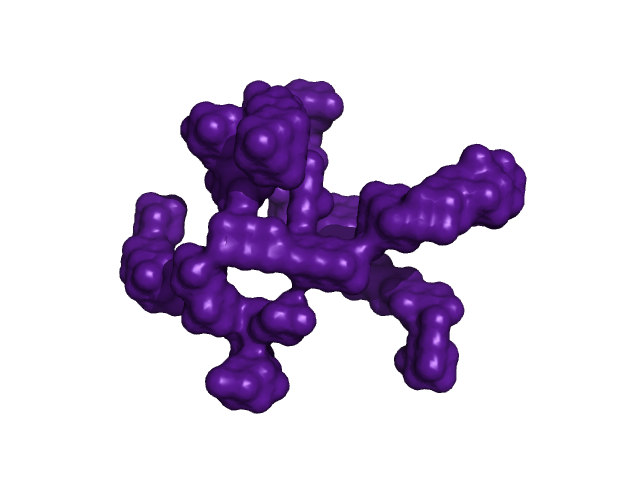

Supplement: S2 Dataset — (TGZ) [file pone.0203003.s003.tgz › BAMLET_and_BLAGLET_SAXS_and_models/SAXS_models_for_BAMLETs_pH12_4C/bam1o10_11.png]

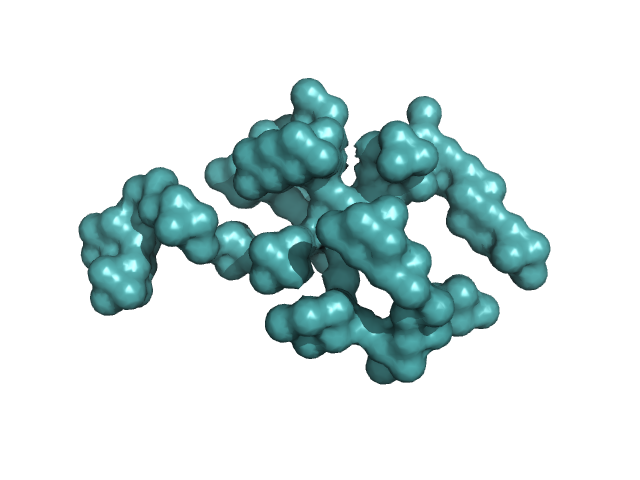

Supplement: S2 Dataset — (TGZ) [file pone.0203003.s003.tgz › BAMLET_and_BLAGLET_SAXS_and_models/SAXS_models_for_BAMLETs_pH12_4C/bam1o5_08.png]

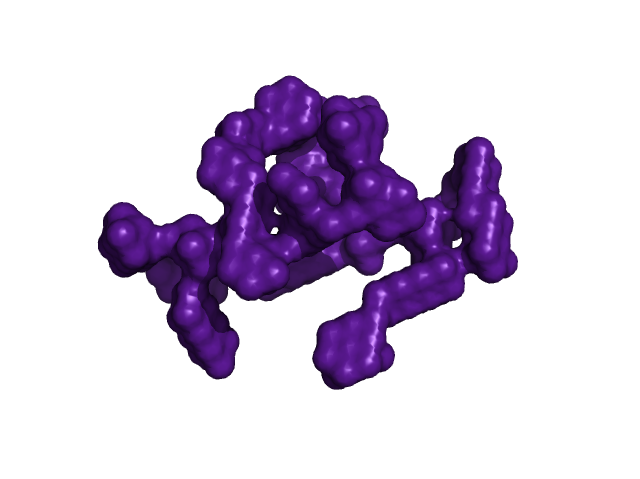

Supplement: S2 Dataset — (TGZ) [file pone.0203003.s003.tgz › BAMLET_and_BLAGLET_SAXS_and_models/SAXS_models_for_BAMLETs_pH12_4C/bam1o10_08.png]

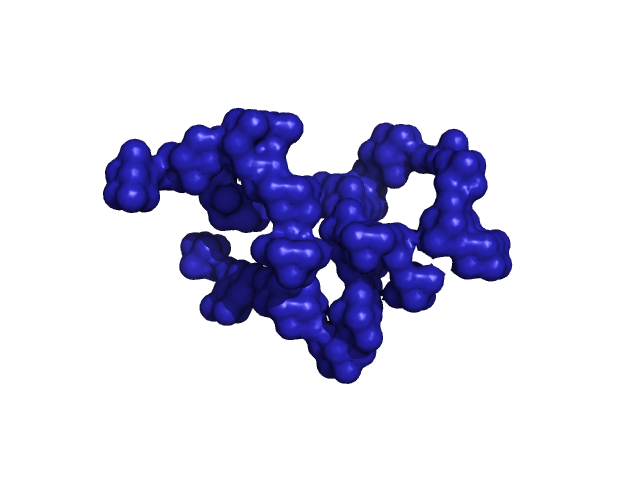

Supplement: S2 Dataset — (TGZ) [file pone.0203003.s003.tgz › BAMLET_and_BLAGLET_SAXS_and_models/SAXS_models_for_BAMLETs_pH12_4C/bam1o7p5_11.png]

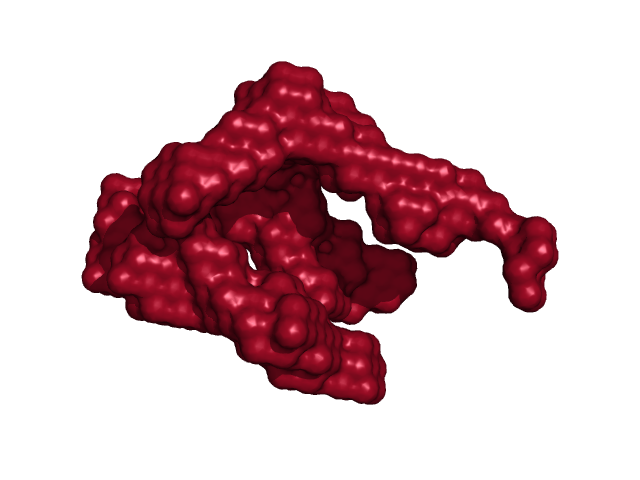

Supplement: S2 Dataset — (TGZ) [file pone.0203003.s003.tgz › BAMLET_and_BLAGLET_SAXS_and_models/SAXS_models_for_BAMLETs_pH12_4C/bam1o20_16.png]

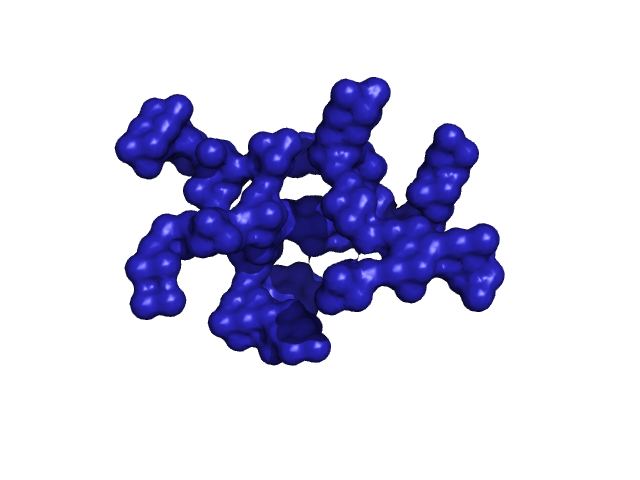

Supplement: S2 Dataset — (TGZ) [file pone.0203003.s003.tgz › BAMLET_and_BLAGLET_SAXS_and_models/SAXS_models_for_BAMLETs_pH12_4C/bam1o7p5_15.png]

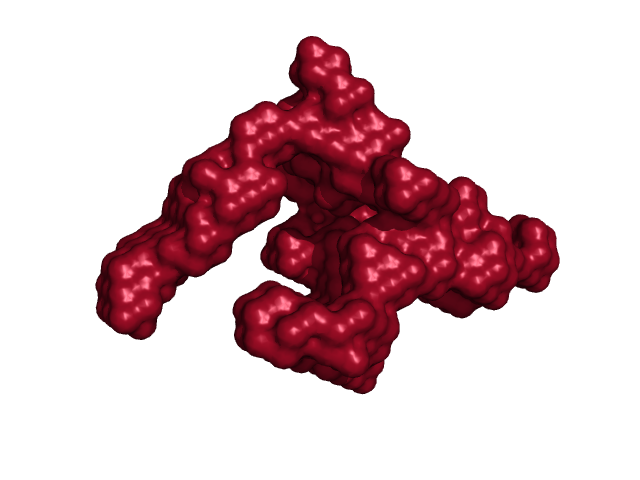

Supplement: S2 Dataset — (TGZ) [file pone.0203003.s003.tgz › BAMLET_and_BLAGLET_SAXS_and_models/SAXS_models_for_BAMLETs_pH12_4C/bam1o20_18.png]

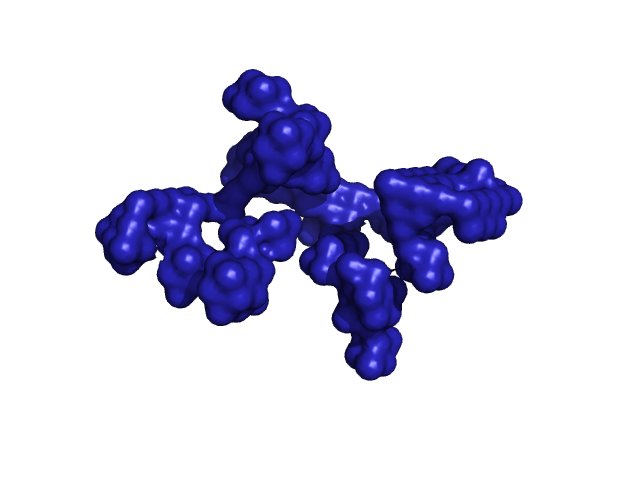

Supplement: S2 Dataset — (TGZ) [file pone.0203003.s003.tgz › BAMLET_and_BLAGLET_SAXS_and_models/SAXS_models_for_BAMLETs_pH12_4C/bam1o7p5_02.png]

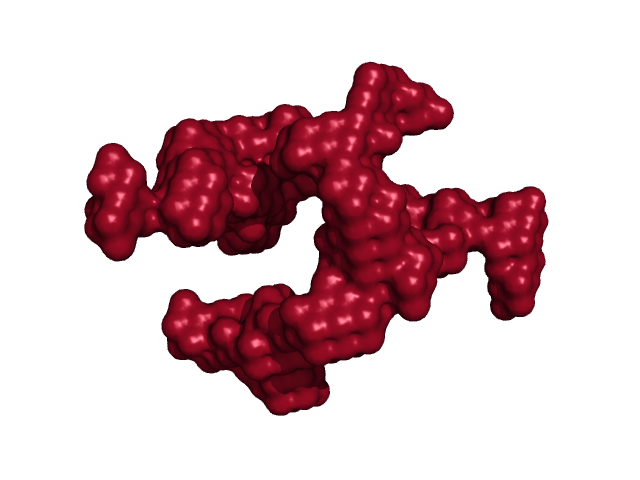

Supplement: S2 Dataset — (TGZ) [file pone.0203003.s003.tgz › BAMLET_and_BLAGLET_SAXS_and_models/SAXS_models_for_BAMLETs_pH12_4C/bam1o20_05.png]

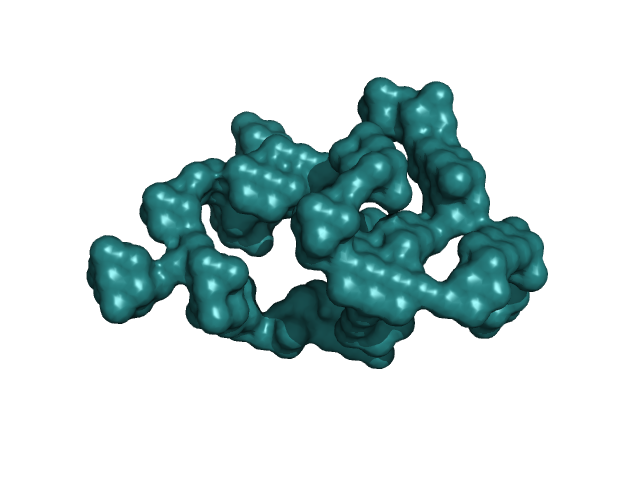

Supplement: S2 Dataset — (TGZ) [file pone.0203003.s003.tgz › BAMLET_and_BLAGLET_SAXS_and_models/SAXS_models_for_BAMLETs_pH12_4C/bam1o5p625_20.png]

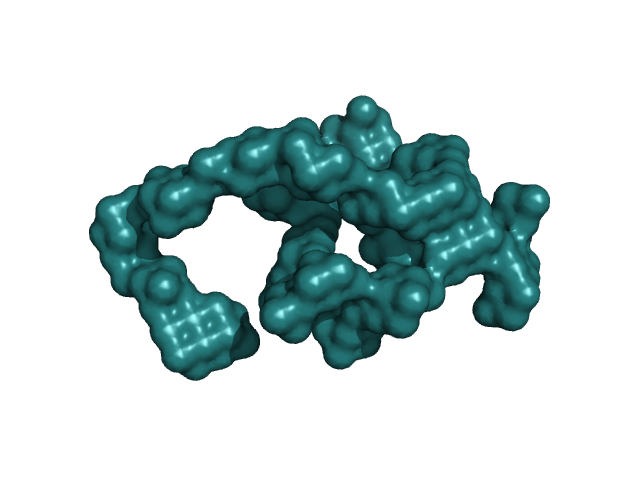

Supplement: S2 Dataset — (TGZ) [file pone.0203003.s003.tgz › BAMLET_and_BLAGLET_SAXS_and_models/SAXS_models_for_BAMLETs_pH12_4C/bam1o5p625_02.png]

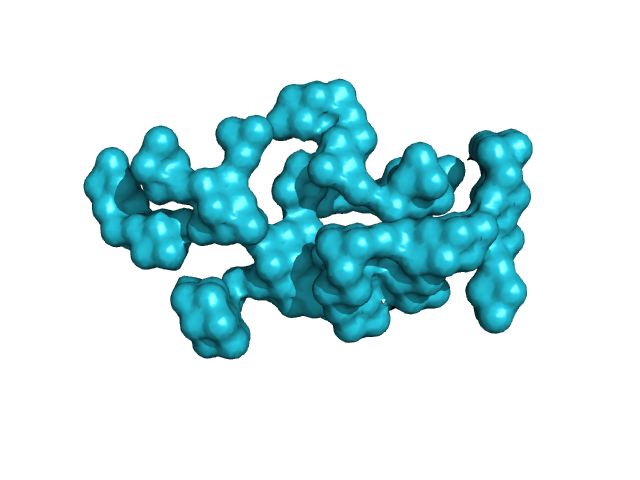

Supplement: S2 Dataset — (TGZ) [file pone.0203003.s003.tgz › BAMLET_and_BLAGLET_SAXS_and_models/SAXS_models_for_BAMLETs_pH12_4C/bam1o2_04.png]

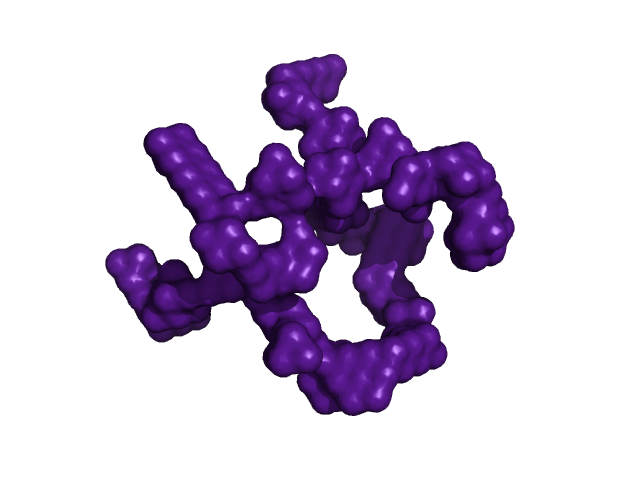

Supplement: S2 Dataset — (TGZ) [file pone.0203003.s003.tgz › BAMLET_and_BLAGLET_SAXS_and_models/SAXS_models_for_BAMLETs_pH12_4C/bam1o10_10.png]

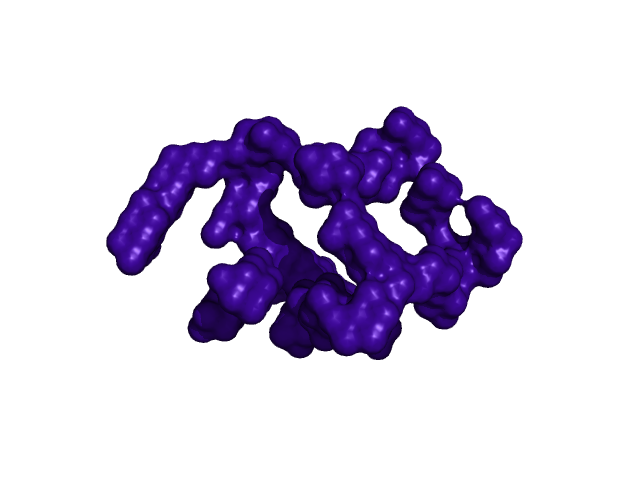

Supplement: S2 Dataset — (TGZ) [file pone.0203003.s003.tgz › BAMLET_and_BLAGLET_SAXS_and_models/SAXS_models_for_BAMLETs_pH12_4C/bam1o8p75_11.png]

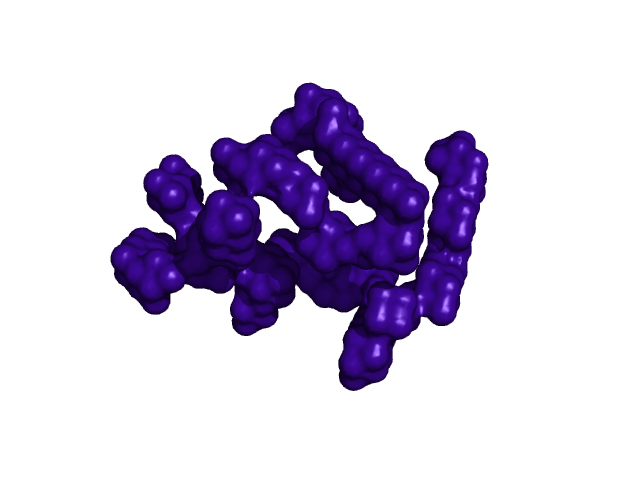

Supplement: S2 Dataset — (TGZ) [file pone.0203003.s003.tgz › BAMLET_and_BLAGLET_SAXS_and_models/SAXS_models_for_BAMLETs_pH12_4C/bam1o8p75_12.png]

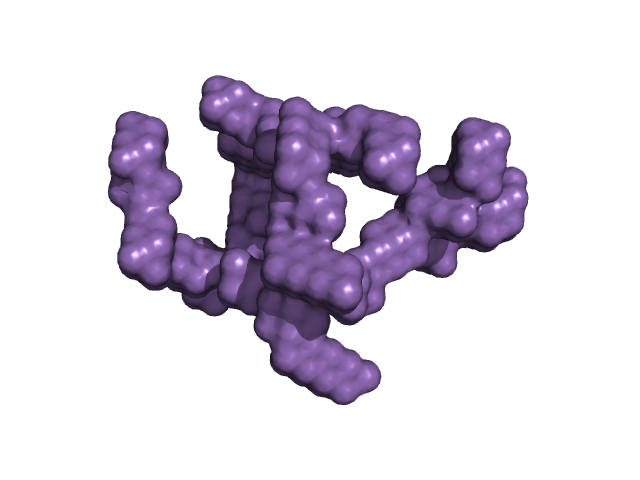

Supplement: S2 Dataset — (TGZ) [file pone.0203003.s003.tgz › BAMLET_and_BLAGLET_SAXS_and_models/SAXS_models_for_BAMLETs_pH12_4C/bam1o12p5_18.png]

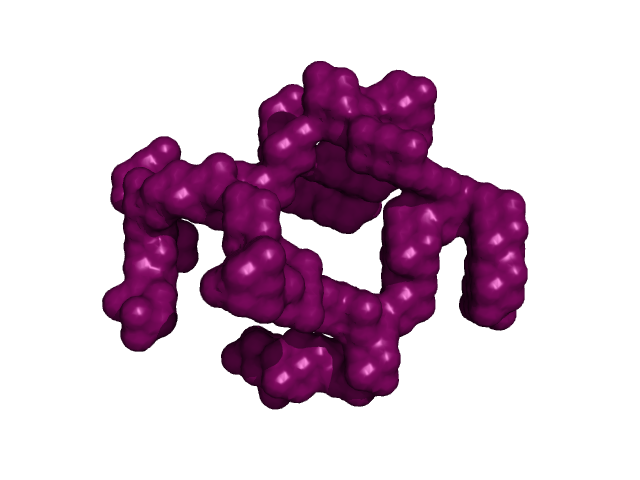

Supplement: S2 Dataset — (TGZ) [file pone.0203003.s003.tgz › BAMLET_and_BLAGLET_SAXS_and_models/SAXS_models_for_BAMLETs_pH12_4C/bam1o15_14.png]

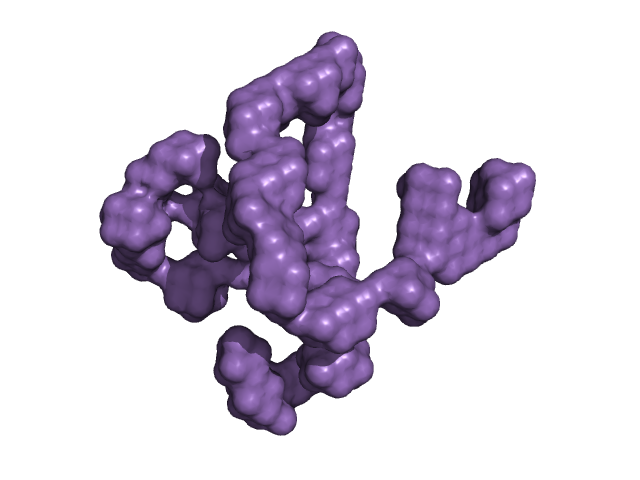

Supplement: S2 Dataset — (TGZ) [file pone.0203003.s003.tgz › BAMLET_and_BLAGLET_SAXS_and_models/SAXS_models_for_BAMLETs_pH12_4C/bam1o12p5_09.png]

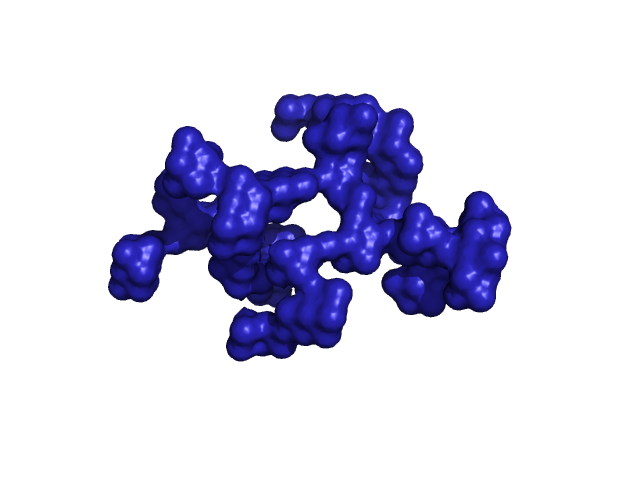

Supplement: S2 Dataset — (TGZ) [file pone.0203003.s003.tgz › BAMLET_and_BLAGLET_SAXS_and_models/SAXS_models_for_BAMLETs_pH12_4C/bam1o7p5_16.png]

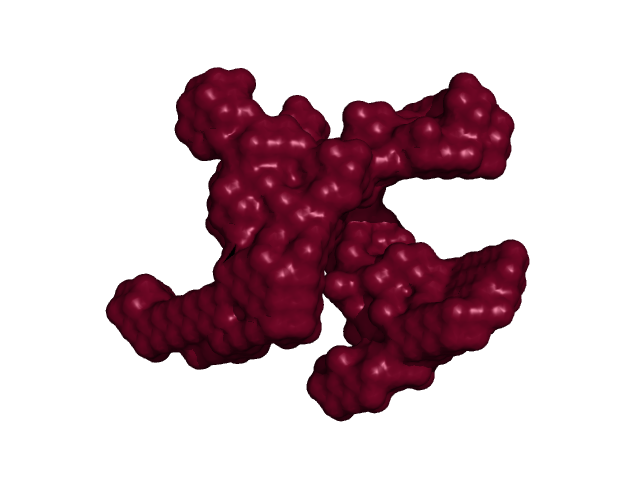

Supplement: S2 Dataset — (TGZ) [file pone.0203003.s003.tgz › BAMLET_and_BLAGLET_SAXS_and_models/SAXS_models_for_BAMLETs_pH12_4C/bam1o17p5_07.png]

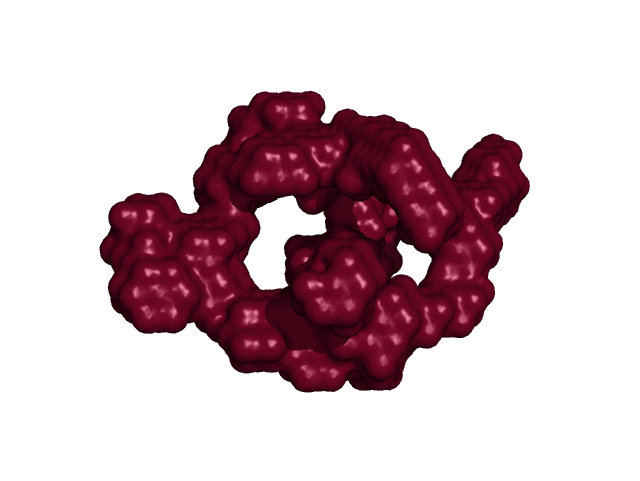

Supplement: S2 Dataset — (TGZ) [file pone.0203003.s003.tgz › BAMLET_and_BLAGLET_SAXS_and_models/SAXS_models_for_BAMLETs_pH12_4C/bam1o17p5_03.png]

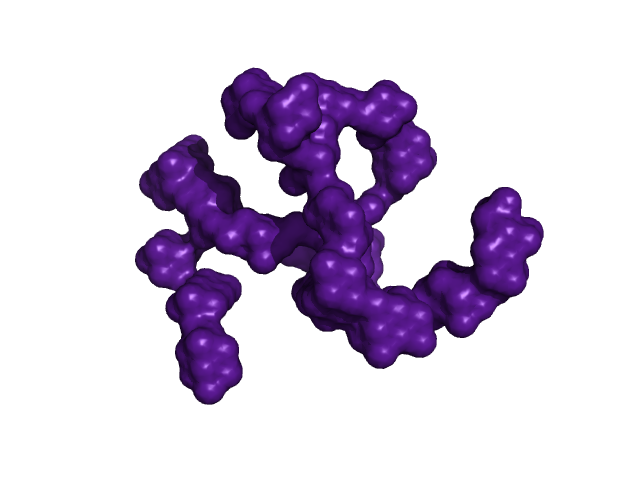

Supplement: S2 Dataset — (TGZ) [file pone.0203003.s003.tgz › BAMLET_and_BLAGLET_SAXS_and_models/SAXS_models_for_BAMLETs_pH12_4C/bam1o10_01.png]

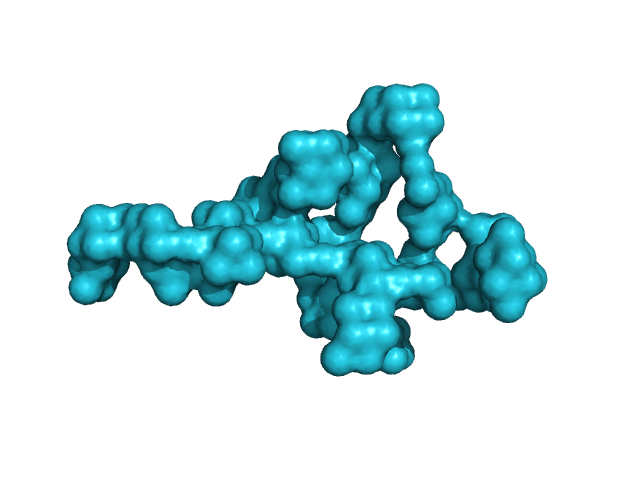

Supplement: S2 Dataset — (TGZ) [file pone.0203003.s003.tgz › BAMLET_and_BLAGLET_SAXS_and_models/SAXS_models_for_BAMLETs_pH12_4C/bam1o2_07.png]

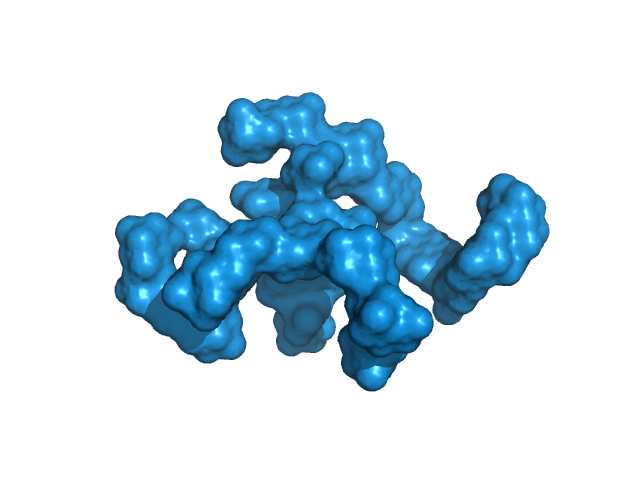

Supplement: S2 Dataset — (TGZ) [file pone.0203003.s003.tgz › BAMLET_and_BLAGLET_SAXS_and_models/SAXS_models_for_BAMLETs_pH12_4C/bam1o6p25_16.png]

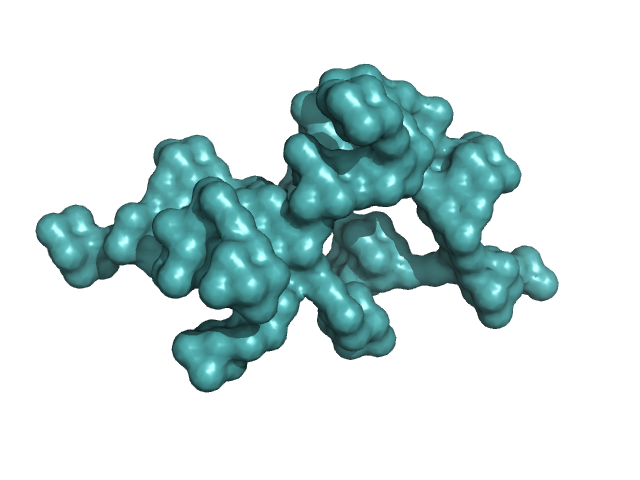

Supplement: S2 Dataset — (TGZ) [file pone.0203003.s003.tgz › BAMLET_and_BLAGLET_SAXS_and_models/SAXS_models_for_BAMLETs_pH12_4C/bam1o5_04.png]

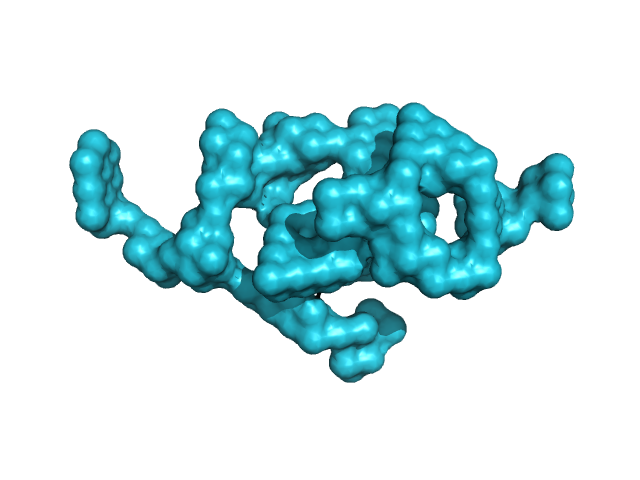

Supplement: S2 Dataset — (TGZ) [file pone.0203003.s003.tgz › BAMLET_and_BLAGLET_SAXS_and_models/SAXS_models_for_BAMLETs_pH12_4C/bam1o2_13.png]
